# Bispecific CAR T cell therapy targeting BCMA and CD19 in relapsed/refractory multiple myeloma: a phase I/II trial

**DOI:** 10.1038/s41467-024-47801-8

**Published:** 2024-04-20

**Authors:** Ming Shi, Jiaojiao Wang, Hongming Huang, Dan Liu, Hai Cheng, Xu Wang, Wei Chen, Zhiling Yan, Wei Sang, Kunming Qi, Depeng Li, Feng Zhu, Zhenyu Li, Jianlin Qiao, Qingyun Wu, Lingyu Zeng, Xiaoming Fei, Weiying Gu, Yuqing Miao, Kailin Xu, Junnian Zheng, Jiang Cao

**Affiliations:** 1 Cancer Institute, Xuzhou Medical University, Xuzhou, 221002 China; 2 Department of Hematology, The Affiliated Hospital of Xuzhou Medical University, Xuzhou, 221002 China; 3 Department of Hematology, The Affiliated Hospital of Nantong University, Nantong, 226000 China; 4 Jiangsu Bone Marrow Stem Cell Institute, Xuzhou, 221002 China; 5 Center of Clinical Oncology, The Affiliated Hospital of Xuzhou Medical University, Xuzhou, 221002 China; 6 Jiangsu Center for the Collaboration and Innovation of Cancer Biotherapy, Xuzhou Medical University, Xuzhou, 221002 China; 7 Department of Hematology, The Affiliated Hospital of Jiangsu University, Zhenjiang, 212000 China; 8 Department of Hematology, The First People’s Hospital of Changzhou, The Third Affiliated Hospital of Soochow University, Changzhou, 213000 China; 9 Department of Hematology, Yancheng No. People’s Hospital, Yancheng, 224006 China

**Keywords:** Clinical trials, Myeloma, Cancer immunotherapy

## Abstract

Despite the high therapeutic response achieved with B-cell maturation antigen (BCMA)-specific chimeric antigen receptor (CAR) T-cell therapy in relapsed and refractory multiple myeloma (R/R MM), primary resistance and relapse exist with single-target immunotherapy. Here, we design bispecific BC19 CAR T cells targeting BCMA/CD19 and evaluate antimyeloma activity in vitro and in vivo. Preclinical results indicate that BC19 CAR specifically recognize target antigens, and BC19 CAR T cells mediate selective killing of BCMA or CD19-positive cancer cells. BC19 CAR T cells also exhibit potent antigen-specific anti-tumor activity in xenograft mouse models. We conduct an open-label, single-arm, phase I/II study of BC19 CAR T cells in 50 patients with R/R MM (ChiCTR2000033567). The primary endpoint was safety. BC19 CAR T cells are well tolerated with grade 3 or higher cytokine release syndrome in 8% of patients and grade 1 neurotoxic events in 4% of patients, which meet the pre-specified primary endpoint. Secondary endpoints include overall response rate (92%), median progression-free survival (19.7 months), median overall survival (19.7 months) and median duration of response (not reached). Our study demonstrates that bispecific BC19 CAR T cells are feasible, safe and effective in treating patients with R/R MM.

## Abstract (web summary)

CAR-T cell therapies targeting BCMA have shown promising responses in patients with multiple myeloma (MM), however primary resistance and relapse are frequently observed. Here the authors report the results of a phase I//II study of bispecific CAR T-cells targeting BCMA and CD19 in relapsed/refractory MM.

## Introduction

Multiple myeloma (MM) is the second most common hematological malignancy characterized by clonal proliferation of plasma cells in the bone marrow or less frequently at extramedullary sites producing monoclonal immunoglobulins^1^. Although the past decade has been marked by significant therapeutic advances, increasing patient survival, MM is still considered incurable and almost all MM patients eventually relapse. Effective and tolerable treatment options for relapsed and refractory (R/R) MM patients remain unsatisfactory with a median overall survival (OS) of 9.3 months in triple-refractory patients and 5.6 months in penta-refractory patients^2^.

Chimeric antigen receptor (CAR) T cell therapies targeting B-cell maturation antigen (BCMA) have shown promising responses and outcomes in heavily pretreated and treatment-refractory MM patients, with 48–100% overall response rates and 6–76% complete response rates^3–10^. However, some patients had no responses to BCMA-targeted CAR T cells or relapsed soon after achieving responses. Downregulation or loss of BCMA expression is observed in 4–33% of progressive patients following CAR T therapy^7,9,11^. One approach to address this problem is to use dual-targeting CAR T cells, as observed in clinical trials using CAR T cells targeting CD19/CD22 for leukemia and lymphoma, CD19/CD20 for lymphoma, and BCMA/CD38 for MM^11–15^.

Previous studies have shown that a small proportion of MM cells express CD19, which are considered to be less-differentiated MM cells or myeloma-like stem cells and related to drug resistance and poor survival^16,17^. Recent studies confirm that CD19 is expressed at an ultra-low density on a fraction of myeloma cells (10.3–80%) in the majority of patients, and CD19^low^ myeloma cells can be eliminated by anti-CD19 CAR T cells^18^. Moreover, administration of anti-CD19 CAR T cells after high-dose melphalan and salvage autologous hematopoietic stem cell transplantation (Auto-HSCT) also showed potential activity in R/R MM^19^. Prompted by these observations, we previously conducted a prospective study and demonstrated that combined infusion of humanized anti-CD19 and anti-BCMA CAR T cells is feasible in patients with R/R MM^20,21^.

In this work, on the basis of these preliminary data, we designed a second-generation bispecific BC19 CAR containing an anti-BCMA single-chain variable fragment (scFv) and a humanized anti-CD19 scFv. Here, we report the preclinical results of bispecific BC19 CAR T cells and the outcomes of patients with R/R MM treated in a Phase I/II trial. 50 patients with R/R MM are administered BC19 CAR T cell infusions. The BC19 CAR T cells are observed to be well tolerated, exhibiting significant clinical efficacy. The overall response rate in this population is 92%, the median progression-free survival is 19.7 months, and the 1-year overall survival rate is 85%. Overall, our study finds that infusion of BC19 bispecific CAR T cells is a viable, safe, and effective strategy for managing patients with R/R MM.

## Results

### Construction and functional validation of mono- or bispecific CAR T cells targeting BCMA or/and CD19 in vitro

**Fig. 1:**
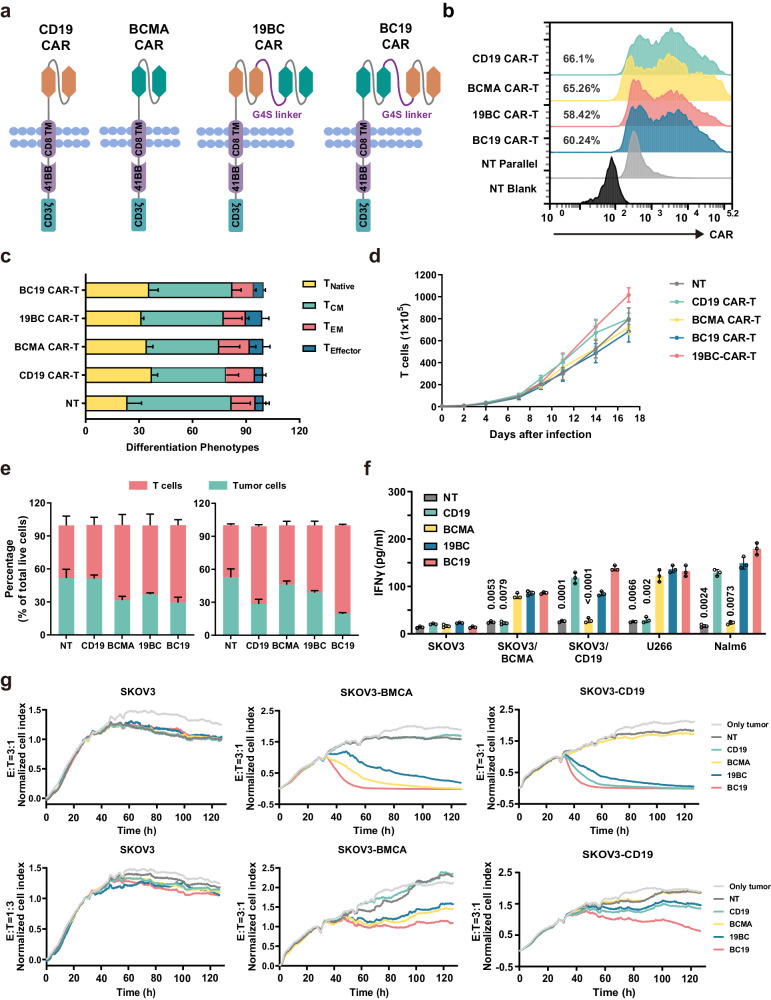
Construction and functional validation of mono- or bispecific CAR T cells targeting BCMA or/and CD19 in vitro*.* **a** Schematic diagram of the CD19/BCMA-targeting CAR constructs used in the current study. Bispecific BC19 or 19BC CAR by connecting an anti-BCMA and anti-CD19 scFv to a second-generation format containing 4-1BB. scFv Single-Chain Variable Fragment, TM Transmembrane Domain. **b** APC-labeled protein L was used to evaluate CAR expression in CD19-, BCMA-, BC19- or 19BC CAR T cells, results of 1 representative experiment are shown (*n* = 3). **c** Naïve, central memory (CM), effector memory (EM), and effector T cells were quantified in the infusion product (*n* = 3, biologically independent samples). **d** The growth curve of CAR T cells during in vitro manufacturing was drawn, results of 1 representative experiment are shown (*n* = 3). **e** CAR T cells were labeled with CTDR and then cocultured with Nalm6 (left, *n* = 3, biologically independent samples) or U266 (right, *n* = 3, biologically independent samples) cells. % CTDR- cells represent CAR T cell-mediated cytotoxicity. **f** ELISA was used to evaluate levels of interferon γ (IFN-γ) in the supernatants (*n* = 3, biologically independent samples). one-way ANOVA followed by Tukey’s multiple comparisons test was used for multiple comparisons with BC19 as a comparison group. **g** SKOV3 (left), SKOV3-BCMA (middle), and SKOV3-CD19 (right) cells were used as target cells to evaluate the specific cytotoxicity of CAR T cells. The E:T ratio is 3:1 (up) or 1:3 (down), results of 1 representative experiment are shown (*n* = 3). Data are presented as mean ± SD, a two-tailed Student *t*-test was performed for two group comparisons. One-way ANOVA analysis of variance was used to determine the statistically significant difference for multiple group comparisons. Statistics: one-way ANOVA followed by Tukey’s multiple comparisons tests (**c,** **d**, **f**). Source data are provided in the Source Data file.

As shown in Fig. 1a, we constructed the bispecific BC19 or 19BC CAR by connecting an anti-BCMA and anti-CD19 scFv into a second-generation format containing 4-1BB. CD19 CAR or BCMA CAR was constructed as reported in our previous studies^20–22^. The expression of the mono- or bispecific CAR in T cells from healthy donors was assessed by flow cytometry. The percentage of BC19 or 19BC CAR-positive T cells in total T cells was approximately 60% (Fig. 1b). The constructed BC19 CAR T cells contained naïve (36%), central memory (42%), effector memory (12%) and effector (5.4%) T cell population, that is similar to the phenotypes of BCMA, CD19 or 19BC CAR-T cells (Fig. 1c). CAR T cell proliferation during ex vivo bio-manufacturing was monitored. The in vitro expansion of BC19 or 19BC CAR T cells was similar to that of BCMA or CD19 CAR T cells or mock T cells (Fig. 1d).

To evaluate the cytotoxicity of CAR T cells to BCMA or CD19-positive hematologic tumor cells, U266 (BCMA^+^) and Nalm6 (CD19^+^) cells were used as target cells. Effector cells were pre-labeled with CTDR and cocultured with target cells for 24 h at a 1:1 Effector: Target (E: T) ratio. The proportion of CTDR cells in coculture represents cytotoxicity mediated by CAR T cells. Our results showed that BC19, 19BC, and BCMA CAR T cells exhibited a greater cytotoxicity and released more IFN-γ compared with CD19 CAR T or mock T cells when U266 was served as target cells (Fig. 1e, f). For evaluating the selective killing of Nalm6 cells induced by CAR T cells, BC19, 19BC, and CD19 CAR T cells showed greater cytotoxicity and secreted more IFN-γ compared with BCMA CAR T or mock T cells. These data indicate that BC19 or 19BC CAR T cells constructed in the current study were able to mediate the selective killing of BCMA or CD19-positive cancer cells. To further verify this, SKOV3 cells overexpressing BCMA (SKOV3-BCMA) or CD19 (SKOV3-CD19) were used as target cells. In Real Time Cellular Analysis (RTCA), neither mono- or bispecific CAR T cells responded to parental SKOV3 cells. However, BC19 and 19BC CAR T cells induced obvious killing effects on SKOV3-BCMA or SKOV3-CD19 cells and released more IFN-γ (Fig. 1f, g).

### Evaluating the antitumor therapeutic ability of BC19 CAR T cells in vivo

**Fig. 2:**
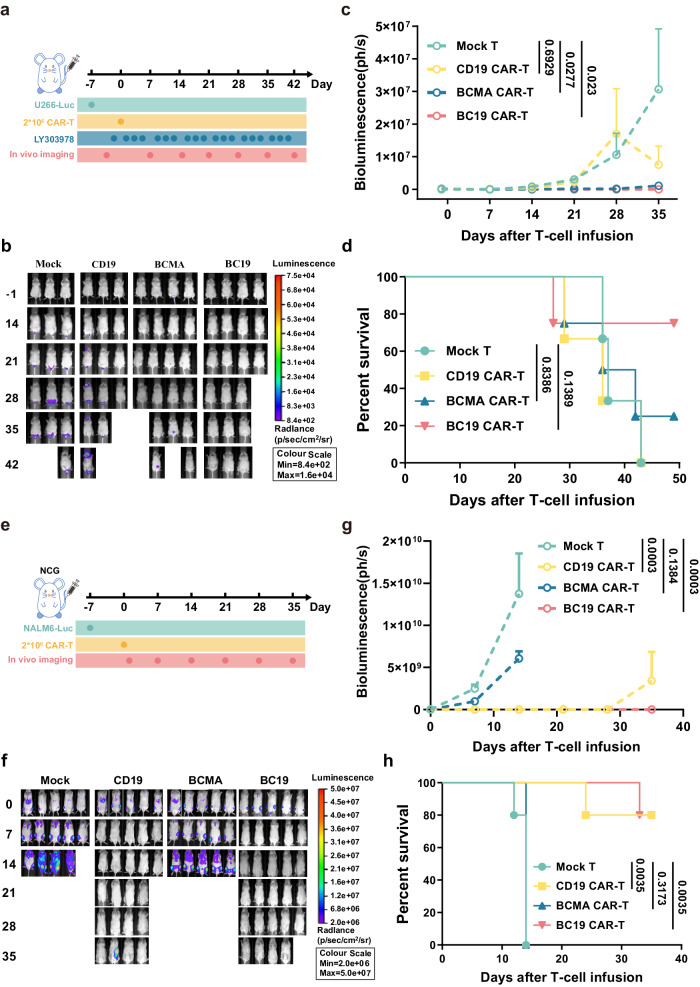
Evaluating antitumor therapeutic ability of BC19 CAR T cells in vivo*.* **a** Schematic diagram of evaluating in vivo anti-BCMA^+^ tumor activities of BC19 CAR T. 2 × 10^6^ U266 cells, endogenously expressing BCMA, were injected into NCG mice via the tail vein on day −7. Γ-secretase inhibitor LY3039478, orally administrated into the mice at 1 mg/kg three times per week, was used to maintain BCMA expression on the surface of U266 cells in mice. Mice were randomly divided into 4 groups and injected with a single dose of 2 × 10^6^ BCMA CAR T, CD19 CAR T, BC19 CAR T, or mock T cells via the tail vein on day 0. **b** In vivo tumor growth was evaluated by luciferase live imaging on days 0, 14, 21, 28, 35, and 42. **c** The bioluminescence value was monitored (Mock T and CD19 CAR T, *n* = 3; BCMA CAR T and BC19 CAR T, *n* = 4). Data are presented as mean ± SEM and analyzed by one-way analysis of variance (ANOVA) followed by Dunnett’s test. **d** Overall survival of the mice was graphically represented as Kaplan–Meier curves and analyzed by using the log-rank test. **e** Schematic diagram of evaluating in vivo anti-CD19^+^ tumor activities of BC19 CAR T. 1 × 10^6^ Nalm6 cells were injected into each NCG mouse on day −7. The mice were treated with 2 × 10^6^ BCMA CAR T, CD19 CAR T, BC19 CAR T, or mock T cells on day 0, respectively. In vivo tumor growth was evaluated (**f**, **g**), and mortality (**h**) of the mice was monitored (*n* = 4). Data are presented as mean ± SD, a two-tailed Student *t*-test was performed for two group comparisons. One-way ANOVA analysis of variance was used to determine the statistically significant difference for multiple group comparisons. Survival of mice was determined using Kaplan–Meier and compared using the log-rank test. Statistics: one-way ANOVA followed by Tukey’s multiple comparisons test (**c**, **g**), log-rank test (**d**, **h**). Source data are provided in the Source Data file.

We have shown that bispecific CAR, BC19, or 19BC, modified T cells were able to mediate specific cytotoxicity to tumor cells expressing BCMA or CD19. BC19 CAR T cells exhibited greater cytotoxic activity at 3:1 or 1:3 E:T ratio compared to 19BC CAR T cells (Fig. 1g), in which BC19 CAR construct was employed in the following in vivo study and clinical trial. To test whether the properties of BC19 CAR T cells found in vitro can be translated into antitumor ability in vivo, a xenograft *mouse* model based on BCMA or CD19-positive tumor cells was employed. Briefly, myeloma U266 cells, grown as suspension cultures, were implanted in NCG mice. For maintaining BCMA expression on the surface of tumor cells, the mice were treated with γ-secretase inhibitor LY3039478^23^. BC19, BCMA, CD19 CAR T, and mock T cells were adoptively transferred into the tumor-bearing mice, and then the inhibitory effects of CAR T cells on U266 cell growth in vivo were monitored by luciferase live imaging assay (Fig. 2a). As shown in Fig. 2b, c, BC19 or BCMA CAR T cell treatment remarkably repressed tumor growth compared to CD19 CAR T or mock T cell treatment. The luciferase activity in the mice of the BC19 CAR T group was undetectable until 42 days after the single dose of CAR T cell treatment, and extended survival was observed in the BC19 CAR T group (Fig. 2d).

To explore the CD19 targeting capacity of BC19 CAR T cells, a xenograft *mouse* model based on Nalm6 cells was employed (Fig. 2e). Figure 2f–h showed that animals in BCMA CAR T and mock T groups succumbed rapidly to progressive disease, while BC19 or CD19 CAR T cell treatment induced tumor remission and prolonged *mouse* survival. These data indicated antigen-specific antitumor activity in BC19 CAR T cells.

### Evaluating binding specificity of the BC19 CAR binder to BCMA or CD19

BCMA scFv-Linker-CD19 scFv, which is the binder of BC19 CAR, was fused with *human* IgG1 Fc or a His tag, respectively (Supplementary Fig. 1a). The two recombinant proteins were expressed and purified, and they appeared at the expected molecular weight in sodium dodecyl sulfate-polyacrylamide gel electrophoresis (SDS-PAGE) (Supplementary Fig. 1b). Both bind to Nalm6 or U266 cells, which express CD19 or BCMA respectively, in a concentration-dependent manner (Supplementary Fig. 1c). SKOV3, SKOV3-BCMA and SKOV3-CD19 cells were used to test the binding specificity of BC19 CAR binders. The flow cytometry results indicated that both BC19 CAR binders could bind to SKOV3-BCMA or SKOV3-CD19 cells, but not SKOV3 cells (Supplementary Fig. 1d). These data suggested that BC19 CAR binder could specifically recognize and bind the endogenous or exogenous target antigens.

### Patient characteristics

From June 2020 to February 2022, 64 patients with R/R MM were screened for eligibility, and 54 patients were initially enrolled and underwent leukapheresis. The manufacturing of BC19 CAR T cells was successful for 100% of patients. Four patients discontinued treatment due to rapid disease progression prior to infusion (Supplementary Fig. 2). Fifty patients finally received BC19 CAR T cell infusions, and the patients’ baseline characteristics are listed in Table 1. The median age of patients was 57 years (range 31–70), and the median time from MM diagnosis to CAR T cell infusion was 29.5 months (range 4–162). A total of 46 patients (96%) had stage II or III disease, 7 patients (14%) had extramedullary disease, and 34 patients (68%) had high-risk cytogenetic profiles, defined by the presence of del(17p), t(4;14), or t(14;16). Patients had a median of 4 lines (range, 2–11) of therapy before enrollment. Among them, 20 patients (40%) had previously received auto-HSCT, and 5 patients (10%) had received prior BCMA, CD19, or GPRC5D-targeted CAR T cell treatment. The proportion of patients exposed or refractory to proteasome inhibitors, immunomodulatory drugs, and CD38 antibodies is presented in Table 1. Two patients (4%) underwent auto-HSCT for consolidation at 2.3 months and 3.8 months after CAR T cell therapy, respectively. The other patients received no further consolidation therapy.

**Table 1:** Characteristics of the patients

	Median (range) or No. (%)		Median (range) or No. (%)
**Age, year**	57 (31–70)	**Bortezomib**	
**Gender**		Exposed	50 (100)
Male	22 (44)	Refractory	50 (100)
Female	28 (56)	**Ixazomib**	
**ECOG performance status**		Exposed	18 (36)
0	22 (44)	Refractory	16 (32)
1	23 (46)	**Previous immunomodulatory drugs**	
2	5 (10)	**Lenalidomide**	
**Monoclonal type**		**Pomalidomide**	
IgG	25 (50)	Exposed	7 (14)
IgA	14 (28)	Refractory	6 (12)
IgD	2 (4)	**Thalidomide**	
λ light chain	6 (12)	Exposed	23 (46)
κ light chain	3 (6)	Refractory	20 (40)
**International Staging System**		**Previous anti-38 monoclonal antibodies**	
I	4 (8)	**Daratumumab**	
II	18 (36)	Exposed	9 (18)
III	28 (56)	Refractory	9 (18)
**Median time since diagnosis, months**	29.5 (4–162)	**Previous ASCT**	20 (40)
**Extramedullary plasmacytomas**	7 (14)	**Previous CAR T cell infusion**	5 (10)
**High-risk cytogenetic**	34 (68)	**Penta-drug exposed**	4 (8)
**Bone marrow plasma cellså 50%**	11 (22)	**Double-refractory disease**	50 (100)
**Detectable CD19 expression**	5 (10)	**Triple-refractory disease**	9 (18)
**Tumor BCMA expression** ≥ **50%**	30(60)	**Penta-refractory disease**	4 (8)
**Previous therapies**	4 (2–11)	**Refractory to the last line of therapy**	40 (80)
**Previous proteasome inhibitors**		**Relapse after the last treatment**	10 (20)

Eastern Cooperative Oncology Group (ECOG) performance‑status scores range from 0 to 5, with higher scores indicating greater disability.

BCMA denotes B‑cell maturation antigen and ASCT autologous stem cell transplantation.

The extramedullary disease was defined as soft-tissue masses spreading outside the bone marrow.

A high tumor burden was defined as at least 50% CD138-positive plasma cells in bone marrow.

High‑risk cytogenetic abnormalities included amplification 1q21, deletion 17p, deletion 13q, t(4;14), t(11; 14), and t(14; 16).

Double‑refractory disease was refractory to an immunomodulatory agent and a proteasome inhibitor.

Triple‑refractory disease was refractory to an immunomodulatory agent, a proteasome inhibitor, and an anti‑CD38 monoclonal antibody.

Penta‑refractory disease was refractory to lenalidomide, pomalidomide, bortezomib, Ixazomib, and daratumumab.

Fonts in bold represent first-level metrics, fonts without bold represent second-level metrics.

### Safety

The most common adverse events that occurred acutely after CAR T cell infusion were hematologic toxicity and CRS (Supplementary Tables 1 and 2). All 50 patients had hematological adverse events, including neutropenia in 100% of patients, leukopenia in 100%, anemia in 94% and thrombocytopenia in 88%. Grade 3–4 hematological adverse events were neutropenia (49 [98%] of 50 patients), leukopenia (48 [96%]), thrombocytopenia (33 [66%]), and anemia (32 [64%]). Patients with grade 3–4 cytopaenia events after day 0 of BC19 CAR T cell infusion recovered to grade 2 or less by day 28 for leukopenia (30 [60%]), neutropenia (27 [54%]), anemia (14 [28%]), and thrombocytopenia (8 [16%]).

CRS occurred in 46 (92%; 95% CI, 81%−98%) of all 50 patients, with grade 3 or higher in 4 patients (8%; 95% CI, 2%−20%). The median time to onset of CRS was 7 days (range 1–24), and the median duration was 3 days (range 1–21). Two patients (4%; 95% CI, 0.5%−14%) had grade 1 neurotoxic events. No grade 3–5 neurotoxic effects were observed. Six patients (12%) received both tocilizumab and glucocorticoids. 19 patients (38%) received tocilizumab, and 16 (32%) received glucocorticoids (Supplementary Table 3). One patient with CR developed hemophagocytic lymphohistiocytosis (according to the diagnostic criteria proposed by Neelapu et al.^24^) on day 19 after grade 2 CRS, and eventually died of septic shock and gastrointestinal bleeding.

Late adverse events (beyond 3 months after CAR T cell infusion) were rare, except for B-cell aplasia, hypogammaglobulinemia, and infections (Supplementary Tables 2 and 4). Forty-five patients developed B-cell aplasia, with a median duration of 138 days (range, 57–461) post-infusion. Hypogammaglobulinemia was found in 20% (10/50) of patients before CAR T cell therapy and in 51% (23/45) of the responders beyond 3 months after BC19 CAR T cell infusion. 14 of 49 patients (29%) experienced prolonged grade 3 or higher cytopenia beyond 3 months after CAR T cell infusion.

Of the 50 patients, 42 (84%) received granulocyte-colony stimulating factor (G-CSF) to treat neutropenia after CAR-Ts infusion. 17 (34%) patients received antifungal prophylaxis due to neutropenia. 42 (84%) patients received antiviral prophylaxis. 16 (32%) patients received anti-Pneumocystis prophylaxis and we did not find patients with Pneumocystis infections during follow-up. In the first three months after CAR-Ts infusion, 30 (60%) patients had an infection, with the majority being mild to moderate in severity (20/30; 66.7%). The most common microorganism was bacterial (*n* = 9), followed by fungal (*n* = 3) and viral (*n* = 1). Of the 49 patients who survived for more than 3 months, 19 (39%) had 26 infections beyond 3 months, including 10 (20%) had 11 severe infections (defined as grade 3 or higher). Seventeen patients (35%) had lung infections, and one (2%) had colonitis. One patient (2%) had herpes zoster, 1 patient (2%) had bacteremia, and eventually died of bacteremia. All infections were controlled with proper and prompt treatment except one with grade 5 infection.

At the cutoff date, 8 patients (16%) died during follow-up (Supplementary Fig. 3). Four patients (8%) died from disease progression or associated complications (No. 13, 17, 33 and 35). Patient 2 attained an sCR in month 1.8, but he died of gastrointestinal bleeding at 19.7 months due to persistent thrombocytopenia. Patient 26 achieved VGPR at 2.3 months after BC19 CAR T treatment, followed by consolidation therapy with auto-HSCT, and died of acute intracranial hemorrhage at 9.1 months. The other 2 patients died while maintaining the response as described above.

### Efficacy

Of the 50 patients assessable for efficacy, 46 (92%; 95% CI, 81%−98%) achieved an overall response (PR or better) to BC19 CAR T cells, including 18 (36%) sCRs, 12 (24%) CRs, 9 (18%) VGPRs, and 7 (16%) PRs (Table 2). Four patients (8%) had stable disease as the best response. The median time to first PR or better was 23.5 days (range, 14–30), and the median time to best response was 1.9 months (range 0.5−6.0). Six of 7 patients (86%; 95% CI, 42%−100%) with extramedullary disease achieved an overall response. Of the five patients who received prior CAR T cell therapy, two achieved sCR, one achieved PR, and the other two had SD.

**Table 2:** Patients’ response to BC19

	Patients (*n* = 50)
Overall response	
Number of patients with a response^a^	46
Proportion of patients with a response, % (95% CI)	92 (81–98)
Best overall response	
Stringent complete response	18 (36)
Complete response	12 (24)
MRD-negative complete response or stringent complete response^b^	22 (44)
Very good partial response	9 (18)
Partial response	7 (14)
Stable disease	4 (8)
Median time to first response, days	23.5 (14–30)
Median time to best response, months	1.9 (0.5–6.0)
Median time to complete response or better, months	2.1 (0.5–6.0)
MRD negativity	
Number of patients with a response who could be evaluated for MRD	39 (85)
Number of patients evaluable for MRD negativity	34 (83)
Median time to MRD negativity, months	0.5 (0.5–2.1)

*MRD* minimal residual disease.

Data are *n* (%) or median (IQR), unless otherwise specified.

^a^Sum of all patients who achieved stringent complete response, complete response, very good partial response, and partial response.

^b^MRD negativity was achieved in 22 (88%) of 25 patients with complete response or stringent complete response who were evaluable for MRD assessment.

Of all patients, 41 had available MRD detection. Thirty-four patients (83%; 95% CI, 68%−93%) achieved MRD negativity, including 22 with CR or sCR (Table 2). The median time for patients with MRD-positive status turned negative after CAR T cell infusion was 0.5 months (range 0.5−2.1). The timing of MRD negativity was not related to the depth of response (Supplementary Fig. 4).

Univariate analyses showed that ORRs were consistent across key covariates, including disease stage, high-risk cytogenetic profile, extramedullary disease, baseline tumor burden, BCMA expression, CD19 detection, number of previous therapy lines, and time since diagnosis. Patients who had received prior CAR T cell treatment had lower ORR (Supplementary Fig. 5).

**Fig. 3:**
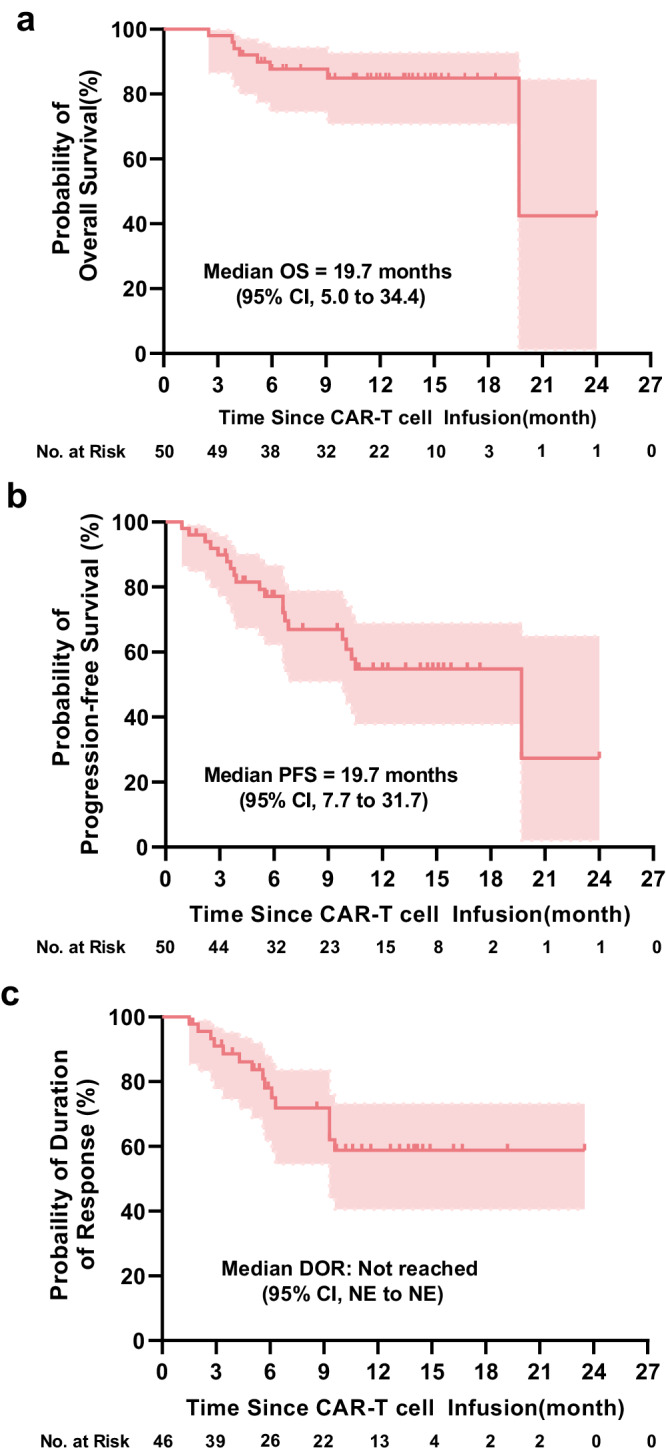
Kaplan–Meier analysis of the overall survival (OS), progression-free survival (PFS), and duration of response (DOR). **a** Kaplan–Meier curves of OS in all 50 patients. **b** Kaplan–Meier curves of PFS in all 50 patients. **c** Kaplan–Meier curves of DOR in 46 patients with a partial or better response. Tick marks indicate the time of data censoring at the last follow-up. NE not estimated. DOR, PFS, and OS of patients were determined using Kaplan–Meier and compared using the log-rank test. Source data are provided in the Source Data file.

At a median follow-up of 11 months (range 2.3–24), 27 of the 46 patients (59%; 95% CI, 43%−73%) with PR or better had ongoing responses. 15 of the 46 patients (33%; 95% CI, 20%−48%) had a relapse or progression during the follow-up, of which 8 of the 30 patients with CR or better and 7 of the 16 patients with VGPR or PR (Supplementary Fig. 3). Of the 15 patients who relapsed or progressed with available BCMA and CD19 detection, 1 (7%) relapsed with BCMA-negative myeloma cells and 14 (93%) relapsed with BCMA-positive myeloma cells. CD19 expression of myeloma cells from all 15 patients was negative. Median OS and PFS for all 50 patients were 19.7 months (95% CI, 5.0−34.4) and 19.7 months (95% CI, 7.7−31.7), respectively (Fig. 3a, b). Median DOR for 46 patients with PR or better was not reached (Fig. 3c). And the 1-year PFS, OS, and DOR rates were 55% (95% CI, 38%−69%), 85% (95% CI, 71%−93%) and 59% (95% CI, 41%−73%), respectively. CR or better patients 12 months PFS and OS rates were 66% (95 CI%, 45%−81%) and 90% (95% CI, 72%−97%), respectively.

### Expansion and persistence

In patients with assessable CAR transgenes, CAR-BC19 amplification in peripheral blood peaked at a median of 12 days (range, 4–25) after infusion followed by a gradual decrease. The rates of detectable CAR-BC19 at 3, 6, 9, and 12 months were 86%, 62%, 44%, and 28%, respectively (Supplementary Fig. 6). Through post-hoc analysis, we found that peak and cumulative levels of CAR-BC19 transgene during the first 28 days in patients with responses were significantly higher than those without responses (Supplementary Fig. 7a). However, the peak levels and cumulative levels of CAR transgene were not associated with the severity of CRS (Supplementary Fig. 7b).

## Discussion

CAR T cell infusion targeting BCMA is currently the most popular CAR T cell therapy for treating R/R MM. Despite the significant therapeutic response achieved with BCMA CAR T cell therapy, MM remains a highly heterogeneous tumor, and thus mono-targeted CAR T therapy may be susceptible to immunologic escape from BCMA-negative cells over the long term^25^. Our previous study showed that combined infusion of humanized anti-CD19 and anti-BCMA CAR T cells achieved 92% ORR in 62R/R MM patients, and 60% of patients achieved CR or better, which is consistent with previous BCMA CAR T cell results^20^. More importantly, the estimated median duration of response was 20.3 months and the median PFS was 18.3 months in R/R MM patients who received combined CAR T cells infusion. Compared with patients who received the same anti-BCMA CAR T cell product alone, patients who received combined CAR T cells seemed to have longer DOR and PFS. On the other hand, the simultaneous preparation of autologous anti-CD19 and anti-BCMA CAR T cells will increase the difficulty and cost of quality control of cell products. Inspired by these findings, and in order to better control the quality of cell products and reduce production costs, we constructed bispecific BC19 CAR T cells targeting BCMA and CD19 and preliminarily verified their safety and efficacy in R/R MM patients.

BC19 CAR T cells were well tolerated and displayed significant clinical activities in patients with R/R MM who received a median of 4 prior lines of therapy compared with other antimyeloma treatments, with an ORR of 92%, a CR and sCR rate of 62%, an MRD-negative rate of 83%, median PFS of 19.7 months and a 1-year OS rate of 85%. In a study of idecabtagene vicleucel, a BCMA-targeting CAR T cell therapy, 73% of patients with more heavily pretreated R/R MM (median of 6 previous regimens) had a response with 33% achieving a CR or better, and median PFS was 8.8 months^9^. In a study of ciltacabtagene autoleucel, a biepitope BCMA-targeted CAR T cell therapy, ORR rate was 97.9% in heavily pretreated R/R MM patients (median of 6 previous therapies) with 82.5% sCR rate, and median PFS was not reached^3^. Although the patients in our study were less heavily pretreated than patients in the studies of KarMMa and CARTITUDE-1 due to the limited availability of innovative drugs, especially CD38 monoclonal antibody in China, BC19 CAR T cells exhibited a promising responsive rate, depth, and durability in patients with R/R MM.

CRS and neurotoxicity were the most common adverse events following CAR T cell infusion. In our study, CRS occurred in 46 patients and was mostly grades 1 or 2, and 8% of whom had grades 3–5, which is comparable to that observed in other BCMA-targeted CAR T cell therapy^3–10^. The CRS-associated symptoms were resolved in all patients with supportive measures, including tocilizumab and glucocorticoids. Reversible neurotoxicity was only observed in 2 patients (4%) after BC19 CAR T cells infusion, and the incidence rate of neurotoxicity was lower compared to data reported by other studies. This safety profile might be attributable to the low weight-based dose of infused CAR T cells and the early use of tocilizumab and glucocorticoids.

In addition to CRS and neurotoxicity, hematotoxicity has drawn increasing recognition in clinical practice^26^. With tisagenlecleucel for R/R B-cell acute lymphoblastic leukemia (ALL) in children and young adults, 41% patients had grade 3–4 thrombocytopenia, and 53% had grade 3–4 neutropenia that were not resolved by 30 days following CAR T cell infusion^27^. With axicabtagene ciloleucel for large B-cell lymphoma, grade 3–4 cytopenias were observed in 15 (48%) of 31 patients on day 30, including neutropenia in 9 (29%) patients, anemia in 5 (16%) and thrombocytopenia in 13 (42%) patients^28^. With idecabtagene vicleucel for R/R MM, 52 (41%) patients had grade 3–4 neutropenia, and 62 (48%) had grade 3–4 thrombocytopenia on day 30^9^. In another BCMA-directed CAR T cell (ciltacabtagene autoleucel) trial for R/R MM, 24 (25%) patients had grade 3–4 neutropenia, and 1 (1%) had grade 3–4 thrombocytopenia at day 30^29^. Similarly, grade 3–4 cytopenias were observed on day 28 in our study, including neutropenia in 22 (44%) patients, anemia in 18 (36%), and thrombocytopenia in 25 (50%) patients. The phenomenon of cytopenia after CAR T cell therapy is poorly understood with various proposed hypotheses. Further studies will be conducted to determine whether cytopenia affects the long-term survival of patients with increased sample size and longer follow-up time.

Some limitations in this study should be considered. The patients involved in this study were only from China. Some patients were less heavily pretreated, and were not exposed to immunomodulatory drugs (IMiDs) or CD38 monoclonal antibody, than some of the previous CAR T studies. Additionally, the follow-up time was relatively short. Further study will be required to assess the efficacy and adverse events of long term.

Taken together, these preliminary results indicate that bispecific BC19 CAR T cells were feasible, safe, and effective in patients with R/R MM. Despite the high therapeutic response achieved with BC19 CAR T cell therapy, a larger sample size and longer follow-up time are required in future prospective and multicenter clinical trials to explore the impact of BC19 CAR T cell therapy on long-term outcomes.

## Methods

The study was sponsored and designed by the Department of Hematology, the Affiliated Hospital of Xuzhou Medical University, and was approved by the Medical Ethics Committee of Xuzhou Medical University Affiliated Hospital (Approved number: XYFY2020-KL062-01). The enrollment was from June 08, 2020 (first patient) to February 02, 2022 (last patient) and all patients enrolled and treated in this trial signed written informed consents prior to participation. The trial was registered on Chictr.org.cn, number ChiCTR2000033567. All clinical investigations were conducted in accordance with the Declaration of Helsinki principles. The study protocol is available in the Supplementary Information file.

All studies with mice were approved by the Ethics Committee for Experimental Animals of Xuzhou Medical University. The mice were maintained in a specific pathogen-free (SPF) environment, in accordance with the requirements of the Ethics Committee for Experimental Animals of Xuzhou Medical University.

### Clinical trial design

We leveraged our experience with anti-BCMA and humanized anti-CD19 CAR T cells to develop two dual-targeting, second-generation bispecific CARs with a 4-1BB costimulatory domain (BC19 and 19BC CARs). A preclinical study was conducted to evaluate proliferation, cytotoxicity, cytokine production, and antitumor activity of BC19, 19BC, CD19, and BCMA CAR T cells. Next, we performed a single-arm, multicenter, phase I/II clinical trial of BC19 CAR T cells in patients with R/R MM. We conducted an open-label, single-arm, multicenter, phase I/II trail of BC19 CAR T cells in patients with R/R MM registered with the Chinese Clinical Trial Registry (link: https://www.chictr.org.cn/, number: ChiCTR2000033567, date: 06/05/2020). To be eligible for participation in this study, patients had to be: (1) Less than 70 years old; (2) Meet the diagnostic criteria for R/R MM defined by the International Myeloma Working Group (IMWG); (3) Patients had experienced relapse or were refractory to at least 2 prior lines of therapy, including a proteasome inhibitor and an immunomodulatory drug; and (4) Patients had measurable disease and adequate performance status and organ function, with an Eastern Cooperative Oncology Group (ECOG) score ≤2. Positive BCMA expression on MM cells was required to be confirmed by flow cytometry regardless of whether CD19 was expressed, but no pre-specified level of expression was required. Female patients had to be human chorionic gonadotropin-negative, with no plans for pregnancy within 6 months of treatment. Patients with mental or psychological illnesses, severe allergies, or a history of severe allergies (especially those who were allergic to interleukin 2[IL-2]) were excluded. Detailed criteria for inclusion and exclusion are provided in supplementary information.

### Cell lines

*Human* B-cell precursor leukemia cell line Nalm6, *human* myeloma cell line U266 and *human* ovarian cancer cell line SKOV3 are obtained from ATCC and cultured according to standard protocols. Nalm6 or U266 cells stably expressing luciferase (Nalm6-luc or U266-luc) and SKOV3 cells stably expressing CD19 (SKOV3-CD19) or BCMA (SKOV3-BCMA) were constructed in our lab. All cell lines’ authenticity was confirmed through STR (Short Tandem Repeat) profiling, and routine mycoplasma testing was performed using a mycoplasma detection kit.

### Viral vector construction

The construction of the anti-CD19 or anti-BCMA chimeric antigen receptor (CAR) has been reported by us^20,21^. The bispecific BCMA/CD19 (BC19)- and CD19/BCMA (19BC)-CARs containing anti-BCMA single-chain variable fragment (scFv) and anti-CD19 scFv in tandem, linked by GGGGS×4, were constructed in this study, respectively. The lentiviral transfer plasmid contains an anti-CD19, anti-BCMA, anti-BC19 or anti-19BC domain, *human* CD8α hinge and transmembrane region, and *human* 4-1BB and *human* CD3ζ signaling moieties. The lentivirus was manufactured by Genechem Co., LTD (Shanghai, China).

### CAR T cell production

Primary peripheral blood mononuclear cells (PBMCs) were isolated from the peripheral blood of healthy donors and patients with R/R MM. All PBMC donated by healthy donors were approved by the Medical Ethics Committee of the Affiliated Hospital of Xuzhou Medical University, and all donors signed informed consent forms. T lymphocytes were isolated using EasySep™ *Human* T Cell Isolation Kit (STEMCELL) according to manufacturing instructions. The procedure for producing CAR T cells has been described in our previous studies^20,21^.

### CAR T cell proliferation assay and phenotype identification

5 × 10^5^ *human* T cells were infected by the lentivirus mentioned above. To draw cell growth curves, live T cells were counted using trypan blue staining and Automated Cell Counter (Thermo Fisher, Countess™ 3). Expression of CD19-, BCMA-, BC19- or 19BC CAR was evaluated by Biotinylated protein L (Acro Biosystems, Cat: RPL-P814R, Lot: BL11R-76EF1-GY,1:400 dilution) and APC--conjugated Streptavidin (Biolegend, Cat: 405207, Lot: B388667, 1:100 dilution). PE-labeled anti-CD45RA (Biolegend, Clone: HI100, Cat: 304107, Lot: B378521, 1:100 dilution) and PE-Cy7-labeled anti-CD62L (Biolegend, Clone: DREG-56, Cat: 304821, Lot: B373156, 1:100 dilution) were employed to determine phenotypes of infusion products of CAR T, including naïve (CD45RA^+^ CD62L^+^), central memory (CD45RA^−^ CD62L^+^), effector memory (CD45RA^−^ CD62L^−^), and effector (CD45RA^+^ CD62L^−^) T cells.

### CAR T cell killing assay

For evaluating the in vitro cytotoxicity of CAR T cells, including CD19-, BCMA-, BC19-, and 19BC CAR T cells, CAR T cells were labeled with CellTracker Deep Red (Thermo Fisher Scientific, Cat: C34565, Lot: 1987253, CTDR, 1:40,000 dilution) for 25 min at 37 °C and then washed with phosphate-buffered saline (PBS) twice. The labeled CAR T cells were cocultured with Nalm6 or U266 cells for 24 h and analyzed using FACScan flow cytometer and CellQuest software (BD Biosciences). % CTDR- cells represent CAR T cell-mediated cytotoxicity. Experiments were repeated at least twice.

To further evaluate the specific cytotoxicity of CAR T cells, Real Time Cellular Analysis (RTCA) was employed. SKOV3, SKOV3-CD19, and SKOV3-BCMA cells were used as target cells, being added to each well of 96-well E-Plate (Agilent Technologies). After proliferation was monitored for 23 h, either mock or CAR T cells were added. T cell numbers were varied to achieve E:T ratios of 3:1 or 1:3. Data were collected using xCELLigence (Agilent, RTCA DP). Experiments were repeated at least twice.

### Enzyme-linked immunosorbent assay (ELISA)

The level of interferon γ (IFN-γ) in the supernatants of the coculture system was detected using the ELISA kits (R&D Systems) following the manufacturer’s instructions. Experiments were performed three times independently.

### Antibodies

Biotinylated protein L (Acro Biosystems, Cat: RPL-P814R, Lot: BL11R-76EF1-GY,1:400 dilution) and APC--conjugated Streptavidin (Biolegend, Cat: 405207, Lot: B388667, 1:100 dilution). PE-labeled anti-CD45RA (Biolegend, Clone: HI100, Cat: 304107, Lot: B378521, 1:100 dilution) and PE-Cy7-labeled anti-CD62L (Biolegend, Clone: DREG-56, Cat: 304821, Lot: B373156, 1:100 dilution); CellTracker Deep Red (Thermo Fisher Scientific, Cat: C34565, Lot: 1987253, CTDR, 1:40,000 dilution).

### In vivo xenograft *mouse* model

For establishing MM xenograft *mouse* models, 6- to 8-week-old male NCG (NOD/ShiLtJGpt-*Prkdc*^em26Cd52^*Il2rg*^em26Cd22^/Gpt) mice were purchased from GemPharmatech Co., Ltd with a production license number SCXK (Su) 2023-0009. This study was approved by the Ethics Committee for Experimental Animals of Xuzhou Medical University. The mice were maintained in a specific pathogen-free (SPF) environment, in accordance with the requirements of the Ethics Committee for Experimental Animals of Xuzhou Medical University. They were subjected to a 12-h light-dark cycle, provided ad libitum access to food and water, and allowed to acclimate for one week. A total of 0.1 mL of the U266 cell suspension (2 × 10^7^ cells/mL) was injected via the tail vein on day −7. In order to maintain BCMA expression on the surface of U266 cells in vivo, γ-secretase inhibitor LY3039478 was employed^23^. LY3039478 was formulated in 1% carboxymethylcellulose and 0.25% Tween 80, suspended by probe and water bath sonication, and then orally administered to mice at 1 mg/kg three times per week. Mice were randomly divided into 4 groups (*n* = 3 or 4) and injected with a single dose of 2 × 10^6^ BCMA CAR T, CD19 CAR T, BC19 CAR T, or mock T cells via the tail vein on day 0. In vivo tumor growth was evaluated by luciferase live imaging (Berthold, Germany) on days 0, 7, 14, 21, 28, 35, and 42. The mice were observed for mortality up to 49 days after CAR T cell administration. Survival is graphically represented as Kaplan–Meier curves and has been analyzed using the log-rank test. Due to different luciferase-expression vectors, it is impossible to evaluate fluorescence intensity using a unified standard. Given the nature of murine hematological malignancies, tumor burden could not be directly assessed by external measurements. Therefore, humane endpoints are determined based on the level of animal discomfort. The criteria are as follows: euthanasia is carried out by carbon dioxide inhalation when the mice already manifest signs of abdominal distension due to peritoneal ascites, labored breathing, significant weight loss, impaired mobility, or paraplegia. To evaluate the CD19 targeting potential of BC19 CAR T cells, a xenograft *mouse* model based on Nalm6 cells was used. 1 × 10^6^ Nalm6 cells in 0.1 mL were injected into each female NCG mouse (GemPharmatech Co., Ltd.) on day −7. The mice were treated with 2 × 10^6^ BCMA CAR T, CD19 CAR T, BC19 CAR T, or mock T cells on day 0, respectively. In vivo tumor growth was assessed, and *mouse* mortality was monitored.

### Procedures

PBMCs were obtained from patients by leukapheresis for BC19 CAR T cells preparation on day −15 to −11. The first day of BC19 CAR T cells infusion was set on day 0. After lymphodepletion chemotherapy with cyclophosphamide (750 mg/m^2^, day −5) and fludarabine (30 mg/m^2^/day, days −5 to −2), patients received a single dose of autologous BC19 CAR T cells infusion at a dose of 1 × 10^6^ cells/kg (body weight) on day 0.

Responses were assessed on days 14 and 28 after infusion and at each follow-up session. Patients were followed monthly for the first 6 months and then every 3 months for 2 years, or until death or withdrawal from the study. The response assessment included the number of plasma cells in the bone marrow (BM), immunofixation electrophoresis, serum free light chain levels, serum paraprotein and serum immunoglobulin concentration, and 24-h urine protein analysis. Minimal residual disease (MRD) was monitored at specified times after CAR T cells infusion and defined as the absence of plasma cells (<0.01%) in BM according to EuroFlow protocol^30^. In patients with extramedullary disease, the assessment included imaging techniques (MRI, CT, or PET-CT) and physical examinations. Serum cytokine levels were measured using a Cytometric Bead Array (CBA) according to the manufacturer’s instructions. Cytogenetic and genomic aberrations were identified by karyotyping and fluorescence in situ hybridization. CAR DNA copies were assessed by quantitative real-time PCR (QPCR), as previously described. Cytokine Release Syndrome (CRS) was graded according to Lee and colleagues criteria^31^. Neurologic and other events were graded according to the Common Terminology Criteria for Adverse Events version 4.03. B-cell aplasia was defined as <1% and recovery ≥3% blood CD19^+^ cells detected by multiparameter flow cytometry (MPFC). Further details of the procedures are provided in supplementary information.

### Outcomes

The primary endpoint was safety. Safety primarily refers to the severity, frequency and duration of adverse events. The key secondary endpoint was the overall response rate (ORR). ORR was defined as the proportion of patients achieving a stringent complete response (sCR), CR, very good partial response (VGPR) or PR according to IMWG criteria at any time after infusion. Other secondary endpoints included duration of response (DOR), progression-free survival (PFS) and OS. DOR was defined as the time from the first evidence of achievement of at least PR to disease relapse or progression. PFS was defined as the period from CAR T cell infusion to disease progression or death from any cause. OS was defined as the time from infusion to death. The cutoff date was June 30, 2022.

### Statistical analysis

The statistical test used in the preclinical study is described in the corresponding figure legends. Descriptive statistics include means with standard deviations or medians with minimum and maximum for continuous variables and counts and percentages for categorical variables. Missing data were not imputed unless otherwise specified. Exact methods (Clopper-Pearson 95% confidence intervals) and Fisher’s exact test were used for categorical variables. Continuous variables were tested by Mann–Whitney U test for two groups and Kruskal–Wallis test for multiple groups. A two-tailed Student *t*-test was performed for two group comparisons. One-way ANOVA analysis of variance was used to determine the statistically significant difference for multiple group comparisons. The forest plot includes confidence intervals for individual groups and differences. DOR, PFS, and OS of patients were determined using Kaplan–Meier and compared using the log-rank test. All analyses were performed with SPSS 26 or GraphPad Prism 10. *P* values below 0.05 (two-tailed) were considered significant.

### Reporting summary

Further information on research design is available in the Nature Portfolio Reporting Summary linked to this article.

### Supplementary information

Supplementary Information

Peer Review File

Reporting Summary

### Source data

Source data

## Data Availability

Individual participants data that underlie the results reported in this article, after deidentification, will be shared upon request after publication and ending 36 months following article publication to researchers who provide a methologically sound proposal. Proposals should be directed to the corresponding author, Jiang Cao. The clinical trial study protocol is available in the Supplementary Information file. All remaining data can be found in the Article, Supplementary Information, and Source Data files. Source data are provided with this paper.
