# Supplementary material for: Bispecific CAR T cell therapy targeting BCMA and CD19 in relapsed/refractory multiple myeloma: a phase I/II trial
Source: Nat Commun. 2024 Apr 20;15:3371. doi: 10.1038/s41467-024-47801-8 (PMC11032309; doi:10.1038/s41467-024-47801-8)
Supplement: Supplementary file 1 — Supplementary Information [file 41467_2024_47801_MOESM1_ESM.pdf]

Supplementary Information

Supplementary Methods..........2
Enrollment of patients..........2
End points and Assessments..........4
MRD detection..........6
CAR T-cell expansion and persistence..........6
Supplementary Figure..........8
Supplementary Fig. 1..........8
Supplementary Fig. 2..........9
Supplementary Fig. 3..........10
Supplementary Fig. 4..........11
Supplementary Fig. 5..........12
Supplementary Fig. 6..........14
Supplementary Fig. 7..........15
Supplementary Fig. 8..........16
Supplementary Fig. 9..........17
Supplementary Tables..........18
Supplementary Table 1 Adverse events...........18
Supplementary Table 2 Adverse events of any grade by period...........19
Supplementary Table 3 Characteristics and management of CRS...........21
Supplementary Table 4 Infections..........22
Supplementary Table 5 Antibody panel for Multiple myeloma Flow-MRD..........23
Supplementary Note..........24
Supplementary Note 1. Revised International Myeloma Working Group Diagnostic Criteria for Multiple Myeloma and Smouldering Multiple Myeloma..........24
Supplementary Note 2. Staging Systems for Multiple Myeloma..........25
Supplementary Note 3. ECOG performance status..........26
Supplementary Note 4. Response Criteria for Multiple Myeloma (Revised Uniform Response Criteria by The International Myeloma Working Group)..........27
Supplementary Note 5. Response Criteria For Multiple Myeloma（Relapsed）..........29
Supplementary Note 6. CRS Grading and Management..........30
Supplementary Note 7. Study protocol..........31
Supplementary References..........86

Supplementary Methods

Enrollment of patients

Inclusion criteria

This is an open-label, single-arm, multi-center, phase I/II study, the enrolled patients were less than 70 years old and met the International Myeloma Working Group (IMWG) diagnostic criteria (see Appendix 1). Patients who were eligible for this study were confirm to be MM through histology, immunology, imaging examination and monoclonal immunoglobulin (or light chain) and had been staged according to the Durie-Salmon (DS) staging system and the International Staging System (ISS) (see Appendix 2). All patients have been treated with alkylators, proteasome inhibitors or autologous hematopoietic stem cell transplantation, and who were refractory to the last line of treatment (response less than partial response (PR) after most recent myeloma therapy or progressive disease (PD) within 60 days after most recent myeloma therapy.)

1. Male or female patients aged 5-70;

2. The patients' ECOG score is less than or equal to 2, and the estimated survival time is more than 12 weeks.

3. Patients diagnosed as refractory or recurrent plasmacytoma, including:

(1) Multiple myeloma, plasmacytic leukemia, poems syndrome, extramedullary plasmacytoma, megaglobulinemia of Fahrenheit, solitary plasmacytoma or primary amyloidosis of bone；

(2) Lymphocytic diseases containing plasma cells, such as plasmocytic lymphoma and Castleman's disease.

4. Patients with measurable or evaluable lesions.

5. The function of main tissues and organs of the patient is good:

(1) Liver function: ALT / AST < 3 times the upper limit of normal value (ULN)

(2) Renal function: creatinine < 220 μ mol / L;

(3) Pulmonary function: indoor oxygen saturation >= 95%;

(4) Cardiac function: left ventricular ejection fraction (LVEF) >= 40%.

6. Patients who are not suitable for hematopoietic stem cell transplantation or who give up transplantation or relapse after transplantation due to limited conditions
7. Patients who can take blood from vein without other contraindications of leukocyte removal.
8. Patients or their legal guardians voluntarily participate in and sign the informed consent.

Exclusion criteria

1. Pregnant or lactating women, or women who have pregnancy plans within half a year;
2. Patients with infectious diseases (such as HIV, active hepatitis B or C infection, active tuberculosis, etc.);
3. Feasibility evaluation and screening showed that the target lymphocyte was less than 10% transfected or less than 5 times amplified under the co stimulation of CD3 / CD28;
4. Patients whose vital signs are abnormal and who cannot cooperate with the examination;
5. Patients with mental or psychological diseases who can not cooperate with treatment and efficacy evaluation;
6. High allergic constitution or severe allergic history, especially for IL-2 allergy;
7. Subjects who need anti infection treatment for systemic infection or local severe infection;
8. Patients with dysfunction of heart, lung, brain, liver, kidney and other important organs;
9. Patients who cannot be included in the treatment for other reasons.

Suspension criteria

Subjects who do not complete the protocol are considered to stop the trial as soon as possible. Causes of premature termination (e.g., voluntary evacuation, toxic side effects, and deaths) must be documented in case reports. The research evaluation will be completed at the end of the suspension. Potential reasons for premature

suspension include:

1. The follow-up of this subject failed.

2. The main researchers believe that the disease is too serious to continue.

3. Patients do not comply with the treatment and clinical agreement of the study;

4. Pregnancy;

5. Voluntary withdrawal. Subjects may withdraw from research projects at any time by changing their will.

6. The significant and rapid development of malignant tumors and metastasis of central nervous system need to choose other methods, including radiotherapy or surgical treatment, but not limited to these two methods.

7. Technical difficulties encountered in the process of T cell gene modification and amplification hinder the production of clinical cell doses that meet the quality control standards.

8. In addition, if the number of patients with serious adverse events related to cell infusion exceeds 40% of the patients in the enrolled group, the researcher will suspend the study and conduct the corresponding demonstration to decide whether to continue the project.

End points and Assessments

The primary endpoint was safety. Safety mainly referred to the severity, frequency and duration of adverse events. Adverse events occurring during the first 3 months after CAR T-cell infusion were monitored continuously. After 3 months, targeted adverse events including hematological events, infections, autoimmune disorders, secondary malignancies, etc. were reported until disease progression separately. Cytokine release syndrome was graded according to criteria modified by Lee and colleagues[1]. Neurologic events and other adverse events were evaluated according to National Cancer Institute's Common Terminology Criteria for Adverse Events (V 4.03).[2] B-cell aplasia was defined as <1% and recovery ≥3% blood $CD19^+$

cells detected by multiparameter flow cytometry (MPFC). Severe adverse events that required hospitalisation, prolonged hospitalisation time, impaired work ability, endangered life, or could cause congenital malformations, must be reported as per protocol, whether it is related to the research drug or not. Clinical manifestations and vital signs associated with cytokine release syndrome, such as myalgia, fatigue, vomiting, nausea, hypoxaemia, hypotension, and neurological symptoms (e.g.,disorientation, limb tremor, disturbance of consciousness, and epilepsy), were recorded at any time during treatment. Peripheral blood was collected to detect Interleukin-6, ferritin, C-reactive protein (CRP), blood cells, coagulation profiles, creatinine, liver transaminase, and bilirubin at any time if necessary. If the patient had heart palpitations, myocardial enzymes, electrocardiogram, and troponin were measured immediately. The assessment of cytokine release syndrome was done by three experienced clinicians and was re-evaluated if inconsistent.

The key secondary endpoint was overall response rate (ORR). ORR was defined as the proportion of patients achieving a stringent complete response (sCR), CR, very good partial response (VGPR) or PR according to the IMWG criteria[3] at any time after infusion. Other secondary endpoints included duration of response (defined as the time from date of first evidence of achievement of at least PR to disease relapse or progression), progression free survival (defined as the period from CAR T cells infusion to disease progression or death from any cause), OS (defined as the time from infusion to death).

Responses were assessed on day 14 and 28 after infusion, and at each follow-up session. Patients were followed up monthly in the first 6 months and subsequently every 3 months for 2 years. All patients will be followed up until they died, lost to follow-up, or withdrew consent. The response evaluation included the number of plasma cells in the bone marrow determined by morphology, serum paraprotein, serum immunoglobulin concentration, serum free immunoglobulin light chains and quantitation of 24-h urine protein measured by-immunoturbidimetry, M-protein determined by immunofixation electrophoresis. BCMA and CD19 expression in bone marrow were assessed by flow cytometry. Minimal residual disease (MRD) was

monitored at specified times after CAR T-cell infusion according to EuroFlow protocol. In patients with extramedullary disease (including tissue masses in extraosseous locations and bone-related plasmacytomas), the assessment included imaging techniques (MRI, CT, or PET-CT), physical examination, and biopsy of involved organs for pathological examination when necessary. Patients were reassessed and given salvage therapy for disease progression or relapse at any time. The cutoff date was June 30, 2022.

MRD detection

Bone marrow was collected at different follow-up time after treatment. MRD was detected according to EuroFlow antibody panels for plasma cell disorders (PCD).

Procedure

Flow cytometric (FCM) analysis was performed on fresh specimens and isotype control antibody was used in each sample for normalization. Plasma cells were gated according to the expression of CD45 and CD38. Phenotype of plasma cells was defined as low level of CD45 and intermediate-to-high level of CD38, which was abbreviated as CD45lowCD38int/hi thereafter. Plasma cells were further analyzed for the expression of other markers such as BCMA and CD19. No less than 1.0×10^5 cells were acquired in FCM analysis for each sample.

CAR T-cell expansion and persistence

The expansion and persistence of CAR-T cells was monitored after adoptively infusion into patients. The PBMCs were harvested from patients periodically and the average copy number of CAR gene in patients was determined by quantitative real-time PCR (QPCR). The genomic DNA was purified using a Genomic DNA Purification Kit (Thermo Fisher Scientific). The plasmid containing desired fragment of CARs was used to establish standard curves.

Primers (5’-3’)

BC19 CAR forward primer: CAGGCTCCTCGCCTTCTG

BC19 CAR reverse primer: GAACCTGGCAGGGATTCCA

BC19 CAR Probe (5'-FAM, 3’-BHQ1): TCTACCACACCAGCCGGCTCCATT

Supplementary Figure

Supplementary Fig. 1

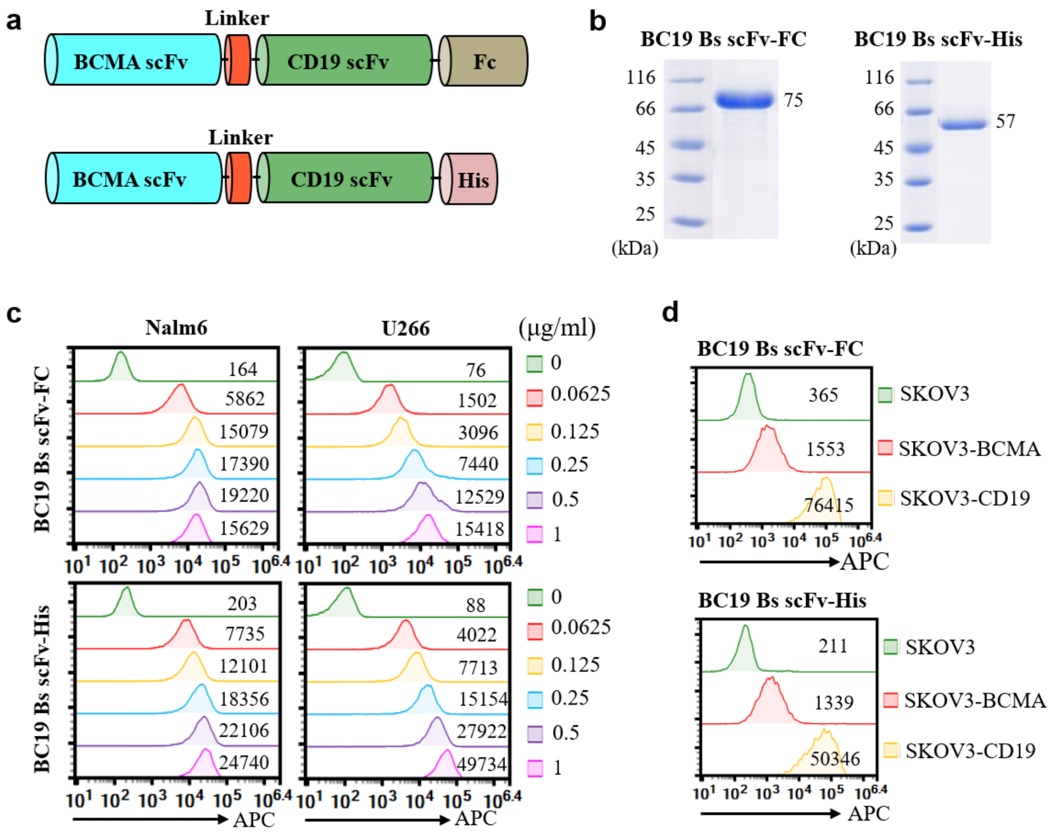

Supplementary Fig. 1 Evaluating binding specificity of the BC19 CAR binder to BCMA or CD19.

a, Schematic diagram for constructing the binder of BC19 CAR. BCMA scFv-Linker-CD19 scFv was fused with human IgG1 Fc or a His tag, respectively. b, SDS-PAGE was used to test the molecular weight and purity of the two recombinant proteins, results of 1 representative experiment are shown (n=3). c, FACS was employed to evaluate the binding of BC19 binders with Nalm6 or U266 cells, results of 1 representative experiment are shown (n=3). d, SKOV3, SKOV3-BCMA and SKOV3-CD19 cells were used to test binding specificity of the BC19 CAR binders (0.125 μg/mL), results of 1 representative experiment are shown (n=3). Source data for (b, c, d) are provided as a Source Data file.

Supplementary Fig. 2

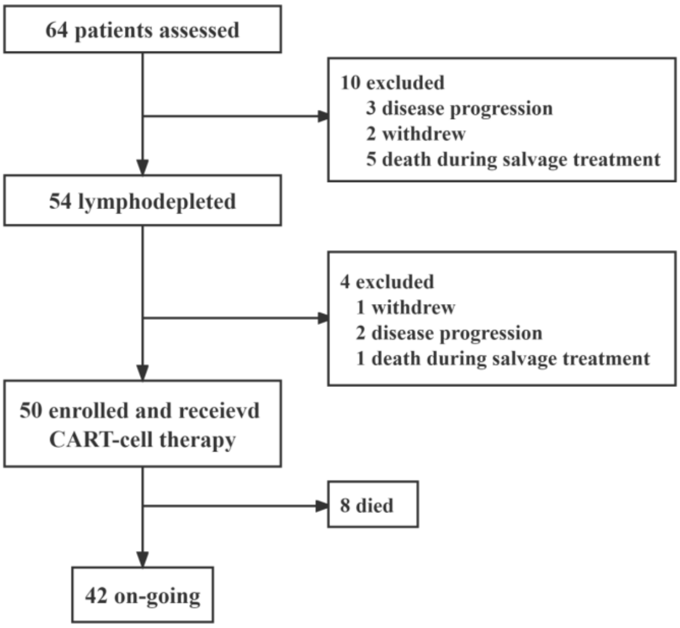

Supplementary Fig. 2 Consort Diagram.

The diagram shows all participants' courses from the time of enrolled in the study.

Supplementary Fig. 3

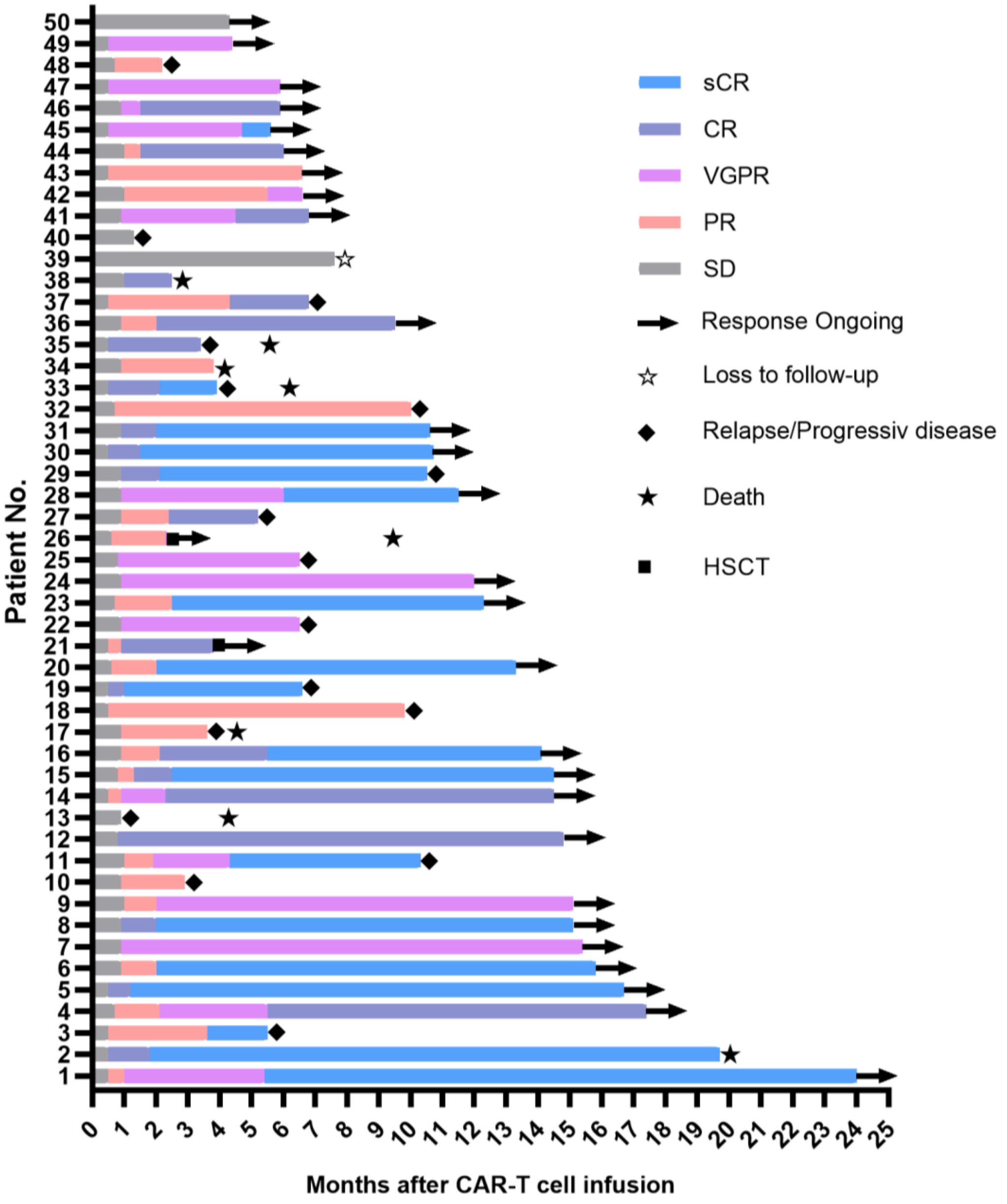

Supplementary Fig. 3 Swimmer plot for the 50 patients treated in the study.

sCR, stringent complete response; CR, complete response; VGPR, very good partial response; PR, partial response; SD, stable disease. Source data are provided as a Source Data file.

Supplementary Fig. 4

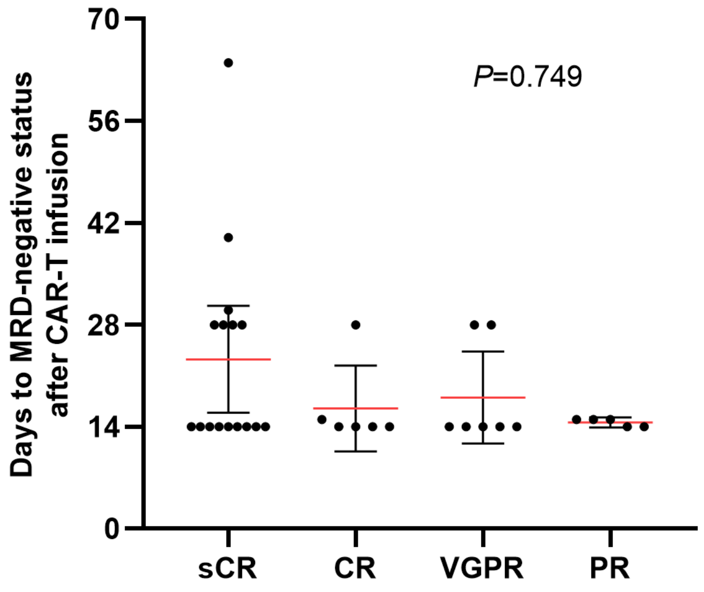

Supplementary Fig. 4 The association of the time to MRD-negativity with the depth of response.

The time to MRD-negativity in patients with stringent complete response (sCR, n=16), complete response (CR, n=6), very good partial response (VGPR, n=7) and partial response (PR, n=5) are shown. Each point represents data from 1 subject. The red line indicates the median. Two-sided P-values were determined using the Kruskal-Wallis test. CAR, chimeric antigen receptor. Raw data are provided as a Source Data file.

Supplementary Fig. 5

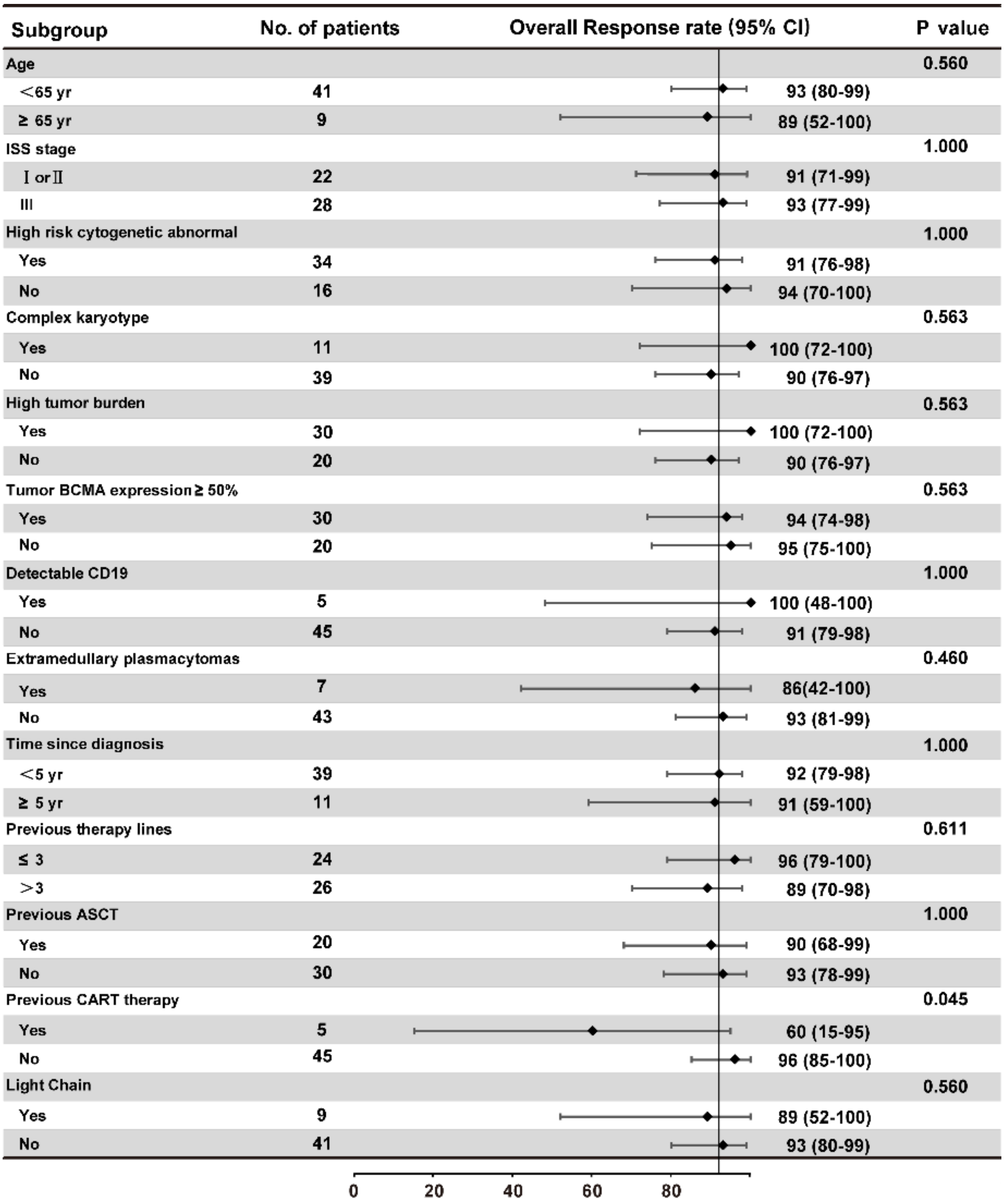

Supplementary Fig. 5 Subgroup analysis of overall response.

The panel shows the subgroup analysis of the objective response rate according to patient characteristics. Blank dots represent the observed proportions, and the lines extending from the dots are the 95% confidence intervals (CI) for these proportions. The 95% CI was calculated with the Clopper-Pearson method. High-risk cytogenetic

profile was defined by the presence of the following abnormalities: 1q21, deletion 17p, deletion 13q, t(4;14), t(11; 14) and t(14; 16). High tumor burden was defined as at least 50% clonal plasma cells or bone marrow plasma cells. Extramedullary disease was defined as soft-tissue masses spreading outside the bone marrow. ASCT, autologous stem-cell transplantation. BCMA, B-cell maturation antigen; CR, complete response; ISS, International Staging System. Source data are provided as a Source Data file.

Supplementary Fig. 6

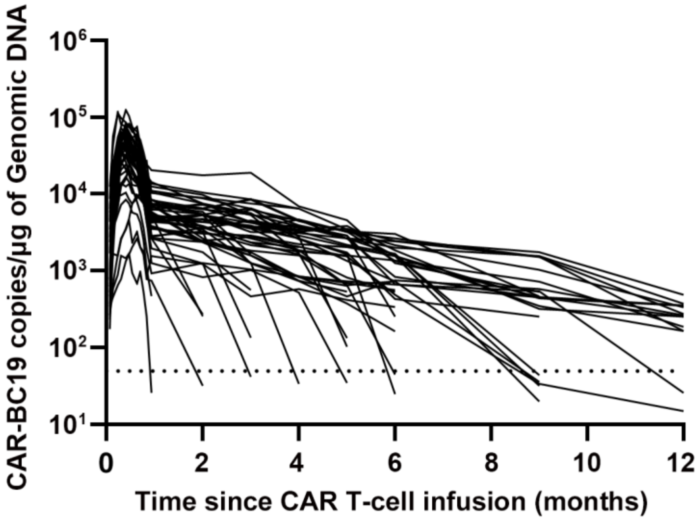

Supplementary Fig. 6 CAR T-cell expansion and persistence.

The copies of CAR-BC19 transgenes are shown respectively in 50 patients with assessable CAR-transgenes. Genomic DNA was isolated from the samples of whole blood at serial time points before and after infusion of CAR T-cells within the first 12 months. The horizontal line in each panel denotes the lower limit of quantitation (50 copies/μg). Source data are provided as a Source Data file.

Supplementary Fig. 7

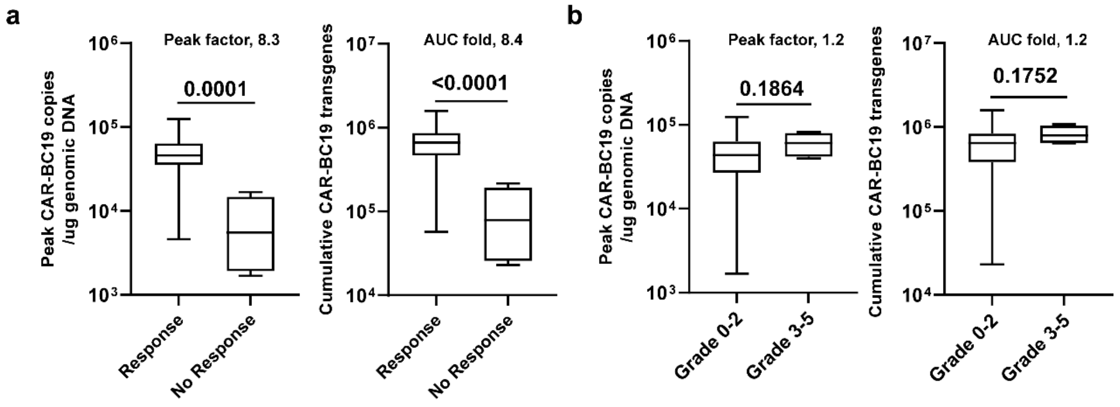

Supplementary Fig. 7 The association of CAR T-cell expansion with response and cytokine release syndrome (CRS).

Panel a show the association of BC19 CAR T-cell expansion with overall response. Panel b shows the association of BC19 CAR T-cell expansion with CRS. CAR Area Under the Curve (AUC) is defined as cumulative levels of CAR transgene of blood over the first 28 days (Response, n=46, No response, n=4). The peak factor and AUC fold change are shown for patients with *vs.* without response or grade ≥3 *vs.* grade 0-2 CRS. The horizontal line within each box represents the median, and the lower and upper borders of each box represent the 25th and the 75th percentiles, respectively, and the bars represent the minimum and maximum range (Grade 0-2, n=46, No Grade 3-5, n=4). *P* values were calculated by Wilcoxon rank sum test. Source data are provided as a Source Data file.

Supplementary Fig. 8

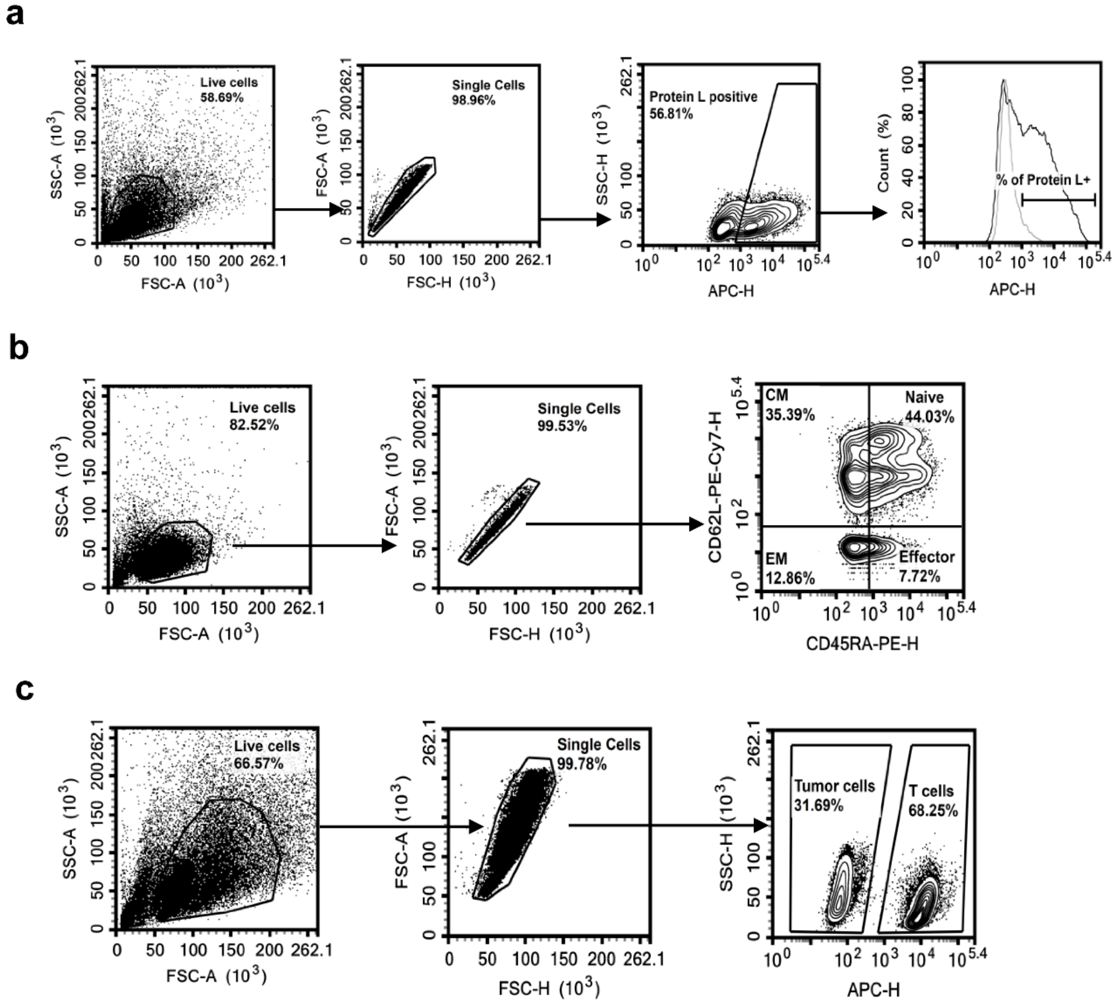

Supplementary Fig. 8 Gating strategy for Figure 1.

a. Gating strategy for quantifying the transduction efficiency of CAR T cell production, corresponding to Figure 1b of the main text. T cells labeled with Biotinylated Protein L followed by APC-conjugated Streptavidin were identified as CAR T cells. b. Gating strategy for memory phenotype analysis of CAR T cell production using PE-anti human CD45RA antibody and PE-Cy7-anti human CD62L antibody, corresponding to Figure 1c of the main text. c. Gating strategy for detection of CAR T cell-mediated killing of U266 or Nalm6 cells, corresponding to Figure 1e of the main text. CAR T cells were stained with CellTracker Deep Red (CTDR) before coinciding with tumor cells.

Supplementary Fig. 9

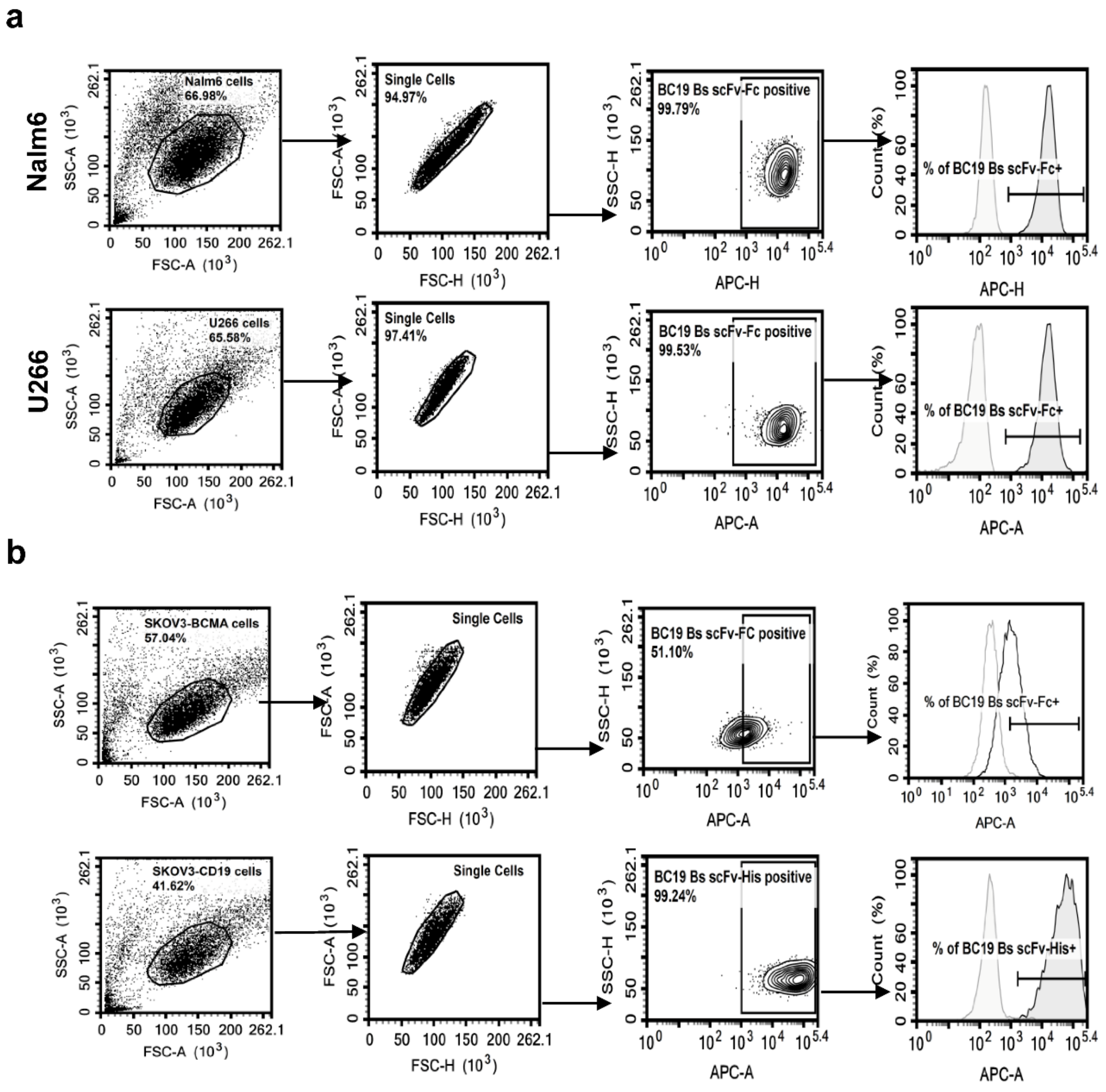

Supplementary Fig. 9 Gating strategy for Supplementary Figure 1.

a. Gating strategy for assessing the binding ability of BC19 CAR binders to CD19 or BCMA, corresponding to Supplementary Figure 1c of the main text. Nalm6 and U266 tumor cells labeled with Biotinylated BC19 binders followed by APC-conjugated Streptavidin were identified as $CD19^+$ or $BCMA^+$ cells. b. The gating strategy was used to test the binding specificity of BC19 CAR binders, corresponding to Supplementary Figure 1d of the main text. SKOV3, SKOV3-BCMA and SKOV3-CD19 cells were labeled with Biotinylated BC19 binders followed by APC-conjugated Streptavidin.

Supplementary Tables

Supplementary Table 1 Adverse events.

Any adverse event	Any Grade	Grade 3-5
	number of patients (percent)	
Any	50 (100)	50 (100)
Haematological		
Neutropenia	50 (100)	49 (98)
Anemia	47 (94)	32 (64)
Thrombocytopenia	44 (88)	33 (66)
Leukopenia	50 (100)	48 (96)
Pulmonary		
Pleural effusion	4 (8)	1 (2)
Gastrointestinal		
Diarrhea	4 (8)	0
Nausea	15 (30)	0
Constipation	1 (2)	0
Decreased appetite	12 (24)	0
Hepatic		
AST increased	27 (54)	12 (24)
ALT increase	23 (46)	4 (8)
Nephritic		
Creatinine increase	8 (16)	0
Others		
Hypokalemia	33 (66)	8 (16)
Hypocalcemia	39 (78)	5 (10)
Hyponatremia	23 (46)	2 (4)
Hypophosphatemia	26 (52)	0
Hypomagnesemia	10 (20)	0
Hypoalbuminemia	33 (68)	1 (2)
Headache	6 (12)	0
Cough	12 (24)	0
Fatigue	14 (28)	0
Pyrexia	47 (94)	12 (24)
Hypotension	8 (16)	0
Hypogammaglobulinemia	23 (46)	0
B-cell aplasia	45 (90)	0
Cytokine release syndrome†	46 (92)	4 (8)
Neurotoxic effect	2 (4)	0

†Events were uniformly graded according to Lee et al[1].

Supplementary Table 2 Adverse events of any grade by period.

Adverse events	Patients with early events≤3 months after infusion (n=50)		Patients with later events > 3 months after infusion (n=49) #	
	Any Grade	Grade 3-5	Any Grade	Grade 3-5
	number of patients (percent)			
Hematologic				
Neutropenia	50 (100)	49 (98)	26 (53)	9 (18)
Anemia	47 (94)	32 (64)	26 (53)	6 (12)
Thrombocytopenia	44 (88)	33 (66)	17 (35)	11 (22)
Leukopenia	50 (100)	48 (96)	23 (47)	6 (12)
Immue system disorder				
Hypogammaglobulinemia	23 (46)	0	23 (47)	-
Gastrointestinal				
Diarrhea	4 (8)	0	7 (14)	0
Nausea	15 (30)	0	1 (2)	0
Hepatic				
AST increased	27 (54)	12 (24)	3 (6)	0
ALT increased	23 (46)	4 (8)	3 (6)	0
Infection※				
Any infection	30 (60)	7 (14)	19 (37)	10 (20)
Lung infection	23 (46)	6 (12)	17 (35)	9 (19)
Urinary tract infection	2 (4)	0	0	0
Batcteremia	0	0	1 (2)	1 (2)
Herpes zoster infection	0	0	1 (2)	0
Other				
Hypokalemia	33 (66)	8 (16)	6 (12)	0
Hypophosphatemia	26 (52)	0	3 (6)	0
Hypocalcemia	39 (78)	5 (10)	4 (8)	0
Hypomagnesemia	10 (20)	0	1 (2)	0
Hyponatremia	23 (46)	2 (4)	5 (10)	0
Hypoalbuminemia	33 (68)	1 (2)	5 (10)	0
Fatigue	14 (28)	0	4 (8)	0
Headache	6 (12)	0	1 (2)	0
Pyrexia	47 (94)	12 (24)	8 (16)	0
Decreased appetite	12 (24)	0	4 (8)	1 (2)
Cough	12 (24)	0	10 (20)	0
Sinus tachycardia	1 (2)	0	0	0
Arthralgia	1 (2)	0	1 (2)	0
Pain in extremity	2 (4)	0	3 (6)	0
Hypotension	8 (16)	0	0	0
B-cell aplasia	45 (90)	0	40 (82)	0

CRS¶	46 (92)	4 (8)	0	0
Neurologic events	2 (4)	0	0	0
CRS intervention				
Tocilizumab	19 (38)	3 (6)	-	-
Glucocorticoids	13 (26)	3 (6)	-	-

Late adverse events including hematological and immunologic events, and infections from patients were reported until multiple myeloma progression. For reporting the hematological and immunologic events, 40 patients with ongoing response for more than 3 months were analyzed.

※ The total of number and percent of patients with different types of infections were not equal to those of patients with any infection due to repeated infections in some patients.

¶ CRS was graded according to criteria proposed by Lee et al[1]. Individual symptoms of the CRS and neurologic events were graded according to the National Cancer Institute Common Terminology Criteria for Adverse Events, version 4.03.

Supplementary Table 3 Characteristics and management of CRS.

Parameter	Grade 1	Grade 2	Grade 3
Patients with CRS* event-no. (%)	34 (68)	8 (16)	4 (8)
Median (range) time to onset-days	7.5 (1-24)	6 (1-12)	8 (6-10)
Median (range) duration-days	2.5 (1-11)	4 (1-21)	4 (3-5)
Tocilizumab use-no. (%)†	11 (22)	5 (10)	3 (6)
Glucocorticoid use-no. (%)	8 (16)	5 (10)	3 (6)

CRS denotes cytokine release syndrome.

*Uniformly graded per Lee DW, et al[1].

†The decision to give tocilizumab was at the treating physician's discretion based on protocol-specified toxicity management guidelines.

Supplementary Table 4 Infections

Infections	Patients with early events ≤ 3 months (n=50)		Patients with late events >3 months (n=49)	
	Any Grade	Grade 3-5	Any Grade	Grade 3-5
	Number of patients (percent)			
Pathogen unspecified	22 (44)	2 (4)	14 (29)	4 (8)
Viral	1 (2)	0	1 (2)	0
Bacterial	9 (18)	3 (6)	5 (10)	1 (2)
Fungal	3 (6)	3 (6)	0	0

Infections were graded according to the National Cancer Institute Common Terminology Criteria for Adverse Events, version 4.03

Supplementary Table 5 Antibody panel for Multiple myeloma Flow-MRD

Fluorescent dyes	Tube 1	Tube 2
Qdot 565	CD45	CD45
APC-CY7	CD38	CD38
APC	CD138	CD138
PerCP-CY5.5	CD19	CD19
PE-CY7	BCMA	BCMA
BV510	CD27	
V450	CD56	
FITC		CyIgκ
PE		CyIgλ

Supplementary Note

Supplementary Note 1. Revised International Myeloma Working Group Diagnostic Criteria for Multiple Myeloma and Smouldering Multiple Myeloma

Definition of multiple myeloma

Clonal bone marrow plasma cells ≥10% or biopsy-proven bony or extramedullary plasmacytoma and any one or more of the following myeloma defining events:

Myeloma defining events:

Evidence of end organ damage that can be attributed to the underlying plasma cell proliferative disorder, specifically:

- Hypercalcaemia: serum calcium >0.25 mmol/L (>1 mg/dL) higher than the upper limit of normal or >2.75 mmol/L (>11 mg/dL)
- Renal insufficiency: creatinine clearance <40 mL per min† or serum creatinine>177 µmol/L (>2 mg/dL)
- Anemia: hemoglobin value of >20 g/L below the lower limit of normal, or a hemoglobin value <100 g/L
- Bone lesions: one or more osteolytic lesions on skeletal radiography, CT, or PET-CT

Any one or more of the following biomarkers of malignancy:

- Clonal bone marrow plasma cell percentage ≥60%
- Involved: uninvolved serum free light chain ratio ≥100
- >1 focal lesion on MRI studies

Definition of smouldering multiple myeloma

Both criteria must be met:

- Serum monoclonal protein (IgG or IgA) ≥30 g/L or urinary monoclonal protein ≥500 mg per 24 h and/or clonally bone marrow plasma cells 10–60%
- Absence of myeloma defining events or amyloidosis

Supplementary Note 2. Staging Systems for Multiple Myeloma

Stage	Durie-Salmon Criteria
I	All of the following:<br>• Hemoglobin value >10 g/dL<br>• Serum calcium value normal or ≤12 mg/dL<br>• Bone x-ray, normal bone structure) or solitary bone plasmacytoma only<br>• Low M-component production rate:<br>IgG value <5 g/dl;<br>IgA value <3 g/dl<br>Bence Jones protein <4 g/24 h
II	Neither stage l nor stage lll
III	One or more of the following:<br>• Hemoglobin value <8.5 g/dl<br>• Serum calcium value >12 mg/dl<br>• Advanced lytic bone lesions<br>• High M-component production rate:<br>IgG value >7 g/dl;<br>IgA value >5 g/dl<br>Bence Jones protein >12 g/24 h
Subclassifiation Criteria<br>A Normal renal function (serum creatinine level <2.0 mg/dl)<br>B Abnormal renal function (serum creatinine level ≥2.0 mg/dl)	

Stage	International Staging System
I	Serum β2-microglobulin <3.5 mg/L and serum albumin ≥ 3.5 g/dL
II	Serum β2-microglobulin < 3.5 mg/L and serum albumin < 3.5 g/dL or serum β2-microglobulin 3.5 to < 5.5 mg/L
III	Serum β2-microglobulin ≥ 5.5 mg/L

Supplementary Note 3. ECOG performance status

Grade	ECOG performance status
0	Fully active, able to carry on all pre-disease performance without restriction
1	Restricted in physically strenuous activity but ambulatory and able to carry out work of a light or sedentary nature, e.g., light house work, office work
2	Ambulatory and capable of all self-care but unable to carry out any work activities; up and about more than 50% of waking hours
3	Capable of only limited self-care; confined to bed or chair more than 50% of waking hours
4	Completely disabled; cannot carry on any self-care; totally confined to bed or chair
4	Dead

Supplementary Note 4. Response Criteria for Multiple Myeloma (Revised Uniform Response Criteria by The International Myeloma Working Group)

Response Category	Response Criteria
sCR	CR as defined plus normal FLC ratio and absence of clonal plasma cells by immunohistochemistry or two- to four-color flow cytometry; two consecutive assessments of laboratory parameters are needed
CR	Negative immunofixation of serum and urine, disappearance of any soft tissue plasmacytomas, and <5% plasma cells in bone marrow; in patients for whom only measurable disease is by serum FLC level, normal FLC ratio of 0.26 to 1.65 in addition to CR criteria is required; two consecutive assessments are needed
VGPR	Serum and urine M component detectable by immunofixation but not on electrophoresis or ≥90% reduction in serum M component plus urine M component <100 mg/24 h; in patients for whom only measurable disease is by serum FLC level, >90% decrease indifference between involved and uninvolved FLC levels, in addition to VGPR criteria, is required; two consecutive assessments are needed
PR	≥50% reduction of serum M-protein and reduction in 24-hour urinary M-protein by ≥90% or to <200 mg per 24 hIf the serum and urine M-protein are unmeasurable, a ≥50% decrease in the difference between involved and uninvolved FLC levelsis required in place of the M-protein criteria. If serum and urine M-protein are unmeasurable, and serum free light assay is also unmeasurable, ≥50% reduction in plasma cells is required in place of M-protein, provided baseline bone marrow plasma cell percentage was ≥30%. In addition, if present at baseline, a ≥50% reduction in the size of soft tissue plasmacytomas is also required.<br>Two consecutive assessments are needed; no known evidence of progressive or new bone lesions if radiographic studies were performed.
MR	≥25% but ≤49% reduction of serum M protein and reduction in 24-hour urine M protein by 50% to 89%<br>In addition, if present at baseline, 25% to 49% reduction in size of soft tissue plasmacytomas is also required<br>No increase in size or number of lytic bone lesions (development of compression fracture does not exclude response)
SD	Not meeting criteria for CR, VGPR, PR or progressive disease; no known

	evidence of progressive or new bone lesions if radiographic studies were performed
PD, progressive disease	Increase of 25% from lowest response value in any of following:<br>Serum M component with absolute increase ≥0.5 g/dL; serum M component increases ≥1 g/dL are sufficient to defined relapse if starting M component is ≥5 g/dl and/or;<br>Urine M component (absolute increase must be ≥200 mg/24 h) and/or;<br>Only in patients without measurable serum and urine M protein levels: difference between involved and uninvolved FLC levels (absolute increase must be >10 mg/dl);<br>Only in patients without measurable serum and urine M protein levels and without measurable disease by FLC level, bone marrow plasma cell percentage (absolute percentage must be ≥10%)<br>Development of new or definite increase in size of existing bone lesions or soft tissue plasmas<br>Development of hypercalcemia that can be attributed solely to plasma cell proliferative disorder<br>Two consecutive assessments before new therapy are needed

Supplementary Note 5. Response Criteria For Multiple Myeloma（Relapsed）

Relapse Subcategory	Relapse Criteria
Clinical relapse	Clinical relapse requires one or more of:<br>•Direct indicators of increasing disease and/or end organ dysfunction (CRAB features). It is not used in calculation of time to progression or progression-free survival but is listed here as something that can be reported optionally or for use in clinical practice<br>• Development of new soft tissue plasmacytomas or bone lesions<br>• Definite increase in the size of existing plasmacytomas or bone lesions. A definite increase is defined as a 50% (and at least 1 cm) increase as measured serially by the sum of the products of the cross-diameters of the measurable lesion<br>• Hypercalcemia (>11.5 mg/dL) [2.65 mmol/L]<br>• Decrease in hemoglobin of ≥2 g/dL [1.25 mmol/L]<br>• Rise in serum creatinine by 2 mg/dL or more [177 μmol/L or more]
Relapse from CR	Any one or more of the following:<br>• Reappearance of serum or urine M-protein by immunofixation or electrophoresis<br>• Development of ≥5% plasma cells in the bone marrow<br>• Appearance of any other sign of progression (ie, new plasmacytoma, lytic bone lesion, or hypercalcemia)

Supplementary Note 6. CRS Grading and Management

Grade	Criteria
1	Mild reaction:<br>Treated with supportive care such as antipyretics, antiemetics.
2	Moderate reaction<br>requiring IV therapies or parenteral nutrition; some signs organ dysfunction (i.e. grade 2 creatinine or grade 3 liver function tests) related to CRS and not attributable to any other condition. Hospitalization for management of CRS related symptoms including fevers associated neutropenia
3	More severe reaction:<br>Hospitalization required for management of symptoms related to organ dysfunction including grade 4 LFTs or grade 3 creatinine related to CRS and not attributable to any other conditions; this excludes management of fevers or myalgias. Includes hypotension treated with IVFs or low-dose pressors, coagulopathy requiring fresh frozen plasma (FFP) or cryoprecipitate, and hypoxia requiring supplemental oxygen (nasal cannula oxygen, high flow oxygen, Continuous Positive Airway Pressure [CPAP] or Bilateral Positive Airway Pressure [BiPAP]). Patients admitted for management of suspected infection due to fevers and/or neutropenia may have grade 2 CRS.
4	Life-threatening complications such as hypotension requiring pressors, hypoxia requiring mechanical ventilation.
5	Death

Supplementary Note 7. Study protocol

Clinical study for anti-BCMA and anti-CD19 double targets CAR-T in the treatment of refractory or recurrent plasma cell tumor

PI/Department: Jiang Cao, M.D. & Ph.D./Department of hematology

Funding Sponsor:

Study sponsor and monitor: The Affiliated Hospital of Xuzhou Medical University

Study duration: 2020.06-2023.06

Version: V1.0

Protocol updated: 2020.05.1

1. Abstract

**Title**	Clinical study for anti-BCMA and anti-CD19 double targets CAR-T in the treatment of refractory or recurrent plasma cell tumor
**Phase**	Phase I/II
**Study Design**	This is an open-label and single-arm study to assess the safety and efficacy of anti-BCMA and anti-CD19 double targets CAR-T cells for relapsed and refractory multiple myeloma
**Number of subjects**	Approximately 60 subjects will be enrolled into this study.
**Summary of Subject Eligibility Criteria**	<u>Inclusion criteria:</u><br>1. Male or female patients aged 5-70;<br>2. The patients' ECOG score is less than or equal to 2, and the estimated survival time is more than 12 weeks;<br>3. Patients diagnosed as refractory or recurrent plasmacytoma, including<br>(1) Multiple myeloma, plasmacytic leukemia, poems syndrome, megaglobulinemia of Fahrenheit, extramedullary plasmacytoma, solitary plasmacytoma or primary amyloidosis of bone;<br>(2) Lymphocytic diseases containing plasma cells, such as plasmocytic lymphoma and Castleman's disease.<br>4. Patients with measurable or evaluable lesions;<br>5. The function of main tissues and organs of the patient is good:<br>(1) Liver function: ALT / AST < 3 times the upper limit of normal value (ULN);<br>(2) Renal function: creatinine < 220 μ mol / L;<br>(3) Pulmonary function: indoor oxygen saturation >= 95%;<br>(4) Cardiac function: left ventricular ejection fraction (LVEF) >= 40%.<br>6. Patients who are not suitable for hematopoietic stem cell transplantation or who give up transplantation or relapse after transplantation due to limited conditions;<br>7. Patients who can take blood from vein without other contraindications of leukocyte removal;<br>8. Patients or their legal guardians voluntarily participate in and sign the informed

	consent.
	Exclusion Criteria:<br>1. Pregnant or lactating women, or women who have pregnancy plans within half a year;<br>2. Patients with infectious diseases (such as HIV, active hepatitis B or C infection, active tuberculosis, etc.);<br>3. Feasibility evaluation and screening showed that the target lymphocyte was less than 10% transfected or less than 5 times amplified under the co stimulation of CD3 / CD28;<br>4. Patients whose vital signs are abnormal and who cannot cooperate with the examination;<br>5. Patients with mental or psychological diseases who can not cooperate with treatment and efficacy evaluation;<br>6. High allergic constitution or severe allergic history, especially for IL-2 allergy;<br>7. Subjects who need anti infection treatment for systemic infection or local severe infection;<br>8. Patients with dysfunction of heart, lung, brain, liver, kidney and other important organs;<br>9. Patients who cannot be included in the treatment for other reasons.
**Study Product, Dose, Route**	Anti-BCMA and anti-CD19 double targets CAR-T cells, single dose intravenous infusion (1×10^6 cells/kg body weight)
**Objectives**	Primary outcome measures: The primary endpoint was safety. Safety mainly referred to the severity, frequency and duration of adverse events, including hematologic toxicity, CRS, neurotoxic events, and other related adverse events. Adverse events occurring during the first 3 months after CAR T-cell infusion were monitored continuously. After 3 months, targeted adverse events including hematological events, infections, autoimmune disorders, secondary malignancies, etc. were reported until disease progression separately.

	Secondary outcome measures: The key secondary endpoint was overall response rate (ORR). ORR was defined as the proportion of patients achieving a stringent complete response (sCR), CR, very good partial response (VGPR) or PR according to the IMWG criteria3 at any time after infusion. Other secondary endpoints included duration of response (defined as the time from date of first evidence of achievement of at least PR to disease relapse or progression), progression free survival (defined as the period from CAR T cells infusion to disease progression or death from any cause), OS (defined as the time from infusion to death).
**Statistical Methodology**	Descriptive statistics includes medians with minimum and maximum for continuous variables, and counts and percentages for categorical variables. The continuous variables are tested by Mann-Whitney U test for two groups and Kruskal-Wallis test for multiple groups. All adverse events will be described. Kaplan-Meier curves are used for analyzing duration of response, progression-free survival and overall survival and were compared with the use of log-rank test. Subgroups or covariate analyses of efficacy and / or safety will be needed.
**Study duration**	The amount of time required to complete this trial will depend on the number of patients. We estimate it will take approximately 4 years to complete this trial.

2. Background

Multiple myeloma (MM) is a disease defined by the accumulation of clonal bone marrow plasma cells and development of clinical complications including hypercalcemia, renal insufficiency, symptomatic anemia, destructive lytic bone lesions, and susceptibility to infections. In the past 30 years, the treatment of MM has been developed. From early cyclophosphamide and melphalan to combined chemotherapy in the 1980s, the efficacy of MM treatment has been significantly improved. In recent years, hematopoietic stem cell transplantation (HSCT)[1-2], protease inhibitor[3], immunomodulator[4], daratumab[5-6] and other applications[7-8] have further improved the prognosis of MM patients. However, MM is still an incurable disease, especially the prognosis of relapsed and refractory (R/R) patients is still poor[9]. It is necessary to explore more effective treatment methods to improve the prognosis and survival time of R/R MM patients.

In recent years, immunotherapy has become a focus in cancer therapy[10-14.] Genetically engineered T cells carrying specific antigen binding sequences can specifically recognize and kill tumor cells in a non-MHC dependent manner[14-16]. T cells modified with chimeric antigen receptors (CARs) specific to MM cell antigen have also been developed and achieved promising response, which have become a new treatment approach for R/R MM[17-20].

CD19 is the surface marker of B lymphocyte. Almost all B lymphocytic tumors express CD19. Several reports have identified in MM patients a minor component of the multiple myeloma expresses CD19 that is related to drug-resistant, disease-propagating properties[17,21]. The treatment of R/R MM with anti-CD19 CAR-T cells (CTL019) has entered Phase I clinical trial (NCT02135406, Pennsylvania, USA). After treated with CTL019 cells followed by melphalan and autologous hematopoietic stem cell transplantation (ASCT), the concentration of monoclonal immunoglobulin (M protein), IL-6 and the expression of immunoglobulin heavy chain gene decreased. 6 of 10 patients in the clinical trial maintained complete remission (CR). The main adverse reactions of CTL019 were grade 1 cytokine release syndrome (CRS) and ASCT-induced enterocolitis[17].

B cell maturation antigen (BCMA) is mainly expressed by mature B cells, normal plasma cells, malignant plasma cells and plasmacytoid dendritic cells, but not by primary B cells, memory B cells, normal hematopoietic stem cells and other non-hematopoietic cells[19, 22]. BCMA belongs to the TNF receptor superfamily, which combines B cell activating factor (BAFF) and a proliferation inducing ligand (APRIL) to promote the growth of MM cells and the adhesion of bone marrow stromal

cells[23]. BCMA antibody can kill both MM cell lines and primary MM cells[24]. The number of B cells in BCMA deficient mice is normal, but the function of B cells is inhibited[25]. These data suggest that BCMA is suitable as a target for MM therapy and does not significantly affect the function of normal B cells. The scFv of BCMA-CAR-T cells containing C12A3.2 or C111D5.3 target BCMA-1 and BCMA-2, respectively and CAR-T cells showed obviously reactive proliferation and killing activity when co-cultured with BCMA-positive MM cells[19]. Anti-BCMA CAR-T cells can recognize and kill MM cells of patients, and play an anti-tumor role through perforin pathway in MM murine model. In December 2016, Bluebird Company published the phase I clinical data of anti-BCMA CAR-T in the treatment of R/R MM. Nine patients were enrolled in the study and received three different doses of CAR-T cells, e.g. 5.0×10^7 (dose 1), 15.0×10^7 (dose 2) and 45.0×10^7 (dose 3). The results showed that the overall response rate (ORR) was 78% and 100% in the dose 2 and dose 3 groups, respectively. Two patients met CR criteria at 4- and 6-months follow-up. In dose 2 and dose 3 groups, MM cells were undetected in the bone marrow beyond 14 days after CAR-T cell infusion. In dose 1 group, ORR was 33%. At present, many research institutes around the world are carrying out research on BCMA CAR-T, CD19 CAR-T and other CAR-T cells for the treatment of MM. Preliminary data show that CAR-T can effectively treat R/R MM patients. We previously conducted a prospective study and demonstrated that combined infusion of humanised anti-CD19 and anti-BCMA CAR T cells is feasible in patients with R/R MM[26]. On the basis of these preliminary data, we designed a second-generation bispecific BC19 CAR containing an anti-BCMA single-chain variable fragment (scFv) and a humanized anti-CD19 scFv. This study is to determine the safety and efficacy of anti-BCMA and anti-CD19 double targets CAR-T cells in the treatment of R/R MM patients. This study may provide data for the new treatment strategy of R/R MM.

3. Previous researches

3.1 Construction of Lentivirus Expression Vector for Chimeric Antigen Receptor

The bispecific BCMA/CD19 (BC19)- and CD19/BCMA (19BC)-CARs containing anti-BCMA single chain variable fragment (scFv) and anti-CD19 scFv in tandem, linked by GGGGS×4, were constructed in this study, respectively. The lentiviral transfer plasmid contains an anti-CD19, anti-BCMA, anti-BC19 or anti-19BC domain, human CD8α hinge and transmembrane region and human 4-1BB and

human CD3ζ signaling moieties. The lentivirus was manufactured by Genechem Co., LTD (Shanghai, China).

3.2 Manufacture of anti-BCMA and anti-CD19 double targets CAR-T-cells

Primary peripheral blood mononuclear cells (PBMCs) were isolated from peripheral blood of the healthy donor and the patients with R/R MM. T lymphocytes were isolated using EasySepTM human T Cell Isolation Kit (STEMCELL) according to the manufacture's instruction. The production procedure of CAR-T cells has been described in our previous studies[26].

3.3 CAR T cell proliferation assay and phenotype identification

1×10^7 human T cells were infected by the lentivirus mentioned above. To draw cellular growth curves, the live T cells were counted by using trypan blue staining and Automated Cell Counter (Thermo Fisher, CountessTM 3). CD19-, BCMA-, BC19- or 19BC-CAR expression was evaluated by PE-Labeled Protein L (Acro Biosystems).

FITC-labeled anti-CD45RA (BD Biosciences) and PECy7-labeled anti-CD62L (BD Biosciences) were employed to determine phenotypes of infusion products of CAR-T, including naïve ($CD45RA^+$ $CD62L^+$), central memory ($CD45RA^-$ $CD62L^+$), effector-memory ($CD45RA^-$ $CD62L^-$) and effector ($CD45RA^+$ $CD62L^-$) T cells.

3.4 CAR T cell killing assay

For evaluating the in vitro cytotoxicity of CAR T cells, Nalm-6 or U266 cells were labeled with CellTracker Deep Red (CTDR, 0.25 μM) for 25 min at 37°C and then cocultured with the CAR T cells labeled with carboxyfluorescein diacetate succinimidyl ester (CFSE) for 24 hours. After washing with phosphate buffered saline (PBS) twice, the labeled cells were analyzed on a FACScan flow cytometer using CellQuest software (BD Biosciences). % CTDR+ cells represent the CAR T Cell mediated cytotoxicity. To further evaluate specific cytotoxicity of the CAR T cells, Real Time Cellular Analysis (RTCA) was employed. SKOV3, SKOV3-CD19 and SKOV3-BCMA cells were used as target cells, being added into each well of 96-well E-Plate (Agilent Technologies). After proliferation was monitored for 23 hours, either mock or the CAR T cells were added. T-cell numbers were varied to achieve E:T ratios of 3:1 or 1:3. The data were collected by using xCELLigence (Agilent, RTCA DP).

3.5 Enzyme-linked immunosorbent assay (ELISA)

The level of interferon γ (IFN-γ) in the supernatants of the co-culture system was detected using the ELISA kits (R&D Systems) following the manufacturer's instructions.

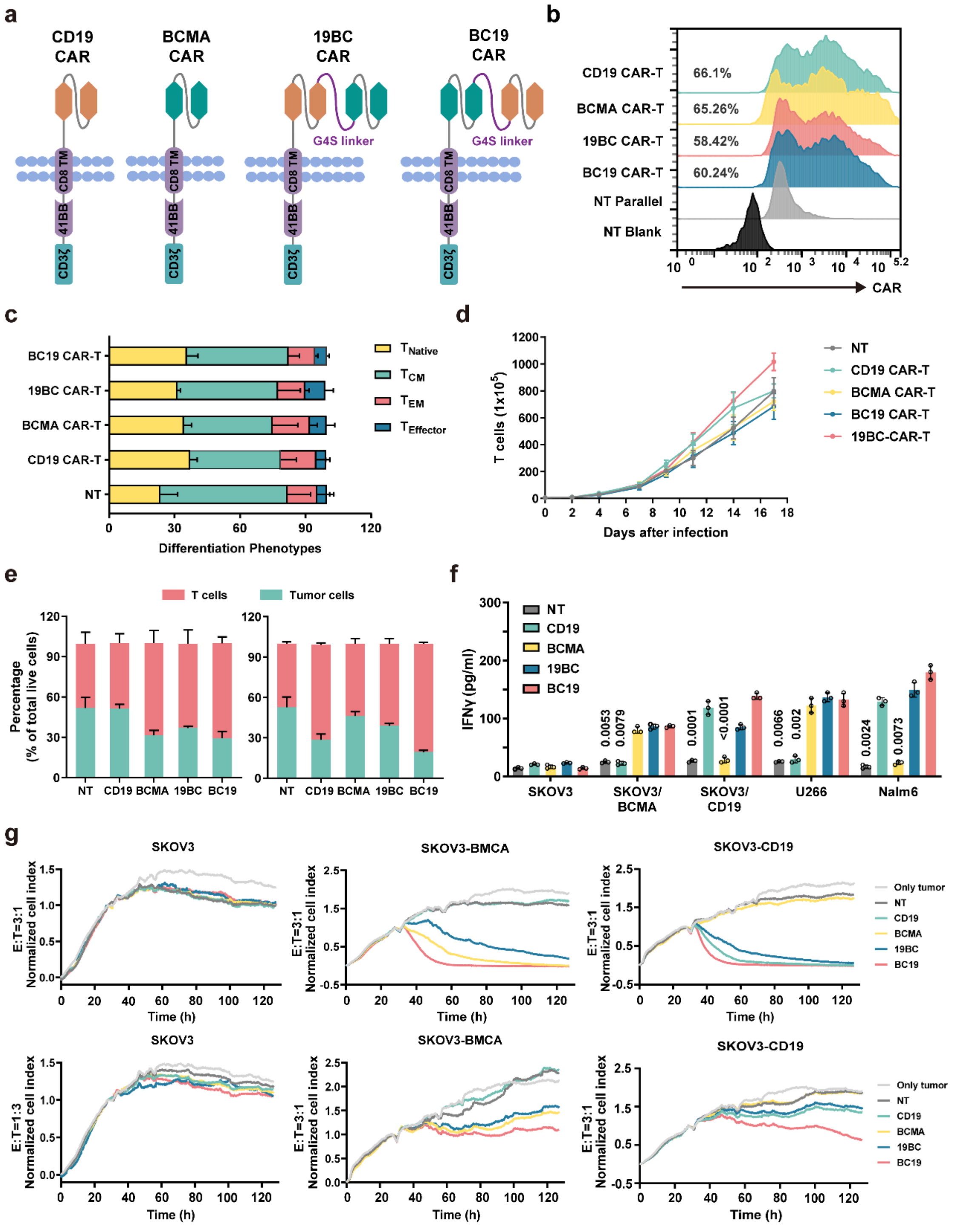

Fig. 1 Construction and functional validation of mono- or bi-specific CAR T cells targeting BCMA or/and CD19 in vitro.

3.6 In vivo xenograft mouse model

For establishing MM xenograft mouse models, six- to eight-weeks old male NCG (NOD/ShiLtJGpt-Prkdcem26Cd52Il2rgem26Cd22/Gpt) mice were purchased from GemPharmatech Co., Ltd. A total of 0.1 mL of the U266 cell suspension (2×10^7 cells/mL) was injected via the tail vein

on day -7. In order to maintain BCMA expression on the surface of U266 cells in vivo, γ-secretase inhibitor LY3039478 was employed23. LY3039478 was formulated in 1% carboxymethylcellulose and 0.25% Tween 80, suspended by probe and water bath sonication, and then orally administrated into the mice at 1 mg/kg three times per week. Mice were randomly divided into 4 groups (n=3 or 4) and injected with a single dose of 2×10^6 BCMA CAR T, CD19 CAR T, BC19 CAR T or mock T cells via the tail vein on day 0. In vivo tumor growth was evaluated by luciferase live imaging (Berthold, Germany) on day 0, 7, 14, 21, 28, 35 and 42. The mice were observed for mortality up to 49 days after CAR T cell administration. Survival is graphically represented as Kaplan–Meier curves and was analyzed by using the log-rank test.

For evaluating the potential of targeting CD19 of BC19 CAR T cells, Nalm-6 cell based xenograft mouse model was used. 1×10^6 Nalm-6 cell in 0.1 mL was injected into each of female NCG mice (GemPharmatech Co., Ltd.) on day -7. The mice were treated with 2×10^6 BCMA CAR T, CD19 CAR T, BC19 CAR T or mock T cells on day 0, respectively. In vivo tumor growth was evaluated, and mortality of the mice was monitored.

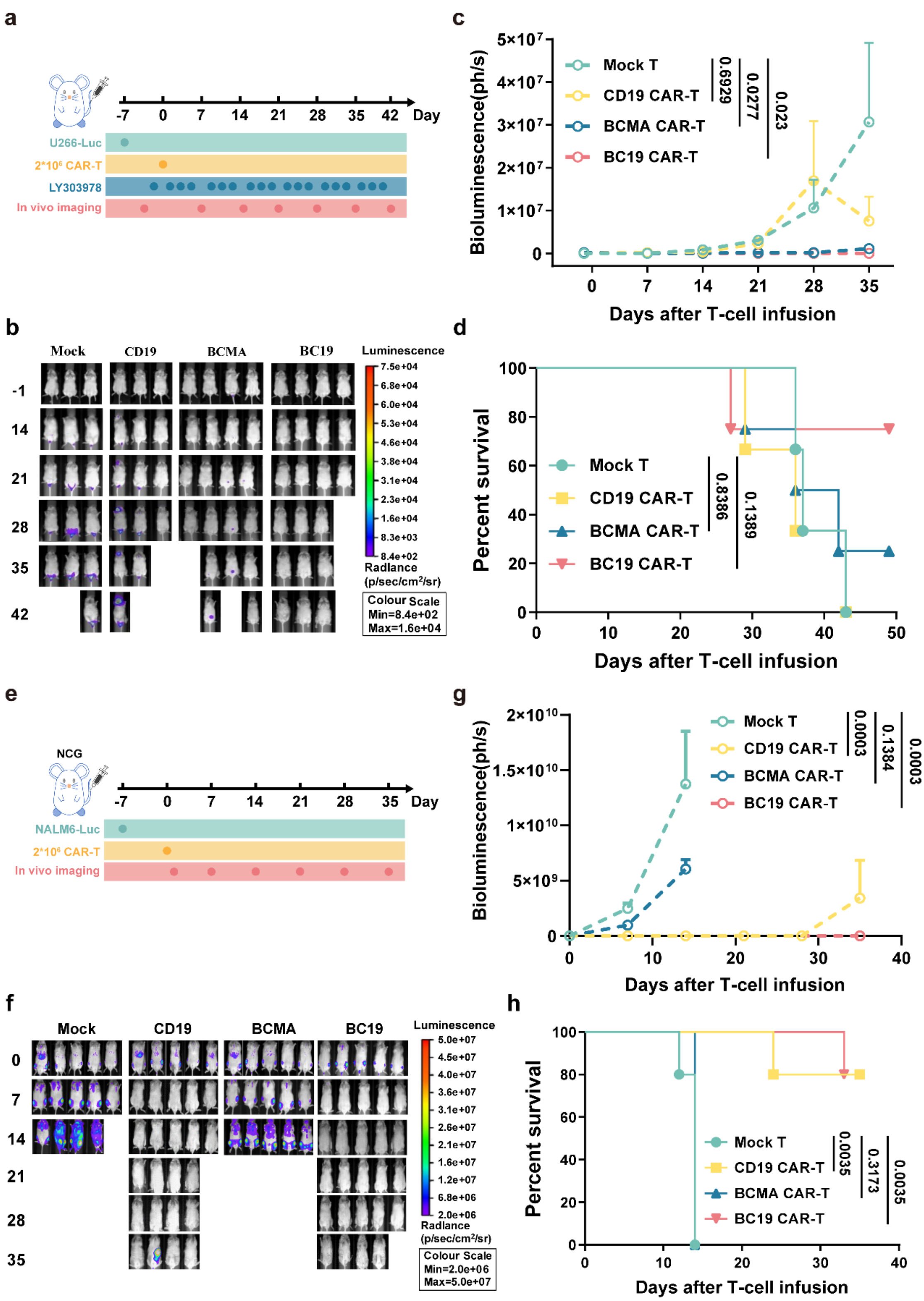

Fig. 2 Evaluating antitumor therapeutic ability of BC19 CAR T cells in vivo.

3.9 Previous clinical study

From May 1, 2017, to Jan 20, 2019, 22 patients were enrolled and 21 received an infusion of

CAR-T cells and were evaluable for safety and activity analyses. At a median follow-up of 179 days (IQR 72–295), 20 (95%) of 21 patients had an overall response, including nine (43%) stringent complete responses, three (14%) complete responses, five (24%) very good partial responses, and three (14%) partial responses. The most common adverse events included cytokine release syndrome (19 [90%] of 21), including 18 patients (86%) with grade 1–2 cytokine release syndrome. The most common serious adverse events were hematological toxicities, which occurred in 20 (95%) of 21 patients. Common grade 3 or higher adverse events included neutropenia (18 [86%]), anemia (13 [62%]), and thrombocytopenia (13 [62%]). One patient died due to cerebral hemorrhage, which was considered related to sustained thrombocytopenia. No deaths were judged to be treatment-related [26].

4. Objectives

This study is an open-label and single-arm clinical study. We attempt to treat patients with R/R MM through BC19 CAR-T cells. The primary endpoint was safety. Safety mainly referred to the severity, frequency and duration of adverse events. Adverse events occurring during the first 3 months after CAR T-cell infusion were monitored continuously. After 3 months, targeted adverse events including hematological events, infections, autoimmune disorders, secondary malignancies, etc. were reported until disease progression separately. The key secondary endpoint was overall response rate (ORR). ORR was defined as the proportion of patients achieving a stringent complete response (sCR), CR, very good partial response (VGPR) or PR according to the IMWG criteria3 at any time after infusion. Other secondary endpoints included duration of response (defined as the time from date of first evidence of achievement of at least PR to disease relapse or progression), progression free survival (defined as the period from CAR T cells infusion to disease progression or death from any cause), OS (defined as the time from infusion to death).

5. Study Design

5.1 Study design

This is an open-label and single-arm to assess the safety and efficacy of infusion of BC19 CAR-T cells in patients with relapsed and refractory multiple myeloma.

5.2 Sample

Number of sample: Approximately 60 subjects will be enrolled into this study.

The total number of patients to be infused with BC19 CAR-T cells is approximately 60. The preliminary I study would enroll 20 subjects depended on practical considerations and the number of potential subjects who meet the minimum requirements of regulation. To further validate the safety and efficacy of BC19 CAR T cell infusion, we plan to expand enrollment to more than 60 patients in a Phase II trial.

5.3 Study duration

The amount of time required to complete this trial will depend on the number of patients. We estimate it will take approximately 4 years to complete this trial.

5.4 Enrollment of patients

Inclusion criteria:

This is an open-label and single-arm clinical study in which all enrolled patients with an age ≥5 and < 70 years met the International Myeloma Working Group (IMWG) diagnostic criteria (Appendix 1)[27]. Patients who were eligible for this study were confirm to be MM through histology, immunology, imaging examination and monoclonal immunoglobulin (or light chain) and had been staged according to the Durie-Salmon (DS) staging system and the International Staging System (ISS) (see Appendix 2). All patients have been treated with alkylators, proteasome inhibitors or autologous hematopoietic stem cell transplantation, and who were refractory to the last line of treatment (response less than partial response (PR) after most recent myeloma therapy or progressive disease (PD) within 60 days after most recent myeloma therapy.)

- Male or female patients aged 5-70;
- The patients' ECOG score is less than or equal to 2, and the estimated survival time is more than 12 weeks;
- Patients diagnosed as refractory or recurrent plasmacytoma, including

(1) Multiple myeloma, plasmacytic leukemia, poems syndrome, megaglobulinemia of Fahrenheit, extramedullary plasmacytoma, solitary plasmacytoma or primary amyloidosis of bone;

(2) Lymphocytic diseases containing plasma cells, such as plasmocytic lymphoma and Castleman's disease.

- Patients with measurable or evaluable lesions;
- The function of main tissues and organs of the patient is good:

(1) Liver function: ALT / AST < 3 times the upper limit of normal value (ULN);

(2) Renal function: creatinine < 220 μ mol / L;

(3) Pulmonary function: indoor oxygen saturation >= 95%;

(4) Cardiac function: left ventricular ejection fraction (LVEF) >= 40%.

- Patients who are not suitable for hematopoietic stem cell transplantation or who give up transplantation or relapse after transplantation due to limited conditions;
- Patients who can take blood from vein without other contraindications of leukocyte removal;
- Patients or their legal guardians voluntarily participate in and sign the informed consent.

Exclusion Criteria:

- Pregnant or lactating women, or women who have pregnancy plans within half a year;
- Patients with infectious diseases (such as HIV, active hepatitis B or C infection, active tuberculosis, etc.);
- Feasibility evaluation and screening showed that the target lymphocyte was less than 10% transfected or less than 5 times amplified under the co stimulation of CD3 / CD28;
- Patients whose vital signs are abnormal and who cannot cooperate with the examination;
- Patients with mental or psychological diseases who can not cooperate with treatment and efficacy evaluation;
- High allergic constitution or severe allergic history, especially for IL-2 allergy;
- Subjects who need anti infection treatment for systemic infection or local severe infection;
- Patients with dysfunction of heart, lung, brain, liver, kidney and other important organs;
- Patients who cannot be included in the treatment for other reasons.

Suspension criteria

Subjects who do not complete the protocol are considered to stop the trial as soon as possible. Causes of premature termination (e.g., voluntary evacuation, toxic side effects, and deaths) must be documented in case reports. The research evaluation will be completed at the end of the suspension. Potential reasons for premature suspension include:

- The follow-up of this subject failed.
- The main researchers believe that the disease is too serious to continue.
- Patients do not comply with the treatment and clinical agreement of the study;
- Pregnancy;
- Voluntary withdrawal. Subjects may withdraw from research projects at any time by changing

their will.

- The significant and rapid development of malignant tumors and metastasis of central nervous system need to choose other methods, including radiotherapy or surgical treatment, but not limited to these two methods.
- Technical difficulties encountered in the process of T cell gene modification and amplification hinder the production of clinical cell doses that meet the quality control standards.
- In addition, if the number of patients with serious adverse events related to cell infusion exceeds 40% of the patients in the enrolled group, the researcher will suspend the study and conduct the corresponding demonstration to decide whether to continue the project.

6. Study Methodology

This study consists of 4 steps: recruitment and Screening of Subjects; preparation of CAR-T infusion (including leukocyte apheresis, CAR-T cell preparation and chemotherapy); CAR-T cell infusion; post-treatment assessment and follow-up.

6.1 Recruitment and Screening of Subjects

First, evaluate the patient's medical history to see if the disease meets the indication requirements; then explain the treatment process and risks, and patient sign an informed consent form. Blood samples will be taken and tested for HIV and sent to the CAR-T production department for T cell production feasibility assessment. About 1-2 weeks, proliferation and transduction efficiency of the patient's T cells will be assessed by production department in vitro to confirm whether his/her T cells are suitable for large-scale CAR-T cell production.

6.2 Examination and data collection

6.2.1 The patients who pass the previous stage will be examined for diseases and a series of routine examinations. See the following examination items for details. The results of the examination items will be collected. According to the Inclusion criteria and exclusion criteria, the suitability of CAR-T cell therapy will be confirmed.

6.2.2 Screen tests (the following tests must be completed within 4 weeks after enrollment):

- Complete history and physical examination.
- Assessment of cardiac function by electrocardiogram and MUGA scans or cardiac ECHO

- Pulse Oxygen Saturation Assessment of Pulmonary Function.
- Na, K, BUN, creatinine, Cl, bilirubin, Ca for whole blood cell count, PO4, CO2, LDH, ALT, AST, uric acid, haptoglobin, direct and indirect Coombs tests, beta-2 microglobulin, SPEP, HCG (for premenopausal women), immunoglobulin levels (IgG, IgM and IgA), free light chain, PT/APTT and INR.
- Bone marrow aspiration, cytogenetics including FISH.
- To quantify the number of T cells and the ratio of CD4:CD8 of Peripheral blood by flow cytometry.
- Detection of HIV, hepatitis B (HepBS Ab, Hep BS Ag, Hep Be Ab, Hep Be Ag and HepBc Ab), and hepatitis C (Hep C Ab).

6.3 CAR-T cell production

Enriched monocytes from healthy donors or patients were further isolated via density gradient centrifugation (Lymphoprep, Axis Shield, Norway) at 800 g without break for 20 min at room temperature. The interface, peripheral blood mononuclear cell (PBMC) layer, was collected and washed with 0.9% Saline. CD3+ T cell purification and activation were conducted with magnetic Dynabeads (Thermo Fisher Scientific) following the manufacturer's instructions. At 24 hours after activation, T cells were transduced with CD19 lentivirus (MOI=5). Briefly, 3×10^6 T cells were mixed with 100 μL of lentivirus (10^8 TU/mL) and centrifuged at 800 g for 60 min at 30°C. After centrifugation, the cell culture flask was returned to a 37°C and 5% CO2 incubator. The transduced T cells were continuously cultured for 5-10 days prior to the subsequent assay.

6.4 Lymphodepleting Chemotherapy

The aim of lymphodepleting chemotherapy is to reduce the burden of tumors and deplete the endogenous lymphocytes, so as to facilitate the proliferation of reinfused CAR-T cells. All patients will be pretreated with FC regimen, fludarabine: $30mg/m^2\times$ 3 days, cyclophosphamide: $750mg/m^2\times1$ day. Antiemetic and symptomatic treatment can be given in the course of chemotherapy. In addition to contra-indications, general treatment is the same as other chemotherapy. If neutrophils are less than 1.0 $\times10^9$/L after chemotherapy, granulocyte colony stimulating factor should be applied to neutrophils more than 1.5×10^9/L. Antibiotics can be given to prevent infection if patients have neutrophil deficiency.

6.6 Assessment post chemotherapy

After chemotherapy, tumor burden should be re-evaluated. Screen tests should include imaging diagnosis, physical examination, blood laboratory testing, bone marrow MRD assessment, and chemotherapy side effects assessment.

6.7 CAR-T cell infusion

CAR-T cells will be infused two days after the end of chemotherapy. The CAR-T cells are cryopreserved in insoluble cryomedia and will be administered as a single dose. The entire rewarm process should be within 10-15 minutes. During the entire infusion process, the patient's vital signs should be closely monitored. The oxygen saturation test should be performed 15 minutes before the infusion, at the end of the infusion, 15 minutes after the infusion, and until the patient's condition is stable. 30 to 60 minutes before infusion of CAR-T cells, patients will be given 325 to 650 mg of acetaminophen to prevent infusion-related reactions. If fever occurs on the day of transfusion of CAR-T cells and lasts less than 24 hours without any other toxicities it is attributed to the transfusion of T-cell response.

6.8 Proliferation of CAR-T cells

To assess the in vivo amplification of CAR-T cells, we designed primers for BC19 CARs and probes with fluorophores to detect the proliferation of BC19 CAR-T cells in peripheral blood by quantitative PCR and detailed process are included in the supplementary materials. T lymphocytes and B lymphocytes in peripheral blood will be detected according to the procedure with details in the Appendix 4.

6.9 Concomitant medication

All kinds of chemotherapeutic drugs are not allowed in patients with CAR-T cell infusion. The complications of patients with CAR-T cell infusion can be treated symptomatically. When severe cytokine release syndrome is difficult to control with other drugs, tocilizumab or glucocorticoid should be chosen. Treatment-related events will be managed as per the treatment algorithm (see section 11.9 Risk disposal SOP).

7. Observation schedule

Comprehensive chemistry profiles, including LDH, phosphorus, and uric acid, creatinine, liver transaminase, bilirubin, complete blood counts, IL-6, ferritin, C-reactive protein (CRP) and coagulation profiles will be obtained every 2-3 days during treatments for one month after CAR-T cell infusion. Observation indicators and examination time are carried out according to the requirements of follow-up time.

8. Follow-up

Efficacy will be assessed at 2 weeks, 1 month, 2-month, 3 month and every three months after the first three months by medical history assessment, physical examination and blood examination. Participants will enter a at least five-year long-term follow-up to assess outcomes such as recurrence of malignant tumors.

9. Clinical efficacy evaluation

The response will be assessed according to the IMWG criteria[28] with details in the Appendix (Appendix 5,6). Re-evaluation of the response will be performed two weeks, 1 month, 2 months, 3 months, and every three months after the first three months of CAR-T infusion. The evaluation includes: the number of plasma cells in the bone marrow determined by morphology, serum paraprotein and serum immunoglobulin concentration measured by immunoturbidimetry, quantitation of 24-h urine protein, M-protein determined by immunofixation electrophoresis, and serum free immunoglobulin light chains. Minimal residual disease (MRD) will be detected according to the standard proposed by Dongen et al.[29] with details in the Appendix 7. In patients with extramedullary disease, the assessment includes imaging techniques (MRI, CT, or PET-CT), physical examination, and biopsy of involved organs for pathological examination when necessary. Patients should be reassessed and given salvage therapy under the condition of disease progression or relapse at any time.

10. Safety evaluation

1) Detection of CRS-related factors (detail see 11.9.2 Cytokine Release Syndrome).

2) Detection of various laboratory items: blood routine, coagulation function, blood piece observation, liver function, kidney function, electrolyte, blood sugar, myocardial enzymes, T cell subsets,

immunoglobulin, etc.

3) Observation of adverse events and serious adverse events. Adverse events occurring during the first 3 months after CAR-T-cell infusion will be monitored continuously. After 3 months, targeted adverse events including hematological events, infections, autoimmune disorders, secondary malignancies, etc. should be reported separately until disease progression.

11. Project risk and SOP

11.1 Project risk

Safety assessment involves all patients receiving CAR-T cell infusion. We will summarize the treatment-related adverse events, severe adverse events, treatment-related serious adverse events, safety laboratory parameters, and classify the adverse events according to NCI CTCAE version 4.0[30] and the CRS evaluation criteria proposed by Lee et al[31].

11.2 Adverse event (AE)

Adverse events (AEs) refer to any adverse medical manifestations, all abnormal findings, subjective and objective symptoms, complications and accidents that occur during cell infusion. Any aggravation of an existing condition or disease is considered an adverse event. Abnormal laboratory results and changes in ECG are considered adverse events only when they lead to discontinuation of treatment, need therapeutic intervention, or what researchers consider to be AEs.

11.3 Observation of adverse events

11.3.1 Clinical adverse events

All participants will be carefully observed for any adverse events during the study, and their clinical manifestations, severity, occurrence time, duration, treatment methods and prognosis were recorded in time (Appendix 8). The correlation between adverse events and experiments will also be determined. All adverse events will be followed up until relapses of disease or death.

11.3.2 Abnormal laboratory test

Those with abnormal results should be closely followed up until they returned to normal, and the

correlation between the abnormal results and the treatment should be determined.

11.4 Severity of AE

Table 1-Severity of AE

**mild**	Transient and tolerate
**moderate**	Uncomfortable, normal activities are affected
**Severe**	Normal activities are severely limited, may cause disability and life threatening
**Life threatening**	Life threatening immediately
**dead**	Correlated with AEs

11.5 Criteria for the relationship between AEs and experimental treatment

11.5.1 Criteria for evaluating adverse events (including symptoms, signs, test indicators)

- The time of occurrence of adverse events coincided with the time of cell therapy.
- AEs are associated with known adverse reactions to cell therapy
- AEs cannot be explained by other reasons.
- AEs disappeared after cell arrest therapy
- Reproduction of AEs after cell therapy

Table 2 - Modified Karch and Lasagna (1975) versions describe the relationship between adverse events and the drug being tested

**Definite**	A response occurs when the drug is taken for a short period of time, or when the drug accumulates a certain level in the body fluid or tissue, triggering a known or expected response pattern of the suspected drug; and the response improves precisely after withdrawal or reduction of the dose, but the response reappears after repeated use.
**Credible**	A response occurs when the drug is taken for a short period of time or when the drug accumulates a certain level in the body fluid or tissue, triggering a known or expected response pattern of the suspected drug; and the response will definitely improve after withdrawal or reduction of the dose, but it cannot be reasonably explained by the clinical condition known to the subject.

**Possible**	A reaction occurs in a short time after taking the drug under test because the suspected drug has a known or expected response pattern, but the reaction can also be easily triggered by other factors.
**inaccessible**	An association cannot be established by evaluation.
**Unrelated**	A reaction in which there is sufficient information to indicate that the cause of the disease is not related to the drug being tested.

11.5.2 Adverse reaction assessment

- Definite correlation: meeting the above criteria 1, 2, 3, 4 and 5
- Possibly correlated: meeting the above criteria 1, 2, 3 and 4
- Inaccessible: at the same time meet the above standards 1 and 2
- May unrelated: meeting the above-mentioned Article 1 criteria
- Definite unrelated: none of the five criteria mentioned above is in conformity with it.

Table 3-Assessment of AE

Results	Indicators				
	1	2	3	4	5
Definite	+	+	+	+	+
Credible	+	+	+	+	?
Possible	+	+	±	±	?
Inaccessible	+	–	±	±	?
Unrelated	+	–	–	–	–

11.6 Record of AEs

11.6.1 AEs

All AEs should be recorded in the CRF and the following should be provided as far as possible:

- The nature of adverse events
- The time when adverse events first occurred
- The intensity of adverse events
- Relations with CAR-T therapy
- The duration of adverse events

- Whether the event is one-off or intermittent (theoretically, every adverse event should be reported). However, some AEs may occur frequently, such as vomiting or diarrhea; if the interval between intermittent events is less than 24 hours, it is more reasonable to record them as an intermittent adverse event.)
- Severe/non-severe

11.6.2 Severe Adverse Events (SAEs)

SAEs are events that require hospitalization, prolong hospitalization time, impair work ability, endanger life or death, and cause congenital malformations during clinical trials. Any serious adverse event that occurs during the treatment or observation phase, whether or not it is related to the research drug, must be reported by fax to the coordinating organization/its representative within 24 hours of his/her discovery.

Researchers are required to record all serious adverse events in the CRF Serious Adverse Events Page and fax them to the coordinating organization/its representative to report serious adverse events. Researchers should not delay notifying coordinating organizations/their representatives of serious adverse events while waiting for other information to complete all records. The information contained in the initial notice should at least be sufficient to illustrate the following:

- Number
- The abbreviation of the subject's name (for reasons of confidentiality, the subject's name should not be transmitted)
- The time and date of the first study drug use
- The time and date of the incident
- A brief description of what happened and the countermeasures taken
- Researchers' opinions on the relationship between events and research drugs

After the first fax report, the CRF Serious Adverse Events Page should be used to further report the adverse events, detailing the questionable parts of the adverse events. Relevant hospital case records and anatomical reports should be provided as far as possible. Coordination organizations should report SAEs to local regulator in accordance with local regulations. Researchers must report serious adverse events to regulatory authorities (if applicable) and ethics committees (IRB) in accordance with local laws and regulations.

11.7 Follow-up observation of subjects with non-severe and severe adverse events

All subjects with non-severe and severe adverse events, whether or not they are associated with the drug, must be monitored. Until the symptoms subside and the relevant clinical laboratory parameters return back to baseline, or observed changes are satisfactorily explained, or death, pathological reports should be provided if feasible. All results must be reported in the files.

11.8 Adverse Event Collection Time

From the beginning of the study to the end, all non-severe adverse events occurring throughout the study need to be collected, whether they are regular telephone contacts and/or information from research visits or spontaneous reports from recipients.

SAEs are collected from the beginning of CAR-T cell therapy until 3 months after completion or discontinuation of CAR-T cell therapy.

11.9 Risk disposal SOP

The symptoms of cough, low fever, fatigue, muscle pain, shivering, sweating, anorexia, nausea and diarrhea will be closely observed after CAR-T cell infusion, whether the patient has respiratory or heart-related symptoms. The presence of tumor lysis symptoms will be closely observed for the first two weeks after infusion. The main indicators include serum electrolyte, phosphorus, calcium, uric acid, creatinine and lactate dehydrogenase. The subjects were assessed for toxicity according to the plan.

11.9.1 Hypogammaglobulinemia and B/plasma cell aplasia

Transient or permanent host B and plasma cell depletion and hypogammaglobulinemia is also a potential risk with CAR-T-19 cells, since normal B cells express CD19. This is expected to resolve if the CAR-T-19 cells are cleared.

1）Clinical Indications of Intravenous Infusion of Immunoglobulin

①IgG< 2g/L;

②IgG 2-5g/L,Patients with antibody deficiencies and frequent bacterial infections.

2）Dosage of IVIgG: 0.4g-0.6g/kg;

The dosage and times of IVIG depends on IgG concentration or infection profile of patients as per

physicians' discretion.

11.9.2 Cytokine Release Syndrome（CRS）

Researches showed that patients treated with CAR-T-19 may experience CRS, development of which has correlated with disease response. Clinical manifestations include high fevers, fatigue, anorexia, nausea, vomiting, headache, rash, hypotension (occasionally requiring vasopressor support), tachypnea, hypoxia (occasionally requiring ventilator support), delirium and confusion (in several patients), evidence of disseminated intravascular coagulation as well as macrophage activation syndrome (MAS). The CRS has been effectively abrogated with anti-cytokine directed therapy including dexamethasone or tocilizumab in most patients (Appendix 9).

1) Diagnosis

**Organ system**	**Symptoms**
Constitutional	Fever ± rigors, malaise, fatigue, anorexia, myalgias, arthalgias, nausea, vomiting, headache
Skin	Rash
Gastrointestinal	Nausea, vomiting, diarrhea
Respiratory	Tachypnea, hypoxemia
Cardiovascular	Tachycardia, widened pulse pressure, hypotension, increased cardiac output (early), potentially diminished cardiac output (late)
Coagulation	Elevated D-dimer, hypofibrinogenemia ± bleeding
Renal	Azotemia
Hepatic	Transaminitis, hyperbilirubinemia
Neurologic	Headache, mental status changes, confusion, delirium, word finding difficulty or frank aphasia, hallucinations, tremor, dysmetria, altered gait, seizures

2) CRS grade system

**Grade**	**Toxicity**
Grade1	Symptoms are not life threatening and require symptomatic treatment only, eg, fever, nausea, fatigue, headache, myalgias, malaise
Grade 2	Symptoms require and respond to moderate intervention
	Oxygen requirement <40% or
	Hypotension responsive to fluids or low dose2 of one vasopressor or
	Grade 2 organ toxicity

Grade 3	Symptoms require and respond to aggressive intervention
	Oxygen requirement ≥40% or
	Hypotension requiring high dose* or multiple vasopressors or
	Grade 3 organ toxicity or grade 4 transaminitis
Grade 4	Life-threatening symptoms
	Requirement for ventilator support or
	Grade 4 organ toxicity (excluding transaminitis)
Grade 5	Death

3) CRS therapy [32]

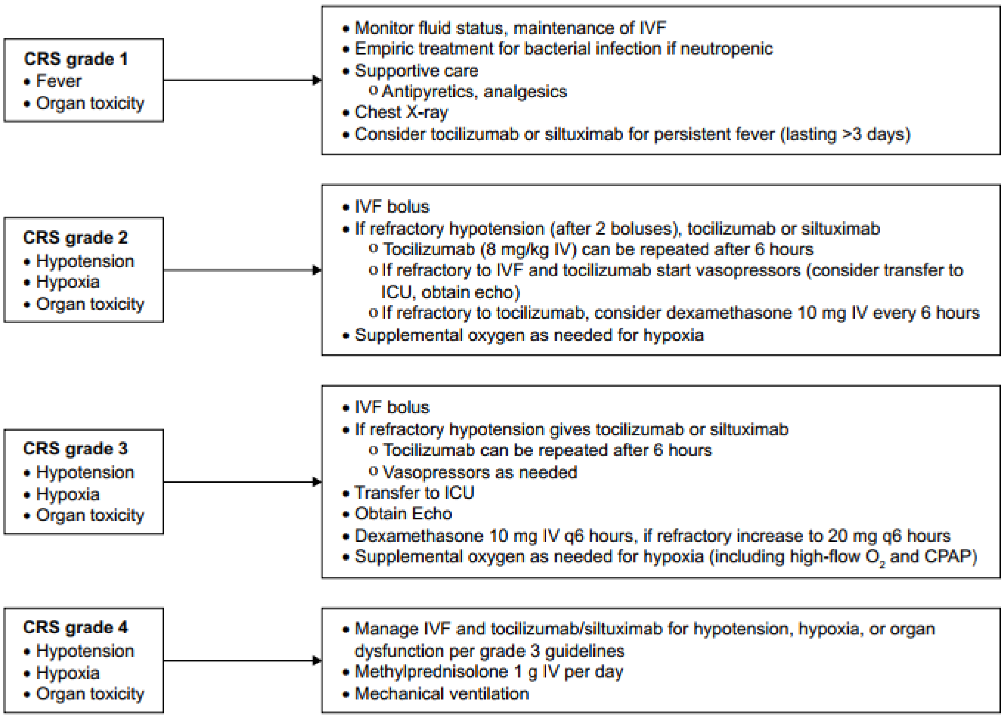

Figure 4 CRS management recommendations by Neelapu et al.[32]
Notes: These recommendations suggest using anti-cytokine therapies for grade 1 CRS and require them for grade 2 or higher CRS. Supportive care is also suggested for each grade.
Abbreviation: CRS, cytokine release syndrome; IVF, intravenous fluid; ICU, intensive care units; q, every; CPAP, continuous positive airway pressure.

11.9.3 Neurological Toxicities

Patients with severe CRS may develop neurological toxicities, including being diminished attention, language disturbance, impaired handwriting, confusion, disorientation, agitation, somnolence, obtundation, myoclonus, encephalopathy, and seizure-like activity. Some patients may require ICU stay due to these side effects, and few patients may require intubation. Neurological examination, brain MRI, and examination of the cerebrospinal fluid (CSF) would be performed as clinically indicated. The

following specific measures will be taken to minimize toxicity in treated patients. Neurologic events will be evaluated according to National Cancer Institute's Common Terminology Criteria for Adverse Events (V 4.03).

Vigilant supportive care is necessary. Intravenous (IV) hydration, withhold oral intake of food, medicines, and fluids, and assess swallowing when necessary. Avoiding medications that cause central nervous system depression. Corticosteroids therapy with dexamethasone 10 mg IV every 6h or methylprednisolone 1 mg/kg IV every 12h may be considered for any severe or life-threatening neurotoxicity and anti-seizure. Sedatives may be considered as clinically indicated. Consider anti-IL-6 therapy with tocilizumab 8 mg/kg IV or siltuximab 11 mg/kg IV, if neurological toxicities are associated with concurrent cytokine-release syndrome (CRS). ICU transfer is recommended for severe neurological toxicities.

11.9.4 Risk of tumor lysis syndrome (TLS)

The risk of tumor lysis syndrome (TLS) is associated with the disease burden, but in most cases, this risk will be very low. The burden of CAR-T-19 target cells in MM patients is low compared with that in CLL patients previously treated, since MM cells does rarely express CD19. Patients will be closely monitored both before and after chemotherapy and CAR-T-19 infusion for laboratory evidence of TLS (serum measurements of potassium, uric acid, phosphate, calcium, and creatinine). Subjects will receive hydration, allopurinol (at the discretion of the treating physician), to minimize any toxicity should significant acute tumor lysis begin to occur (Appendix 10).

11.9.5 Macrophage activation syndrome/hemophagocytic lymphohistiocytosis (MAS/HLH)

Features consistent with MAS/HLH have been observed in patients treated with CAR-T-19, coincident with clinical manifestations of the CRS. MAS appear to be a reaction to immune activation that occurs from the CRS and therefore should be considered a manifestation of CRS. MAS is similar to HLH; it is a rare reaction to immune stimulation by infection, autoimmune diseases or other precipitants, but it is distinct from familial or genetically mediated HLH. The diagnosis of HLH/MAS can be difficult in the context of CRS. Many of the traditional diagnostic criteria for HLH/MAS- fever, splenomegaly, cytopenia in at least two of three hematopoietic cell lineages (red blood cells, white blood cells, and platelets), hyper triglyceridemia or hypofrinogenaemia wit elevated D-dimers, hemophagocytosis in

bone marrow, hyperferritinaemia, high levels of soluble CD25, and low or absent NK-cell activity — are not specific. Indeed, these features are frequently present in patients with even low-grade CRS and also in those with advanced stage hematological malignancies in the absence of CAR-T-cell therapy. Thus, new criteria are needed for the diagnosis of HLH/MAS in patients with CRS after CAR-T-cell therapy.

Diagnostic criteria for CAR-T-cell-related HLH/MAS [32]

A patient might have HLH/MAS if he/she had a peak serum ferritin level of >10,000 ng/ml during the cytokine-release syndrome phase of CAR-T-cell therapy (typically the first 5 days after cell infusion) and subsequently developed any two of the following:

- Grade ≥3 increase in serum bilirubin, aspartate aminotransferase, or alanine aminotransferase levels*
- Grade ≥3 oliguria or increase in serum creatinine levels*
- Grade ≥3 pulmonary edema*
- Presence of haemophagocytosis in bone marrow or organs based on histopathological assessment of cell morphology and/or CD68 immunohistochemistry

*Grading as per Common Terminology Criteria for Adverse Events, version 4.03 (REF. 43)

Studies have shown that IL-6 levels were extraordinarily high during the CRS, prompting us to use an anti-IL6 receptor antibody tocilizumab to treat the CRS/MAS. The majority of patients treated with tocilizumab have rapid (within hours) resolution of dramatic fevers, and continuous improvement in hypotension and hypoxia over hours to several days, and shows improvement in biochemical evidence of CRS and MAS within 48 hours. If the patient has no improvement clinically or serologically within 48 h, additional therapy with etoposide 75-100 mg/m^2 can be considered, as the available evidence in contexts other than CAR-T-cell therapy indicates that this agent is the preferred treatment for refractory HLH. Moreover, this agent can be used in patients with liver and kidney dysfunction. Indeed, rapid initiation of etoposide therapy, in spite of organ dysfunction, may be imperative for patients with high probability of a HLH diagnosis, owing to the high risk of death. Etoposide can be repeated after 4-7 days, as indicated clinically or serologically, to achieve adequate disease control. Intrathecal cytarabine, with or without hydrocortisone, should also be considered for patients with HLH-associated neurotoxicity.

Although etoposide and cytarabine are often used in the treatment of familial and malignancy-associated HLH, at present, direct evidence to support their use in patients with CAR-T-cell-associated HLH is lacking [34-35].

12. Regulation

12.1 Personnel

Each of the medical team consists of at least one senior doctor, two attending physicians, one senior nurse with the title of Vice-Senior or above, and two supervisor nurses. Medical staff should be skilled in aseptic operation and first-aid skills.

12.2 Environment

According to the "Code for Construction and Acceptance of Clean rooms", "Good Manufacturing Practices for Pharmaceutical Production (Revised in 2010)", GMP laboratory that has passed the examination and has an overall Class B local A-level standard. The construction of the lentiviral system and the entire process of CAR-T cell preparation were performed in a GMP laboratory.

12.3 Apparatus

Lentivirus construction and CAR-T cell preparation equipment are standardized. All equipment is regularly maintained and calibrated.

12.4 CAR-T cells preparation

Before infusion, CAR-T products must pass the following test items: bacteria (e.g. faecal alkalogenic bacteria, Candida albicans, Escherichia coli, Haemophilusin fluenzae, Meningococcus, Pseudomonas aeruginosa, Staphylococcus aureus, Streptococcus pneumoniae and Streptococcus pyogenes A, fungi, mycoplasma, endotoxin, replicated lentivirus, p24, VSV-G nucleus, Acids, HIV gag, mouse antibodies, medium components, carrier packaging cells or plasmid components.

13. Files and data

The hospital should keep these original data until 5 years after the termination of the clinical study, including the confirmation of all participants (effective check of different records, such as CRF and

hospital original records), informed consent of all original participants, CRF form, detailed records of drug distribution, etc.

The inspector is responsible for conducting regular on-site inspections to ensure strict compliance and implementation of clinical trial programs. The record tables are checked to ensure that the data on CRF are consistent with the original data, no errors and no tampering. All adverse events are recorded in detail, properly handled and tracked until they are properly resolved. SAE and unexpected events are reported to ethics committees and regulatory authorities in accordance with regulations. Researchers periodically review all adverse events, and assess the risks and benefits of the study when necessary.

14. Statistical Analysis Plan

This is an open label, exploratory development study to evaluate the safety and tolerability, and persistence and engraftment of autologous T cells engineered to express a chimeric antigen receptor targeting BCMA/CD19. The subject population to be analyzed for primary and secondary endpoints will include all patients who were infused with BC19 CAR-T-cells.

14.1 General principles of statistical analysis

Statistical analysis for this study will be performed using SPSS version 22 or above. If there is no special explanation, all hypothesis tests are conducted by two-sided test. P less than 0.05 is defined as statistically significant, and P will be retained to four decimal places if necessary, e.g. P <0.0001. 95% confidence interval should be calculated if necessary. The descriptive analysis of a single group will be carried out in the following manner: continuous variables will be summarized by number of subjects, mean, standard deviation, median, first quartile, third quartile, minimum and maximum; Classification variables will be summarized using the number and the percentage.

14.2 Statistical analysis methods

1) Processing methods for shedding and missing data

The analysis of secondary endpoints will include shedding data. When the intention-to-treat (ITT) set is used for the analysis of secondary study endpoints, the last observation carried forward (LOCF) method is used to impute missing data, in which the last non-missing post-baseline on-treatment measurement at scheduled clinic visits is used to impute the missing measurements.

2) Interim analysis

Not Applicable.

3) Subgroup analysis

Subgroups or covariate analyses of efficacy and / or safety will be needed. The continuous variables are tested by Mann-Whitney U test for two groups and Kruskal-Wallis test for multiple groups. Kaplan-Meier curves are used for analyzing duration of response, progression-free survival and overall survival and are compared with the use of log-rank test. Multivariate Cox Regression models is used for analysis of factors related to survival.

14.3 Statistical analysis

1) Drop out analysis

Classify according to the description of the total number of subjects participating in the inclusion screening and the reasons for exclusion. Subjects' information is summarized in terms of primary causes (e.g., loss to follow-up, AE, poor compliance), including the number of subjects and the number of subjects entering and completing each study phase (or each week/month of the study), as well as reasons for discontinuation after enrollment. Clarify whether subjects who discontinue CAR-T cell therapy are followed up during the study period.

Provide a list of all subjects who are discontinued after enrolment, including subject number, specific reason for discontinuation, treatment (drug/CAR-T cells and dose), cumulative dose (if appropriate), and duration of treatment before discontinuation.

2) Demographic and other baseline characteristics analysis

According to the numerical characteristics of the variables, continuous variables (such as age, height, weight, etc.) will be summarized using number, mean, standard deviation, median, first quartile, third quartile, minimum and maximum; Categorical variables (such as sex, disease history, previous treatment, etc.) will be summarized using the number and percentage. The continuous variables are tested by Mann-Whitney U test for two groups and Kruskal-Wallis test for multiple groups.

3) Efficacy analysis

The overall response rates analysis will be described at different follow-up time points (3, 6, and 1 year) after treatment. Kaplan-Meier curves are used for analyzing duration of response, progression-free survival and overall survival and were compared with the use of log-rank test.

Multivariate Cox Regression models is used for analysis of factors related to survival. Subgroups or covariate analyses of efficacy and / or safety will be needed.

The analysis of the amplification level, distribution characteristics and persistence time of CAR-T cells in subjects should be described according to different follow-up time points after treatment and various tissues in vivo (peripheral blood, bone marrow, cerebrospinal fluid and lymph nodes, etc.), and a line chart should be drawn based on the above corresponding characteristics and follow-up time points.

The characteristics of lymphocyte subduction and retroviral replication in vivo should be described according to different follow-up time points, and a line chart should be drawn according to the corresponding characteristics and follow-up time points.

4) Safety analysis

Safety will be evaluated by summarizing AE, changes in laboratory test results, and changes in vital signs. Any subject treated with CAR-T cell therapy will be included in the safety analysis.

A) Adverse events

Descriptive statistics (counts and percentages) of all adverse events, including long-term adverse events.

B) Laboratory tests

Descriptive statistics (counts and percentages) of the results of all laboratory tests and changes from baseline.

C) Vital signs, physical examinations, and other safety-related examinations

Descriptive statistics (count and percentage) of vital signs test results and changes from baseline. Vital signs, other physical examination findings, and other safety-related observations should be analyzed and presented in a similar manner to laboratory variables.

15. Ethical Considerations

1. This protocol and any amendments will be submitted to a properly constituted independent Institutional Review Board (IRB), in agreement with local legal prescriptions, for formal approval of the study conduct. The decision of the IRB concerning the conduct of the study will be made in writing to the investigator and a copy of this decision will be provided to the sponsor before commencement of this study. Serious adverse events during the study should be reported to the IRB in a timely manner.

2. In the process of clinical research, the individual rights and interests of the subjects must be fully protected, and the scientific nature and reliability of the research must be ensured. The interests, safety and health of the subjects are above the consideration of scientific and social interests.

3. The investigator or his/her designated representative must explain the detailed information about the clinical study to the subjects, and obtain informed consent after fully and in detail explaining the content of the study.

16. Confidentiality

The investigator must ensure anonymity of the patients and patients must not be identified by names in any documents submitted to the funding sponsor. Signed informed consent forms and patient enrollment log must be kept strictly confidential to enable patient identification at the site. Information about study subjects will be kept confidential and managed. In the event that a subject revokes authorization to collect, the investigator, by regulation, retains the ability to use all information collected prior to the revocation of subject authorization. For subjects that have revoked authorization to collect, attempts should be made to obtain permission to collect at least vital status (i.e. that the subject is alive) at the end of their scheduled study period.

17. Faculty

personnel	Position/major	task
Cao Jiang	Director /hematology	Protocol design and supervisor
Xu Kai-lin	Deputy director /hematology	Protocol design
Zheng Jun-nian	Deputy director/oncology	Protocol design
Li Zhen-yu	Deputy director /hematology	Protocol design, toxicity investigation
Yan Zhi-ling	Deputy director of the physician /hematology	Clinical observation
Cheng Hai	Deputy director of the physician /hematology	Clinical observation
Chen Wei	Attending /hematology	Subject screen
Zhang Huanxin	Attending /hematology	Clinical observation
Wang Ying	Attending /hematology	Clinical observation
Sang Wei	Attending /hematology	Clinical observation
Zhu Feng	Attending /hematology	Clinical observation
Xie Xiaobao	Attending /hematology	Clinical observation
Gu Weiying	Attending /hematology	Clinical observation
Lan Jian-ping	Director /hematology	Subject screen
Jin Lai	Director /hematology	Subject screen
Fei Xiaoming	Deputy director of the physician /hematology	Subject screen
Yao Yao	associate research fellow /hematology	CAR-T Cell preparation
Wang Gang	Assistant research fellow/cell therapy	Cell preparation
Shi Ming	Assistant research fellow/cell therapy	CAR-T preparation supervisor
Jing Guangjun	Assistant research fellow/cell therapy	CAR-T Cell preparation
Lu Qun-xian	associate research fell ow/Medical examiner	Tests
Wu Qing-yun	associate research fellow /hematology	CAR-T Cell preparation
Zhao Jing	Nurse supervisor/nursing	Nurse

18. Protocol Reference

1. Attal M, Harousseau JL, Stoppa AM, et al. A prospective, randomized trial of autologous bone marrow transplantation and chemotherapy in multiple myeloma. Intergroupe Francais du Myelome. N Engl J Med. 1996 Jul 11;335(2):91-7.

2. Barlogie B, Jagannath S, Desikan KR, et al. Total therapy with tandem transplants for newly diagnosed multiple myeloma. Blood. 1999 Jan 1;93(1):55-65.

3. Richardson PG, Barlogie B, Berenson J, et al. A phase 2 study of bortezomib in relapsed, refractory myeloma. N Engl J Med. 2003 Jun 26;348(26):2609-17.

4. Rajkumar SV, Blood E. Lenalidomide and venous thrombosis in multiple myeloma. N Engl J Med. 2006 May 11;354(19):2079-80.

5. Lokhorst HM, Plesner T, Laubach JP, et al. Targeting CD38 with Daratumumab Monotherapy in Multiple Myeloma. N Engl J Med. 2015 Sep 24;373(13):1207-19.

6. Palumbo A, Chanan-Khan A, Weisel K, et al. Daratumumab, Bortezomib, and Dexamethasone for Multiple Myeloma. N Engl J Med. 2016 Aug 25;375(8):754-66.

7. McCAR-Thy PL, Owzar K, Hofmeister CC, et al. Lenalidomide after stem-cell transplantation for multiple myeloma. N Engl J Med. 2012 May 10;366(19):1770-81.

8. Krishnan A, Pasquini MC, Logan B, et al. Autologous haemopoietic stem-cell transplantation followed by allogeneic or autologous haemopoietic stem-cell transplantation in patients with multiple myeloma (BMT CTN 0102): a phase 3 biological assignment trial. Lancet Oncol. 2011 Dec;12(13):1195-203.

9. Kumar SK, Dimopoulos MA, Kastritis E, et al. Natural history of relapsed myeloma, refractory to immunomodulatory drugs and proteasome inhibitors: a multicenter IMWG study. Leukemia. 2017 Nov;31(11):2443-8.

10. Brown RD, Spencer A, Ho PJ, et al. Prognostically significant cytotoxic T cell clones are stimulated after thalidomide therapy in patients with multiple myeloma. Leuk Lymphoma. 2009 Nov;50(11):1860-4.

11. Tyler EM, Jungbluth AA, O'Reilly RJ, et al. WT1-specific T-cell responses in high-risk multiple myeloma patients undergoing allogeneic T cell-depleted hematopoietic stem cell transplantation and donor lymphocyte infusions. Blood. 2013 Jan 10;121(2):308-17.

12. Peggs KS, Thomson K, Hart DP, et al. Dose-escalated donor lymphocyte infusions following

reduced intensity transplantation: toxicity, chimerism, and disease responses. Blood. 2004 Feb 15;103(4):1548-56.

13. Sadelain M. T-cell engineering for cancer immunotherapy. Cancer J. 2009 Nov-Dec;15(6):451-5.

14. Sadelain M, Brentjens R, Riviere I. The basic principles of chimeric antigen receptor design. Cancer Discov. 2013 Apr;3(4):388-98.

15. Bhojwani D, Pui CH. Relapsed childhood acute lymphoblastic leukaemia. Lancet Oncol. 2013 May;14(6):e205-17.

16. Geyer MB, Brentjens RJ. Review: Current clinical applications of chimeric antigen receptor (CAR) modified T cells. Cytotherapy. 2016 Nov;18(11):1393-409.

17. Garfall AL, Maus MV, Hwang WT, et al. Chimeric Antigen Receptor T Cells against CD19 for Multiple Myeloma. N Engl J Med. 2015 Sep 10;373(11):1040-7.

18. Ramos CA, Savoldo B, Torrano V, et al. Clinical responses with T lymphocytes targeting malignancy-associated kappa light chains. J Clin Invest. 2016 Jul 1;126(7):2588-96.

19. Carpenter RO, Evbuomwan MO, Pittaluga S, et al. B-cell maturation antigen is a promising target for adoptive T-cell therapy of multiple myeloma. Clin Cancer Res. 2013 Apr 15;19(8):2048-60.

20. Ali SA, Shi V, Maric I, et al. T cells expressing an anti-B-cell maturation antigen chimeric antigen receptor cause remissions of multiple myeloma. Blood. 2016 Sep 29;128(13):1688-700.

21. Yaccoby S. The phenotypic plasticity of myeloma plasma cells as expressed by dedifferentiation into an immature, resilient, and apoptosis-resistant phenotype. Clin Cancer Res. 2005 Nov 1;11(21):7599-606.

22. O'Connor BP, Raman VS, Erickson LD, et al. BCMA is essential for the survival of long-lived bone marrow plasma cells. J Exp Med. 2004 Jan 5;199(1):91-8.

23. Chauhan D, Singh AV, Brahmandam M, et al. Functional interaction of plasmacytoid dendritic cells with multiple myeloma cells: a therapeutic target. Cancer Cell. 2009 Oct 6;16(4):309-23.

24. Ryan MC, Hering M, Peckham D, et al. Antibody targeting of B-cell maturation antigen on malignant plasma cells. Mol Cancer Ther. 2007 Nov;6(11):3009-18.

25. Xu S, Lam KP. B-cell maturation protein, which binds the tumor necrosis factor family members BAFF and APRIL, is dispensable for humoral immune responses. Mol Cell Biol. 2001 Jun;21(12):4067-74.

26. Yan Z, Cao J, Cheng H, et al. A combination of humanised anti-CD19 and anti-BCMA CAR-T

cells in patients with relapsed or refractory multiple myeloma: a single-arm, phase 2 trial. Lancet Haematol 2019;6:e521-e9.

27. Rajkumar SV, Dimopoulos MA, Palumbo A, et al. International Myeloma Working Group updated criteria for the diagnosis of multiple myeloma. Lancet Oncol. 2014 Nov;15(12):e538-48.

28. Palumbo A, Rajkumar SV, San Miguel JF, et al. International Myeloma Working Group consensus statement for the management, treatment, and supportive care of patients with myeloma not eligible for standard autologous stem-cell transplantation. J Clin Oncol. 2014 Feb 20;32(6):587-600.

29. van Dongen JJ, Lhermitte L, Bottcher S, et al. EuroFlow antibody panels for standardized n-dimensional flow cytometric immunophenotyping of normal, reactive and malignant leukocytes. Leukemia. 2012 Sep;26(9):1908-75.

30. Institute NC. Common terminology criteria for adverse events (CTCAE) v4.0. 2010.

31. Lee DW, Gardner R, Porter DL, et al. Current concepts in the diagnosis and management of cytokine release syndrome. Blood. 2014 Jul 10;124(2):188-95.

32. Neelapu SS, Tummala S, Kebriaei P, et al. Chimeric antigen receptor T-cell therapy – assessment and management of toxicities. Nat Rev Clin Oncol. 2018;15(1):47–62.

33. Brudno JN & Kochenderfer JN. Toxicities of chimeric antigen receptor T cells: recognition and management. Blood. 2016; 127: 3321–3330.

34. Schram AM & Berliner N How I treat hemophagocytic lymphohistiocytosis in the adult patient. Blood. 2015; 125: 2908–2914.

35. Jordan MB, Allen CE, Weitzman S, Filipovich AH & McClain KL. How I treat hemophagocytic lymphohistiocytosis. Blood.2011; 118: 4041–4052 .

19. Appendix

Appendix 1

Revised International Myeloma Working Group Diagnostic Criteria For Multiple Myeloma And Smouldering Multiple Myeloma

Definition of multiple myeloma

Clonal bone marrow plasma cells ≥10% or biopsy-proven bony or extramedullary plasmacytoma and any one or more of the following myeloma defining events:

Myeloma defining events:

Evidence of end organ damage that can be attributed to the underlying plasma cell proliferative disorder, specifically:

- Hypercalcaemia: serum calcium >0.25 mmol/L (>1 mg/dL) higher than the upper limit of normal or >2.75 mmol/L (>11 mg/dL)
- Renal insufficiency: creatinine clearance <40 mL per min† or serum creatinine>177 µmol/L (>2 mg/dL)
- Anemia: hemoglobin value of >20 g/L below the lower limit of normal, or a hemoglobin value <100 g/L
- Bone lesions: one or more osteolytic lesions on skeletal radiography, CT, or PET-CT

Any one or more of the following biomarkers of malignancy:

- Clonal bone marrow plasma cell percentage ≥60%
- Involved: uninvolved serum free light chain ratio ≥100
- >1 focal lesion on MRI studies

Definition of smouldering multiple myeloma

Both criteria must be met:

- Serum monoclonal protein (IgG or IgA) ≥30 g/L or urinary monoclonal protein ≥500 mg per 24 h and/or clonally bone marrow plasma cells 10–60%
- Absence of myeloma defining events or amyloidosis

Appendix 2

Staging Systems for Multiple Myeloma

Stage	Durie-Salmon Criteria
I	All of the following:<br>• Hemoglobin value >10 g/dL<br>• Serum calcium value normal or ≤12 mg/dL<br>• Bone x-ray, normal bone structure) or solitary bone plasmacytoma only<br>• Low M-component production rate：<br>IgG value <5 g/dl;<br>IgA value <3 g/dl<br>Bence Jones protein <4 g/24 h
II	Neither stage l nor stage lll
III	One or more of the following:<br>• Hemoglobin value <8.5 g/dl<br>• Serum calcium value >12 mg/dl<br>• Advanced lytic bone lesions<br>• High M-component production rate：<br>IgG value >7 g/dl;<br>IgA value >5 g/dl<br>Bence Jones protein >12 g/24 h
Subclassifiation Criteria<br>A Normal renal function (serum creatinine level <2.0 mg/dl)<br>B Abnormal renal function (serum creatinine level ≥2.0 mg/dl)	

Stage	International Staging System
I	Serum β2-microglobulin <3.5 mg/L and serum albumin ≥ 3.5 g/dL
II	Serum β2-microglobulin < 3.5 mg/L and serum albumin < 3.5 g/dL or serum β2-microglobulin 3.5 to < 5.5 mg/L
III	Serum β2-microglobulin ≥ 5.5 mg/L

Appendix 3

ECOG performance status

Grade	ECOG performance status
0	Fully active, able to carry on all pre-disease performance without restriction
1	Restricted in physically strenuous activity but ambulatory and able to carry out work of a light or sedentary nature, e.g., light house work, office work
2	Ambulatory and capable of all self-care but unable to carry out any work activities; up and about more than 50% of waking hours
3	Capable of only limited self-care; confined to bed or chair more than 50% of waking hours
4	Completely disabled; cannot carry on any self-care; totally confined to bed or chair
4	Dead

Appendix 4

Car-T Proliferation Examination

The proliferation of CAR-T cells was constantly monitored after adoptively infusion into patients. The PBMCs were harvested from patients periodically and the average copy number of CAR gene in patients was determined by quantitative real-time PCR (QPCR). Briefly, the genomic DNA was purified using a Genomic DNA Purification Kit (Thermo Fisher Scientific). The plasmid containing human albumin or CAR was used to establish standard curves. The albumin gene served as an internal control due to the presence of its sequence in two copies per genome.

Procedure

1．DNA preparation

Genomic DNA extraction kit was used to extract genomic DNA from cells.

2. Standard curve

(1) The concentration of plasmid template (pAlb and LTR) was first adjusted to 1 mg/mL, and then the number of copies of the sample was calculated according to the number of bases in the plasmid.

(2) The first point of the standard curve is 1 x 107 copies, then diluted by 10 times gradient, and the last point is 103 copies to produce the standard curve.

(3) Gradient dilution and standard curve are made according to the way shown below.

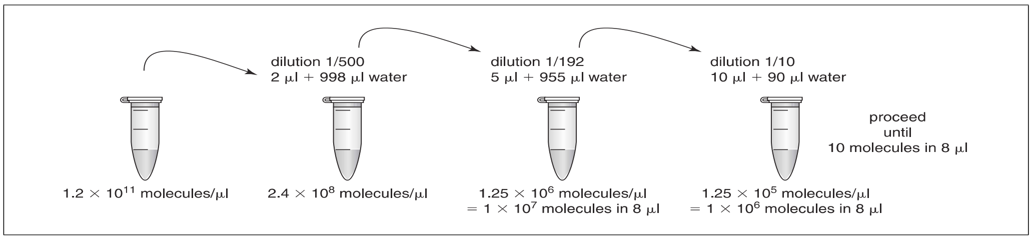

The template samples with different dilution multiples were used for quantitative PCR.

3. Quantitative PCR

PCR reaction system

The total amount required for the sample, Control and NTC (No Template Controls) was calculated, with two compound holes in each group.

Premixtures of probes and primers prepared separately

Table

- primers

Primer	5'-3'	fluore
BC19 CAR F	CAGGCTCCTCGCCTTCTG	
BC19 CAR R	GAACCTGGCAGGGATTCCA	
BC19 CAR Probe	TCTACCACACCAGCCGGCTCCATT	5'-FAM；3'-BHQ1
ALB F	GCTGTCATCTCTTGTGGGCTGT	
ALB R	ACTCATGGGAGCTGCTGGTTC	
ALB Probe	CCTGTCATGCCCACACAAATCTCTCC	5'-FAM-BHQ1-3'

- PCR mix

Reagent	Volume (μL) per Single 20 μL Reaction
2×PCR Mix	10
Probe	0.4
Primer mix F&R	0.4
PCR grade water	4.2

(1) The above 15uL PCR mix was added to 96-well plate. At the same time, according to plate map, 5 ug sample DNA (total 10 ng-100 ng) was added into each hole, 5 uL water was added into NTC group, and the final concentration of primers and probes was 200 nM.

(2) After sealing, centrifuge 200 g for 1 min.

(3) Setting up the procedure of PCR reaction as follows:

	Temp (°C)	Time (sec)
Initial Hot Start/denaturation	95	30
Steps 1–2 are repeated through 40 cycles		
Step 1	95	5
Step 2	60	30

Appendix 5

Response Criteria for Multiple Myeloma (Revised Uniform Response Criteria By The International Myeloma Working Group)

Response Category	Response Criteria
CR, complete response	Negative immunofixation of serum and urine, disappearance of any soft tissue plasmacytomas, and <5% plasma cells in bone marrow; in patients for whom only measurable disease is by serum FLC level, normal FLC ratio of 0.26 to 1.65 in addition to CR criteria is required; two consecutive assessments are needed
sCR, stringent complete response	CR as defined plus normal FLC ratio and absence of clonal plasma cells by immunohistochemistry or two- to four-color flow cytometry; two consecutive assessments of laboratory parameters are needed
Immunophenotypic CR	sCR as defined plus absence of phenotypically aberrant plasma cells (clone) in bone marrow with minimum of 1 million total bone marrow cells analyzed by multi-parametric flow cytometry (with >four colors)
Molecular CR	CR as defined plus negative allele-specific oligonucleotide polymerase chain reaction (sensitivity 10-5)
VGPR, very good partial response	Serum and urine M component detectable by immunofixation but not on electrophoresis or ≥90% reduction in serum M component plus urine M component <100 mg/24 h; in patients for whom only measurable disease is by serum FLC level, >90% decrease indifference between involved and uninvolved FLC levels, in addition to VGPR criteria, is required; two consecutive assessments are needed
PR, partial response	≥50% reduction of serum M-protein and reduction in 24-hour urinary M-protein by ≥90% or to <200 mg per 24 hIf the serum and urine M-protein are unmeasurable, a ≥50% decrease in the difference between involved and uninvolved FLC levelsis required in place of the M-protein criteria. If serum and urine M-protein are unmeasurable, and serum free light assay is also unmeasurable, ≥50% reduction in plasma cells is required in place of M-protein, provided baseline bone marrow plasma cell percentage was ≥30%. In addition, if present at baseline, a ≥50% reduction in the size of soft tissue plasmacytomas is also required.<br>Two consecutive assessments are needed; no known evidence of progressive or new bone lesions if radiographic studies were performed.
MR, minimal response for	≥25% but ≤49% reduction of serum M protein and reduction in 24-hour urine M protein by 50% to 89%

relapsed refractory myeloma only	In addition, if present at baseline, 25% to 49% reduction in size of soft tissue plasmacytomas is also required<br>No increase in size or number of lytic bone lesions (development of compression fracture does not exclude response)
SD, stable disease	Not meeting criteria for CR, VGPR, PR or progressive disease; no known evidence of progressive or new bone lesions if radiographic studies were performed
PD, progressive disease	Increase of 25% from lowest response value in any of following:<br>Serum M component with absolute increase ≥0.5 g/dL; serum M component increases ≥1 g/dL are sufficient to defined relapse if starting M component is ≥5 g/dl and/or;<br>Urine M component (absolute increase must be ≥200 mg/24 h) and/or;<br>Only in patients without measurable serum and urine M protein levels: difference between involved and uninvolved FLC levels (absolute increase must be >10 mg/dl);<br>Only in patients without measurable serum and urine M protein levels and without measurable disease by FLC level, bone marrow plasma cell percentage (absolute percentage must be ≥10%)<br>Development of new or definite increase in size of existing bone lesions or soft tissue plasmas<br>Development of hypercalcemia that can be attributed solely to plasma cell proliferative disorder<br>Two consecutive assessments before new therapy are needed

Appendix 6

Response Criteria For Multiple Myeloma（Relapsed）

Relapse Subcategory	Relapse Criteria
Clinical relapse	Clinical relapse requires one or more of:<br>•Direct indicators of increasing disease and/or end organ dysfunction (CRAB features). It is not used in calculation of time to progression or progression-free survival but is listed here as something that can be reported optionally or for use in clinical practice<br>• Development of new soft tissue plasmacytomas or bone lesions<br>• Definite increase in the size of existing plasmacytomas or bone lesions. A definite increase is defined as a 50% (and at least 1 cm) increase as measured serially by the sum of the products of the cross-diameters of the measurable lesion<br>• Hypercalcemia (>11.5 mg/dL) [2.65 mmol/L]<br>• Decrease in hemoglobin of ≥2 g/dL [1.25 mmol/L]<br>• Rise in serum creatinine by 2 mg/dL or more [177 µmol/L or more]
Relapse from CR	Any one or more of the following:<br>• Reappearance of serum or urine M-protein by immunofixation or electrophoresis<br>• Development of ≥5% plasma cells in the bone marrow<br>• Appearance of any other sign of progression (ie, new plasmacytoma, lytic bone lesion, or hypercalcemia)

Appendix 7

MRD Detection

Bone marrow was collected at different follow-up time after treatment. MRD was detected according to EuroFlow antibody panels for plasma cell disorders (PCD).

Multiple myeloma Flow-MRD: antibody panel.

**Tube**	**1**	**2**
Qdot 565	CD45	CD45
APC-CY7	CD38	CD38
APC	CD138	CD138
PerCP-CY5.5	CD19	CD19
PE-CY7	BCMA	BCMA
BV510	CD27	
V450	CD56	
FITC		CyIgκ
PE		CyIgλ

Procedure

Flow cytometric (FCM) analysis was performed on fresh specimens and isotype control antibody was used in each sample for normalization. Plasma cells were gated according to the expression of CD45 and CD38. Phenotype of plasma cells was defined as low level of CD45 and intermediate-to-high level of CD38, which was abbreviated as CD45lowCD38$^{int/hi}$ thereafter. Plasma cells were further analyzed for the expression of other markers such as BCMA and CD19. No less than 1.0×10^5 cells were acquired in FCM analysis for each sample.

Appendix 8

Adverse Event Recording

All adverse events occurring during the adverse event reporting period must be recorded. Adverse events (including lab abnormalities that constitute AEs) should be described using a diagnosis whenever possible, rather than individual underlying signs and symptoms. When a clear diagnosis cannot be identified, each sign or symptom should be reported as a separate Adverse Event. All adverse events should be treated appropriately

As far as possible, each adverse event should be evaluated to determine:

1. The severity grade (CTCAE4.0)
2. Its duration (start and end dates)
3. Its relationship to the study treatment: Is there a reasonable possibility that the AE is related to the study treatment- No (unrelated) or Yes. If yes is the event possibly, probably or definitely related to the investigational treatment (CAR-T19 T-cells) or the non-investigational treatment (i.e. lymphodepleting chemotherapy).
4. Action taken with respect to study or investigational treatment (none, dose adjusted, temporarily interrupted, permanently discontinued, unknown, not applicable)
5. Whether medication or therapy taken (i.e. no concomitant medication/non-drug therapy, concomitant medication/non-drug therapy)

Appendix 9

CRS Grading and Management

Grade	Criteria
1	Mild reaction:<br>Treated with supportive care such as antipyretics, antiemetics.
2	Moderate reaction<br>requiring IV therapies or parenteral nutrition; some signs organ dysfunction (i.e. grade 2 creatinine or grade 3 liver function tests) related to CRS and not attributable to any other condition. Hospitalization for management of CRS related symptoms including fevers associated neutropenia
3	More severe reaction:<br>Hospitalization required for management of symptoms related to organ dysfunction including grade 4 LFTs or grade 3 creatinine related to CRS and not attributable to any other conditions; this excludes management of fevers or myalgias. Includes hypotension treated with IVFs or low-dose pressors, coagulopathy requiring fresh frozen plasma (FFP) or cryoprecipitate, and hypoxia requiring supplemental oxygen (nasal cannula oxygen, high flow oxygen, Continuous Positive Airway Pressure [CPAP] or Bilateral Positive Airway Pressure [BiPAP]). Patients admitted for management of suspected infection due to fevers and/or neutropenia may have grade 2 CRS.
4	Life-threatening complications such as hypotension requiring pressors, hypoxia requiring mechanical ventilation.
5	Death

Appendix 10

SOP of Serious Adverse Reactions during CAR-T Treatment

11.1 Sepsis

Diagnosis

2010 Diagnostic criteria for sepsis, severe sepsis and septic shock in Germany

I. Confirmation of infection
Diagnosis of an infection on the basis of microbiological evidence or clinical criteria

II. Systemic inflammatory host response (SIRS) (at least 2 criteria)

- Fever (≥38°C) or hypothermia (≤36°C) confirmed by rectal, intravascular or intravesical measurement
- Tachycardia: heart rate ≥90 bpm
- Tachypnea (frequency ≥20/min) or hyperventilation (PCO_2 ≤4.3 kPa/ ≤33 mmHg)
- Leukocytosis (≥12000/mm^3) or leukopenia (≤4000/mm^3) or ≥10% immature neutrophils in differential blood count

III. Acute organ dysfunction (at least 1 criterion)

- Acute encephalopathy: reduced alertness, disorientation, agitation, delirium
- Relative or absolute thrombocytopenia: decrease in platelet counts by more than 30% within 24 hours or a platelet count of less than 100.000/mm^3. Thrombocytopenia due to acute hemorrhage or immunological causes must be ruled out.
- Arterial hypoxemia: PaO_2 ≤10 kPa (≤75 mmHg) while breathing ambient air or a PaO_2/FiO_2 ratio of ≤33 kPa (≤250 mmHg) on oxygen administration. A clinically manifest heart or lung disease must be ruled out as a cause of hypoxemia.
- Renal impairment: diuresis of ≤0.5 ml/kg/h for at least 2 hours despite adequate volume resuscitation and/or an increase in serum creatinine level to > twice the upper limit of normal (ULN).
- Metabolic acidosis: Base excess of ≤–5 mmol/L or lactate concentration of >1.5 × ULN.

Sepsis: criteria I and II,
Severe sepsis: criteria I, II and III
Septic shock: criteria I and II, as well as a systolic arterial blood pressure of ≤90 mmHg for at least 1 hour, or mean arterial pressure of ≤65 mmHg, or the necessity of vasopressor administration to maintain a target systolic arterial pressure of ≥90 mmHg or mean arterial pressure of ≥65mmHg. Hypotension persists despite adequate volume resuscitation and cannot be explained by other causes.

2012 International Diagnostic Criteria for Sepsis

Table 1 Diagnostic criteria for sepsis

Infection, documented or suspected, and some of the following:
General variables
Fever (>38.3 °C)
Hypothermia (core temperature <36 °C)
Heart rate >90 min^{-1} or more than two SD above the normal value for age
Tachypnea
Altered mental status
Significant edema or positive fluid balance (> 20 mL/kg over 24 h)
Hyperglycemia (plasma glucose >140 mg/dL or 7.7 mmol/L) in the absence of diabetes
Inflammatory variables
Leukocytosis (WBC count >12,000 μL^{-1})
Leukopenia (WBC count <4,000 μL^{-1})
Normal WBC count with greater than 10 % immature forms
Plasma C-reactive protein more than two SD above the normal value
Plasma procalcitonin more than two SD above the normal value
Hemodynamic variables
Arterial hypotension (SBP <90 mmHg, MAP <70 mmHg, or an SBP decrease >40 mmHg in adults or less than two SD below normal for age)
Organ dysfunction variables
Arterial hypoxemia (PaO_2/FiO_2 <300)
Acute oliguria (urine output <0.5 mL kg^{-1} h^{-1} for at least 2 h despite adequate fluid resuscitation)
Creatinine increase >0.5 mg/dL or 44.2 μmol/L
Coagulation abnormalities (INR >1.5 or aPTT >60 s)
Ileus (absent bowel sounds)
Thrombocytopenia (platelet count <100,000 μL^{-1})
Hyperbilirubinemia (plasma total bilirubin >4 mg/dL or 70 μmol/L)
Tissue perfusion variables
Hyperlactatemia (>1 mmol/L)
Decreased capillary refill or mottling

2012 International Diagnostic Criteria for Severe Sepsis

Severe sepsis definition = sepsis-induced tissue hypoperfusion or organ dysfunction (any of the following thought to be due to the infection)
Sepsis-induced hypotension
Lactate above upper limits laboratory normal
Urine output <0.5 mL kg^{-1} h^{-1} for more than 2 h despite adequate fluid resuscitation
Acute lung injury with Pao_2/Fio_2 <250 in the absence of pneumonia as infection source
Acute lung injury with Pao_2/Fio_2 <200 in the presence of pneumonia as infection source
Creatinine >2.0 mg/dL (176.8 μmol/L)
Bilirubin >2 mg/dL (34.2 μmol/L)
Platelet count <100,000 μL
Coagulopathy (international normalized ratio >1.5)

Therapy

A. Initial resuscitation

1. We recommend the protocolized, quantitative resuscitation of patients with sepsis-induced tissue hypoperfusion (defined in this document as hypotension persisting after initial fluid challenge or blood lactate concentration ≥ 4 mmol/L. This protocol should be initiated as soon as hypoperfusion is recognized and should not be delayed pending ICU admission. During the first 6 h, the goals of initial resuscitation of sepsis-induced hypoperfusion should include all of the following as a part of a

treatment protocol:

(a) CVP 8-12 mmHg

(b) MAP C65 mmHg

(c) Urine output ≥0.5 mL kg/ h^{-1}

(d) Superior vena cava oxygenation saturation (ScvO2) or mixed venous oxygen saturation (SvO2) 70 or 65 %, respectively.

2. During the first 6 h of resuscitation, if ScvO2 less than 70 %or SvO2 equivalent of less than 65 % persists with what is judged to be adequate intravascular volume repletion in the presence of persisting tissue hypoperfusion, then dobutamine infusion (to a maximum of 20 ug/kg. min) or transfusion of packed red blood cells to achieve a hematocrit of greater than or equal to 30 % in attempts to achieve the ScvO2 or SvO2 goal are options.

3. In patients with elevated lactate levels targeting resuscitation to normalize lactate as rapidly as possible.

B. Diagnosis

1. Cultures as clinically appropriate before antimicrobial therapy if no significant delay (45 min) in the start of antimicrobial. At least 2 sets of blood cultures (both aerobic and anaerobic bottles) be obtained before antimicrobial therapy with at least 1 drawn percutaneously and 1 drawn through each vascular access device, unless the device was recently (<48 h) inserted. Other culture specimens, including urine, cerebrospinal fluid, wounds, respiratory secretions or other body fluids that may be the source of infection, should be retained before antibiotics are used.

2. Imaging studies performed promptly to confirm a potential source of infection.

3. Use of the 1,3 b-D-glucan assay (grade 2B), mannan and anti-mannan antibody assays, if available and invasive candidiasis is in differential diagnosis of cause of infection.

C. Antimicrobial therapy

1. Administration of effective intravenous antimicrobials within the first hour of recognition of septic shock (grade 1B) and severe sepsis without septic shock as the goal of therapy.

2. Initial empiric anti-infective therapy of one or more drugs that have activity against all likely pathogens (bacterial and/or fungal or viral) and that penetrate in adequate concentrations into tissues presumed to be the source of sepsis.

3. Duration of therapy typically 7–10 days; longer courses may be appropriate in patients who have a

slow clinical response, undrainable foci of infection, bacteremia with S. aureus; some fungal and viral infections or immunologic deficiencies, including neutropenia.

4. If the patient's current clinical symptoms are determined to be caused by non-infectious factors, antibiotic treatment should be discontinued immediately.

5. Procalcitonin is not recommended as a diagnostic marker for severe sepsis. During antibiotic treatment, if no infection is found, clinicians are advised to use low procalcitonin levels as a marker for stopping empirical antibiotic treatment.

D. Infection prevention

1a. For some specific infections requiring urgent treatment, such as necrotizing fasciitis, diffuse peritonitis, cholangitis, intestinal infarction, the etiology should be identified and diagnosed as soon as possible, and the diagnosis should be completed within 6 hours of symptom onset.

1b. Determine whether there are controllable sources of infection. Control measures include drainage of abscess or local infection, debridement of necrotic tissue after infection, removal of medical devices that can cause infection, or control of the source of microbial infection.

2. When infected peripancreatic necrosis is identified as a potential source of infection, definitive intervention is best delayed until adequate demarcation of viable and nonviable tissues has occurred.

3. When source control in a severely septic patient is required, the effective intervention associated with the least physiologic insult should be used (e.g., percutaneous rather than surgical drainage of an abscess.

4. If intravascular access devices are a possible source of severe sepsis or septic shock, they should be removed promptly after other vascular access has been established
(UG).

E. Fluid therapy of severe sepsis

1. We recommend crystalloids be used as the initial fluid of choice in the resuscitation of severe sepsis and septic shock.

2. The initial goal of fluid resuscitation is to achieve a CVP of at least 8 mmHg (12 mmHg for mechanical ventilation patients), and further fluid therapy is usually required.

3a. We recommend that a fluid challenge technique be applied wherein fluid administration is continued as long as there is hemodynamic improvement either based on dynamic (e.g., change in pulse pressure, stroke volume variation) or static (e.g., arterial pressure, heart rate) variables.

3b. An initial fluid challenge in patients with sepsis-induced tissue hypoperfusion with suspicion of hypovolemia to achieve a minimum of 30 mL/ kg of crystalloids (a portion of this may be albumin equivalent). More rapid administration and greater amounts of fluid may be needed in some patients

F. Vasopressors

1. recommend that vasopressor therapy initially target MAP of ≥65mmHg.

2. norepinephrine as the first-choice vasopressor, epinephrine (added to and potentially substituted for norepinephrine) when an additional agent is needed to maintain adequate blood pressure. Vasopressin (up to 0.03 U/min) can be added to norepinephrine with the intent of raising MAP to target or decreasing norepinephrine dosage.

3. Use of low-dose dopamine as a renal protective drug is not recommended.

4. All patients requiring vasopressors have an arterial catheter placed as soon as practical if resources are available.

G. Inotropic therapy

1. In patients with myocardial dysfunction (elevated filling pressure and decreased cardiac output) or persistent insufficiency of perfusion, intravenous dobutamine or vasopressin are administered even if the blood volume is adequate and the mean arterial pressure is greater than or equal to 65 mmHg.

2. We recommend against the use of a strategy to increase cardiac index to predetermined supranormal levels.

H. Corticosteroids

1. We suggest not using intravenous hydrocortisone as a treatment of adult septic shock patients if adequate fluid resuscitation and vasopressor therapy are able to restore hemodynamic stability (see goals for Initial Resuscitation). If this is not achievable, we suggest intravenous hydrocortisone alone at a dose of 200 mg per day.

2. We suggest not using the ACTH stimulation test to identify the subset of adults with septic shock who should receive hydrocortisone (2B).

3. If hydrocortisone cannot be obtained, and the replacement hormone preparation has no significant corticosteroid activity, it is recommended to increase the daily oral fluorocortisone (50 ug). If hydrocortisone is used, fluorocortisone is optional.

4. We suggest that clinicians taper the treated patient from steroid therapy when vasopressors are no longer required.

5. It is recommended that the daily dose of glucocorticoid in severe sepsis or septic shock patients should not exceed 300 mg equivalent of hydrocortisone.

6. We recommend that corticosteroids not be administered for the treatment of sepsis in the absence of shock. When low-dose hydrocortisone is given, we suggest using continuous infusion rather than repetitive bolus injections.

I. Blood product administration

1. Once tissue hypoperfusion has resolved and in the absence of extenuating circumstances, such as myocardial ischemia, severe hypoxemia, acute hemorrhage, or ischemic coronary artery disease, we recommend that red blood cell transfusion occur when the hemoglobin concentration decreases to 7.0 g/dL to target a hemoglobin concentration of 7.0–9.0 g/dL in adults.

2. We recommend not using erythropoietin as a specific treatment of anemia associated with severe sepsis.

3. We suggest that fresh frozen plasma not be used to correct laboratory clotting abnormalities in the absence of bleeding or planned invasive procedures.

4. We recommend against antithrombin administration for the treatment of severe sepsis and septic shock.

5. In patients with severe sepsis, we suggest that platelets be administered prophylactically when counts are <10,000/mm^3 (10 ×10^9/L) in the absence of apparent bleeding, as well when counts are B20,000/mm^3 (20 ×10^9/L) if the patient has a significant risk of bleeding. Higher platelet counts ≥50,000/mm^3 (50 ×10^9/L) are advised for active bleeding, surgery, or invasive procedures.

11.2 Tumor lysis syndrome

Diagnosis

**Laboratory tumor lysis syndrome**（LTLS）：2 or more
• uric acid > 8 mg/dL or 25% increase
• potassium > 6 meq/L or 25% increase
• phosphate > 4.5 mg/dL or 25% increase
• calcium < 7 mg/dL or 25% decrease

note：abnormality in two or more of the following, occurring within three days before or seven days after chemotherapy.

Clinical tumor lysis syndrome（CTLS）：It meets the diagnostic criteria of LTLS, and one or more criteria can be diagnosed.

• increased serum creatinine (1.5 times upper limit of normal)
• cardiac arrhythmia or sudden death
• seizure

note：

1. Exclude the above changes caused by side effects of drugs;

2. Definition of normal upper limit of serum creatinine (ULN): 61.6 mmol/L for patients aged 1 to 12, 88 mmol/L for patients aged 12 to 16, 114.4 mmol/L for males aged 16 and 105.6 mmol/L for females.

Cairo-Bishop Grading Classification of TLS

Table 2. **Cairo-Bishop Grading Classification of Tumor Lysis Syndrome**

	Grade 0	Grade I	Grade II	Grade III	Grade IV	Grade V
LTLS	Negative	Positive	Positive	Positive	Positive	Positive
Creatinine	≤ 1.5× ULN	1.5× ULN	> 1.5-3× ULN	> 3-6× ULN	> 6× ULN	Death[a]
Cardiac arrhythmia	None	Intervention not indicated	Nonurgent, intervention indicated	Symptomatic and incompletely controlled medically or controlled with device (AED)	Life-threatening (arrhythmia associated with heart failure, hypotension, shock)	Death[a]
Seizure	None	-	One brief generalized seizure, well controlled by anticonvulsants, or infrequent focal motor seizures not interfering with ADLs	Seizure in which consciousness is altered; poorly controlled with breakthrough generalized seizures despite intervention	Seizure of any kind that is prolonged, repetitive, or difficult to control (status epilepticus)	Death[a]

Note. LTLS = laboratory tumor lysis syndrome; ULN = upper limit of normal; AED = automated external defibrillator; ADLs = activities of daily living. Adapted from Cairo & Bishop (2004) with permission from Wiley, Inc.
[a]Death attributed to clinical tumor lysis syndrome.

Prevention

	Low-risk disease	Intermediate-risk disease	High-risk disease
Diagnostic measures	• No specific measures	• Daily monitoring of laboratory abnormalities before and during the first 7 days of anticancer therapy	• At least twice daily monitoring of laboratory abnormalities before and during the first 7 days of anticancer therapy
Preventive measures	• Moderate hydration is recommended	• Vigorous hydration<br>• Keep urinary output >100 mL/h<br>• Treatment with allopurinol or febuxostat should be started at least 24 hours before initiation of anticancer therapy and should be continued till normalization of uric acid levels and signs of large tumor burden are absent	• Vigorous hydration<br>• Keep urinary output >100 mL/h<br>• Single dose 6 mg of rasburicase. Repeat doses as necessary. In case of contraindication treatment with febuxostat
Treatment of established tumor lysis syndrome	• Admission to intensive care unit with continuous cardiac monitoring and monitoring of laboratory abnormalities every 4–6 hours<br>• Early nephrology consultation to estimate the indications for renal replacement therapy<br>• Correction of electrolyte abnormalities<br>• Vigorous hydration, keep urinary output >100 mL/h<br>• Single dose 6 mg of rasburicase. Repeat doses as necessary. In case of contraindication, treatment with febuxostat		

Therapy

3.1Intravenous hydration and Urinary alkalinization

Aggressive intravenous hydration at a rate of 2 to 3 L/day, maintaining a urine output of 100 to 200 mL/ hour. Mannitol 0.5 mg/kg or furosemide 0.5-1.0 mg/kg can be used as urine agent. The dosage was 2-4 mg/kg when hypouria or anuria occures, and the relative density of urine was 1.010-1.015.

Treating Electrolyte disorders

(1) Hyperphosphatemia

Phosphate concentration ≥2.1mmol/L(children) or ≥1.45mmol/L(adult).

Phosphate should be avoided. Aluminum hydroxide can be administered 15 mL/time and orally 50-100 mg/(kg.d) four times a day. Severe illness: dialysis, continuous arteriovenous hemofiltration and continuous venovenous hemofiltration (CVVH) can be used.

(2) Hypocalcemia: ≤1.75mmol /L.

Asymptomatic patients: no treatment for the time being;

Symptomatic patients: intravenous injection of calcium gluconate 50-100 mg/kg.

(3) Hyperkalemia: ≥6.0mmol/L.

Asymptomatic patients: oral or intravenous potassium supplementation should be avoided; ECG or ECG monitoring; sodium polystyrene sulfonate can be given (1 g/kg oral or enema).

Severe illness: When serum potassium is more than 7.0 mmol/L, in addition to the above methods, 10% calcium gluconate 100-200 mg/kg intravenous injection, 25% glucose (2mL/kg) +insulin 0.1U/kg intravenous drip, or even hemodialysis can be used.

(4) hyperuricemia：Uric acid≥476mol/L,occurs 48-72 h post chemotherapy

3.2 Allopurinol:

Adults were given 100 mg/m^2 or 10 mg/(kg.d) orally three times daily (maximum dose 800 mg/d) or 200-400 mg/(m^2.d) or intravenously injected 3 times daily (maximum dose 600 mg/d).

300-450 mg/ (m^2.d) was given orally three times daily to children, the maximum dose was 400 mg/ (m^2.d).

Reduced dose in patients with renal failure,200 mg/d when creatinine clearance is 0.33-0.17 mL/s；100 mg/d when creatinine clearance is less than 0.17 mL/s.

When allopurinol is applied, the dosage of 6-MP or MP should be reduced to 65%-75%.

3.3 Indicators of Allopurinol in the Treatment of Hyperuricemia:

A. Normal uric acid;

B. The types of tumors were Hodgkin's lymphoma and chronic myeloid leukemia without blood invasion.

C. Tumor load is small, such as white blood cell count (WBC) less than 50 *109/L and lactate dehydrogenase (LDH) less than 2 times normal.

D. Chemotherapy intensity is low.

E. Tumors have no renal infiltration.

3.4 Treatment of renal failure (uremia)

Monitor fluid intake, electrolyte and blood pressure. Treatment of hyperuricemia and hyperphosphatemia, prevention and treatment of uric acid nephropathy, adjust the dose of renal excretion drugs. If necessary, dialysis (blood-peritoneum) or blood purification (CAVH, CVVH) were performed.

Supplementary References

1. Lee DW, Gardner R, Porter DL, et al. Current concepts in the diagnosis and management of cytokine release syndrome. Blood 2014;124:188-95.
2. Institute NC. Common Terminology Criteria for Adverse Events version 4.03. US Department of Health and Human Services. 2010.
3. Kumar S, Paiva B, Anderson KC, et al. International Myeloma Working Group consensus criteria for response and minimal residual disease assessment in multiple myeloma. Lancet Oncol 2016;17:e328-e46
