# Supplementary material for: Bispecific CAR T cell therapy targeting BCMA and CD19 in relapsed/refractory multiple myeloma: a phase I/II trial
Source: Nat Commun. 2024 Apr 20;15:3371. doi: 10.1038/s41467-024-47801-8 (PMC11032309; doi:10.1038/s41467-024-47801-8)
Supplement: Supplementary file 3 — Reporting Summary [file 41467_2024_47801_MOESM3_ESM.pdf]

nature portfolio

Corresponding author(s): Jiang Cao

Last updated by author(s): Oct 14, 2023

Reporting Summary

Nature Portfolio wishes to improve the reproducibility of the work that we publish. This form provides structure for consistency and transparency in reporting. For further information on Nature Portfolio policies, see our Editorial Policies and the Editorial Policy Checklist.

Statistics

For all statistical analyses, confirm that the following items are present in the figure legend, table legend, main text, or Methods section.

n/a	Confirmed	
☐	☒	The exact sample size (*n*) for each experimental group/condition, given as a discrete number and unit of measurement
☐	☒	A statement on whether measurements were taken from distinct samples or whether the same sample was measured repeatedly
☐	☒	The statistical test(s) used AND whether they are one- or two-sided<br>*Only common tests should be described solely by name; describe more complex techniques in the Methods section.*
☒	☐	A description of all covariates tested
☒	☐	A description of any assumptions or corrections, such as tests of normality and adjustment for multiple comparisons
☐	☒	A full description of the statistical parameters including central tendency (e.g. means) or other basic estimates (e.g. regression coefficient) AND variation (e.g. standard deviation) or associated estimates of uncertainty (e.g. confidence intervals)
☐	☒	For null hypothesis testing, the test statistic (e.g. *F*, *t*, *r*) with confidence intervals, effect sizes, degrees of freedom and *P* value noted<br>*Give P values as exact values whenever suitable.*
☒	☐	For Bayesian analysis, information on the choice of priors and Markov chain Monte Carlo settings
☒	☐	For hierarchical and complex designs, identification of the appropriate level for tests and full reporting of outcomes
☒	☐	Estimates of effect sizes (e.g. Cohen's *d*, Pearson's *r*), indicating how they were calculated

Our web collection on statistics for biologists contains articles on many of the points above.

Software and code

Policy information about availability of computer code

Data collection	RTCA Software 2.0.0.1301 was used for RTCA experiment<br>BD FACS Diva SoftWare v9.2 is used for flow cytometry<br>IndiGO 2.0.3.0 is used for live imaging of small animals
Data analysis	SAS, Version 9.4 is used for clinical data; For flow cytometry: FlowJo v.10 software; For in vivo imaging: IndiGO 2.0.3.0 ; For data analysis: GraphPad Prism 10 and SPSS 26.

For manuscripts utilizing custom algorithms or software that are central to the research but not yet described in published literature, software must be made available to editors and reviewers. We strongly encourage code deposition in a community repository (e.g. GitHub). See the Nature Portfolio guidelines for submitting code & software for further information.

Data

Policy information about availability of data

All manuscripts must include a data availability statement. This statement should provide the following information, where applicable:

- Accession codes, unique identifiers, or web links for publicly available datasets
- A description of any restrictions on data availability
- For clinical datasets or third party data, please ensure that the statement adheres to our policy

Aggregated data and associated supporting documents (e.g., protocol) will be made available upon request. Individual participants data that underlie the results

reported in this article, after deidentification, will be shared upon request after publication and ending 36 months following article publication to researchers who provide a methologically sound proposal. Proposals should be directed to the corresponding author, Jiang Cao. All remaining data can be found in the Article, Supplementary, and Source Data files. Source data are provided with this paper.

Research involving human participants, their data, or biological material

Policy information about studies with human participants or human data. See also policy information about sex, gender (identity/presentation), and sexual orientation and race, ethnicity and racism.

Reporting on sex and gender	Sex and gender were not considered in the study design. Sex/gender analysis carried out are described in the protocol.
Reporting on race, ethnicity, or other socially relevant groupings	Race, ethnicity, or other socially relevant groupings were not considered in the study design.
Population characteristics	To be eligible for participation in this study, patients had to be: 1) Less than 70 years old; 2) Meet the diagnostic criteria for R/R MM defined by the International Myeloma Working Group (IMWG) ; 3) Patients had experienced relapse or were refractory to at least 2 prior lines of therapy, including a proteasome inhibitor and an immunomodulatory drug; and 4) Patients had measurable disease and adequate performance status and organ function, with an Eastern Cooperative Oncology Group (ECOG) score ≤ 2. Positive BCMA expression on MM cells was required to be confirmed by flow cytometry regardless of whether CD19 was expressed, but no pre-specified level of expression was required. Female patients had to be human chorionic gonadotropin-negative, with no plans for pregnancy within 6 months of treatment. Patients with mental or psychological illnesses, severe allergies, or a history of severe allergies (especially those who were allergic to interleukin [IL]-2) were excluded. Detailed inclusion and exclusion criteria are provided in the supplementary information. The median age of patients was 57 years (range 31-70), and the median time from MM diagnosis to CAR T cell infusion was 29.5 months (range 4-162). A total of 46 patients (96%) had stage II or III disease, 7 patients (14%) had extramedullary disease, and 34 patients (68%) had high-risk cytogenetic profiles, defined by the presence of del(17p), t(4;14), or t(14;16). Patients had a median of 4 lines (range, 2 to 11) of therapy before enrolment. Among them, 20 patients (40%) had previously received auto-HSCT, and 5 patients (10%) had received prior BCMA, CD19 or GPRC5D-targeted CAR T cell treatment.
Recruitment	Healthy participants were recruited through public recruitment advertisement which was approved by the Affiliated Hospital of Xuzhou Medical University. Fresh PBMCs from healthy donors were provided by the Affiliated Hospital of Xuzhou Medical University. The recruitments of healthy human blood donors were approved by the Clinical Research Ethics Committee of the Affiliated Hospital of Xuzhou Medical University. All the participants signed the Informed Consent Form.All patients who are receiving BC19 CAR T cell therapies are seen in the cell therapy service at the institution. All eligible patients were approached for the study by treating investigators. Once consented, patients underwent screening evaluations as specified in the protocols. Participants were recruited by the investigators pursuant to IRB approved methods to reduce the possibility of biased recruiting.
Ethics oversight	The clinical trial (ChiCTR2000033567) was approved by the The Medical Ethics Committee of the Affiliated Hospital of Xuzhou Medical University, the name of the Medical Ethics is "The Medical Ethics Committee of the Affiliated Hospital of Xuzhou Medical University" . All patients enrolled and treated in this trial signed written informed consents before participation. All clinical investigations were conducted according to the Declaration of Helsinki principles.

Note that full information on the approval of the study protocol must also be provided in the manuscript.

Field-specific reporting

Please select the one below that is the best fit for your research. If you are not sure, read the appropriate sections before making your selection.

- [x] Life sciences
- [ ] Behavioural & social sciences
- [ ] Ecological, evolutionary & environmental sciences

For a reference copy of the document with all sections, see nature.com/documents/nr-reporting-summary-flat.pdf

Life sciences study design

All studies must disclose on these points even when the disclosure is negative.

Sample size	From June 2020 to February 2022, 64 patients with R/R MM were screened for eligibility, and 54 patients were initially enrolled and underwent leukapheresis. The manufacturing of BC19 CAR T cells was successful for 100% of patients. Four patients discontinued treatment due to rapid disease progression prior to infusion (Supplementary Fig. 2). Fifty patients finally received BC19 CAR T cell infusions, and the patients' baseline characteristics are listed in Table 1. No statistical methods were used to pre-determine sample size. The number of samples is determined based on previous clinical research experience and actual collection conditions, and can also meet statistical requirements.
Data exclusions	No patient data from the enrolled patients are excluded from the study report.
Replication	Sample sizes of patients in each figures and tables are noted to indicate the degree of replication in the results.
Randomization	It is a single arm study.
Blinding	It is an open-lable study.

Reporting for specific materials, systems and methods

We require information from authors about some types of materials, experimental systems and methods used in many studies. Here, indicate whether each material, system or method listed is relevant to your study. If you are not sure if a list item applies to your research, read the appropriate section before selecting a response.

Materials & experimental systems

n/a	Involved in the study
☐	☒ Antibodies
☐	☒ Eukaryotic cell lines
☒	☐ Palaeontology and archaeology
☐	☒ Animals and other organisms
☐	☒ Clinical data
☒	☐ Dual use research of concern
☒	☐ Plants

Methods

n/a	Involved in the study
☒	☐ ChIP-seq
☐	☒ Flow cytometry
☒	☐ MRI-based neuroimaging

Antibodies

Antibodies used	Biotinylated protein L (Acro Biosystems, Cat: RPL-P814R, Lot: BL11R-76EF1-GY,1:400 dilution) and APC--conjugated Streptavidin (Biolegend, Cat: 405207, Lot: B388667, 1:100 dilution). PE-labeled anti-CD45RA (Biolegend, Clone: HI100, Cat: 304107, Lot: B378521, 1:100 dilution) and PE-Cy7-labeled anti-CD62L (Biolegend, Clone: DREG-56, Cat: 304821, Lot: B373156, 1:100 dilution); CellTracker Deep Red (Thermo Fisher Scientific, Cat: C34565, Lot: 1987253, CTDR, 1:40000 dilution).
Validation	All antibodies used in this study are commercially available. Antibody validations were performed by the suppliers and the information is provided on the website and product information datasheets. The certificate of analysis (CoA) was provided for the quality assurance of each antibody lot

Eukaryotic cell lines

Policy information about cell lines and Sex and Gender in Research

Cell line source(s)	Human B cell precursor leukemia cell line Nalm6, human myeloma cell line U266 and human ovarian cancer cell line SKOV3 are obtained from ATCC and cultured according to standard protocols. Nalm6 or U266 cells stably expressing luciferase (Nalm6-luc or U266-luc) and SKOV3 cells stably expressing CD19 (SKOV3-CD19) or BCMA (SKOV3-BCMA) were constructed in our lab.
Authentication	All cell lines' authenticity was confirmed through STR (Short Tandem Repeat) profiling, and routine mycoplasma testing was performed using a mycoplasma detection kit.
Mycoplasma contamination	All cell lines used tested negative for mycoplasma contamination.
Commonly misidentified lines (See ICLAC register)	No commonly misidentified cell lines from the ICLAC Register were used in the study.

Animals and other research organisms

Policy information about studies involving animals; ARRIVE guidelines recommended for reporting animal research, and Sex and Gender in Research

Laboratory animals	For establishing MM xenograft mouse models, 6- to 8-week-old male NCG (NOD/ShiLtJGpt-Prkdcem26Cd52Il2rgem26Cd22/Gpt) mice were purchased from GemPharmatech Co., Ltd with a production license number SCXK (Su) 2023-0009. The mice were maintained in a specific pathogen-free (SPF) environment, in accordance with the requirements of the Ethics Committee for Experimental Animals of Xuzhou Medical University. They were subjected to a 12-hour light-dark cycle, provided ad libitum access to food and water, and allowed to acclimate for one week.
Wild animals	Wild animals are not used in this study.
Reporting on sex	The study doesn't focus on sex differences.
Field-collected samples	The study did not involve samples collected from the field.
Ethics oversight	This study was approved by the Ethics Committee for Experimental Animals of Xuzhou Medical University (Number: XYFY2020-KL062-01). The mice were maintained in a specific pathogen-free (SPF) environment, in accordance with the requirements of the Ethics Committee for Experimental Animals of Xuzhou Medical University.

Note that full information on the approval of the study protocol must also be provided in the manuscript.

Clinical data

Policy information about clinical studies

All manuscripts should comply with the ICMJEguidelines for publication of clinical research and a completedCONSORT checklist must be included with all submissions.

Clinical trial registration	ChiCTR2000033567
Study protocol	Full study protocol is included with the manuscript submission.
Data collection	Between June 5, 2020, and February 28, 2022, 50 patients with R/R MM were finally underwent BC19 CAR-T therapy and data collection. Clinical data was collected in the Affiliated Hospital of Xuzhou Medical University.
Outcomes	The primary objective of this study was to determine the safety and tolerability of the BC19 CAR-T, and the secondary objective was to determine the effecy of the BC19 CAR-T. All 50 patients had hematological adverse events, including neutropenia in 100% of the patients, leukopenia in 100%, anemia in 94% and thrombocytopenia in 88%. Grade 3-4 hematological adverse events were neutropenia (49 [98%] of 50 patients), leukopenia (48 [96%]), thrombocytopenia (33 [66%]), and anemia (32 [64%]). Of the 50 patients assessable for efficacy, 46 (92%; 95% CI, 81 to 98) achieved an overall response (PR or better) to the BC19 CAR T cells, including 19 (38%) sCRs, 12 (24%) CRs, 8 (16%) VGPRs, and 7 (16%) PRs (Table 2). Four patients (8%) had stable disease as best response. The median time to first PR or better was 23.5 days (range, 14-30), and the median time to best response was 1.9 month (range, 0.5-6.1).

Plants

Seed stocks	Plant is not involved in this study.
Novel plant genotypes	Plant is not involved in this study.
Authentication	Plant is not involved in this study.

Flow Cytometry

Plots

Confirm that:

- [x] The axis labels state the marker and fluorochrome used (e.g. CD4-FITC).
- [x] The axis scales are clearly visible. Include numbers along axes only for bottom left plot of group (a 'group' is an analysis of identical markers).
- [x] All plots are contour plots with outliers or pseudocolor plots.
- [x] A numerical value for number of cells or percentage (with statistics) is provided.

Methodology

Sample preparation	Primary peripheral blood mononuclear cells (PBMCs) were isolated from peripheral blood of the healthy donor and the patients with R/R MM. T lymphocytes were isolated using EasySepTM human T Cell Isolation Kit (STEMCELL) according to the manufacture's instruction,1×10^7 human T cells were infected by the lentivirus mentioned above. 1×10^6 CAR-T were used for Flow Cytometry.
Instrument	Samples were measured on a Canto II (BD Biosciences, Heidelberg, Germany)
Software	NovoExpress Version 1.2.5
Cell population abundance	These cells were not sorted
Gating strategy	The cells were identified by their FSC and SSC profiles. A polygon gate was drawn on a FSC-A vs SSC-A dot plot to include the cell population. Aggregates were identified and removed from the analyzed population by FSC-A vs FSC-H doublet discrimination gates. The boundaries between positive and negative gates were set based upon an unstained control.

- [x] Tick this box to confirm that a figure exemplifying the gating strategy is provided in the Supplementary Information.
